# Qualitative and quantitative research on preferences and perceptions regarding HIV post-exposure prophylaxis among young women, men, female sex workers, members of the LGBTQ + community and people who inject drugs in Kenya, Nigeria and Zimbabwe

**DOI:** 10.3389/frph.2025.1606013

**Published:** 2025-10-09

**Authors:** Moushira El-Sahn, Rose Elliott, Mona El-Sahn, Ignacio Garcia-Gurtubay, Karen Kong, Trisha Wood Santos, Raphael Mulwa, Elizabeth Muthoka, Jeff Lucas

**Affiliations:** ^1^ Routes2Results, London, United Kingdom; ^2^ Trisha Wood Santos Consulting, LLC, Seattle, WA, United States; ^3^ Infotrak Research & Consulting Limited, Nairobi, Kenya

**Keywords:** HIV prevention, health-disparate minority and vulnerable populations, sexual and gender minorities, drug users, sex workers, transgender persons, WHO

## Abstract

**Introduction:**

The World Health Organization (WHO) recently updated its guidelines for HIV post-exposure prophylaxis (PEP). These guidelines recommend community delivery and task-sharing for PEP administration and suggest enhanced adherence counseling for those who initiate PEP. This work provides insights into considerations for optimizing people's knowledge, demand for, use of and adherence to PEP through new channels. This mixed-method study examined five research questions concerning the perceptions and experiences of various groups regarding accessing and utilizing PEP and explored opinions on alternate delivery options to broaden access.

**Methods:**

The total number of end-users interviewed for this study via qualitative and quantitative interview methodologies was 1,156. We spoke with a total of 236 end-users through qualitative Focus Groups (FGs) and in-depth interviews (IDIs) and surveyed 920 end-users with a questionnaire in Kenya, Nigeria and Zimbabwe, including members of key populations (Female Sex Workers, Members of the LGBTQ + Community, People Who Inject Drugs).

**Results:**

Prompted awareness of PEP varied across the study countries (56.2% overall). Healthcare providers (doctors and nurses) were cited by end-user respondents as primary sources of information on HIV and PEP. PEP information evaluation revealed that condomless sex or condom malfunction were the emergency situations that resonated most with end-users. The majority (86.4%) cited a perceived likelihood to use PEP if exposed to HIV. A general hospital was deemed most acceptable for PEP access by end-users in all three countries (81.2%); clinical routes were perceived as preferable in terms of broadening access to PEP, with key reasons being convenience (68.2%), trustworthiness (56.5%) and knowledge (56.5%). End-users emphasized the need for consistent, correct, and supportive interaction points with healthcare providers to complete PEP treatment and follow-up.

**Conclusions:**

WHO's recent update to the PEP guidelines recommends community-based distribution and task-sharing of PEP. Uptake is dependent on confidentiality and privacy of services as well as on increasing awareness and knowledge of the PEP pathway. Provision of PEP by healthcare providers needs to incorporate multiple end-user touch/access-points with emotional support for greater adherence, and our study highlights the different preferences and access contexts among end-users for PEP.

## 1 Introduction

The World Health Organization (WHO) recently updated its HIV post-exposure prophylaxis (PEP) guidelines to support increased access to PEP (1), with recommendations for community delivery and task-sharing for PEP administration and suggestion of enhanced adherence counseling for those who initiate PEP (2, 3). Studies have shown that end-user and provider awareness, usage of and adherence to PEP could be optimized (4–8).

A 2014 systematic review and meta-analysis of 97 studies on PEP adherence in multiple countries (based on 21,462 PEP initiations) found that non-occupational exposure was the main reason for PEP initiation in 34 studies, occupational exposure in 22 studies, and sexual assault in 26 studies; the remainder (15 studies) reported mixed exposures (9).

Current research indicates a concerted effort is needed to increase awareness of PEP, particularly among the general population and among key populations at risk of contracting HIV who face barriers to accessing HIV-related healthcare. Additionally, efforts should focus on broadening access to PEP and improving users' adherence by understanding the barriers they face and identifying effective strategies to enhance adherence (9–11).

The objectives of this research were to answer five research questions: (1) What is the awareness of PEP among end-users? (2) What are end-users' reactions to PEP information, and what is the perceived likelihood to use PEP? (3) What are the preferred access points for PEP? (4) What do end-users think about the PEP Pathway? (5) What could broadening access look like? Table 1 illustrates specific objectives achieved in this study and how our results are organized.

**Table 1:** Research objectives and corresponding outcome measures.

Objectives	
1. Awareness and information sources	• HIV prevention methods
	• Awareness of PEP
	• HIV/PEP: information sources
2. Reactions to PEP	• PEP profile evaluation
	• Likelihood to use PEP
	• Anticipated access
3. PEP pathway	• Evaluation of specific steps in the PEP pathway – what was offered/provided and user-perceived difficulty at each step
4 and 5. Preferred access points and broadening access to PEP	• Settings deemed acceptable/preferable for PEP access

HIV, human immunodeficiency virus; PEP, post-exposure prophylaxis.

## 2 Methods

We employed a mixed-method approach whereby the qualitative and quantitative arms of the research were run simultaneously in Kenya (Nairobi and Mombasa), Nigeria (Lagos and Abuja) and Zimbabwe (Harare and Bulawayo), interviewing potential and experienced end-users (herein all described as “end-users”). The qualitative research comprised in-person in-depth interviews (IDIs) and focus groups (FGs) and the quantitative research was conducted utilizing computer-assisted in-person interviews (CAPI). Respondents were not able to take part in both the qualitative and quantitative phases. All interviews were conducted in safe, confidential (as far as possible, with precautions taken against being overheard) and comfortable locations of the respondents' choice or with respondent approval. All research received ethical approval from in-country local Institutional Research Boards (IRBs). The fieldwork was conducted between October 2023 and January 2024.

### 2.1 Shared methodology: recruitment method

The recruitment process was consistent across all three countries. Qualitative data were collected via IDIs and FGs. Respondents were recruited using a mixed-sex team of recruiters with screening questionnaires to determine eligibility (programmed and conducted on CAPI devices), which they conducted face-to-face with potential end-users who lived in the target areas. Recruitment teams selected low-income areas within each city, where respondents were recruited from their households, chosen at random through walking (from a landmark such as a clinic, religious building, police station or gas station) or hotspots (such as universities, social spaces and sports venues) whereby the teams attempted door-to-door screening with skips between houses and varied pre-selected locations to ensure a wider recruitment field.

Households to be interviewed were selected using a household selection grid. Upon arrival at the sampled enumeration area, the supervisor identified a starting point, typically a street or a conspicuous landmark within the area. From the landmark, the enumerators randomly selected the first household and, using the skip interval and left-hand (anti-clockwise) rule, sampled additional households for inclusion in the survey. A skip interval of four households was applied, while in areas with flats, only one interview was conducted per flat.

To ensure ethical and effective engagement with hard-to-reach populations, particularly MSM, we employed tailored recruitment strategies through trusted community-based umbrella organizations such as NGOs in Kenya (GALCK+), Zimbabwe (GALZ), and Nigeria (Heartland Alliance), and adapted research materials to suit the contextual needs and preferences of each group. Female Sex Workers were recruited using a snowballing approach at their workplaces, such as nightclubs, bars, and red-light districts. A recruitment factor of three was applied, meaning each participant could refer a maximum of two additional respondents.

Table 2 outlines the recruitment process for each group of participants among the end-user sample. The target population self-reported as sexually active (apart from People Who Inject Drugs), HIV negative and in Socio-Economic Class (SEC) C-D. This research utilized the EquityTool which is a short, country-specific questionnaire to measure relative wealth (12). SEC strata C and D were selected for this research as they encompass the broadest and largest section of the population.

**Table 2:** Recruitment process for each group of participants.

Men and YW	• Conducted random walk and hotspots (schools, universities, sports venues), targeted and snowballing approach.
MSM	• Connected with MSM NGO support groups who worked directly with and for the benefit of MSM in Kenya (GALCK+), Zimbabwe (GALZ) and Nigeria (Heartland Alliance).
FSW	• Utilised NGO groups where relevant for example CeSHHAR in Zimbabwe.
	• Recruitment conducted at their places of work
	• Potential for late afternoon and evening interviewing.
	• ·One participant had the potential to recruit up to a maximum of 2 participants to the study.
TGD (Qual)	• We partnered with the Bill & Melinda Gates Foundation and their network of TGD organisations in Kenya for recruitment.
(Kenya only)	
PWID (Qual)	• We followed local partner recommendation to recruit for this type of respondent in Mombasa.
(Kenya only)	

CeSHHAR, Centre for Sexual Health and HIV/AIDs Research; FSW, female sex workers; GALCK, gay and lesbian coalition of Kenya; GALZ, gay and lesbians of Zimbabwe; MSM, men who have sex with men; NGO, non-governmental organization, NE, non-PEP experienced; PE, PEP-experienced; PWID, People Who Inject Drugs; TGD, transgender and gender diverse, YW, young women.

The age ranges selected for both the qualitative and quantitative sample differed depending on the population to ensure adequate representation of that specific group. Young Women had the lowest age range of 18–24 years, Men/Men who have Sex with Men were aged 18–40 years and the remaining three respondent types, Female Sex Workers, Transgender and Gender Diverse people, and People Who Inject Drugs, were aged 18–30 years (see Table 3). Young Women between the ages of 18–24 were chosen to represent the younger age groups who experience the greatest incidence of HIV infection (13). People Who Inject Drugs and Transgender and Gender Diverse people only took part in the qualitative part of the study.

**Table 3:** Summary of screening criteria for recruitment.

End-users: Young Women
• Socio-economic measure C1-D
• Aged 18–24
• Must be sexually active
• Soft quota: *n*=5 to have experience of using PEP
• HIV status must be negative
End-users: Men
• Socio-economic measure C1-D
• Aged 18–40
• Must be sexually active
• Soft quota: *n*=5 to have experience of using PEP
• HIV status must be negative
End-users: Men who have Sex with Men (MSM)
• Socio-economic measure C1-D
• Aged 18–40
• Must be sexually active
• HIV status must be negative
End-users: Female Sex Workers
• Socio-economic measure C1-D
• Aged 18–30
• Must be sexually active
• HIV status must be negative
End-users: Transgender and Gender Diverse people (TGD)
• Socio-economic measure C1-D
• Aged 18–30
• Must be sexually active
• HIV status must be negative
End-users: People Who Inject Drugs (PWID)
• Socio-economic measure C1-D
• Aged 18–30
• HIV status must be negative

FSW, female sex workers; MSM, men who have sex with men; PEP, post-exposure prophylaxis; PrEP, pre-exposure prophylaxis; PWID, people who inject drugs; TGD, transgender and gender diverse.

The selection process for respondents varied by target group. At the household level, random selection was applied only to young women and men. The Kish Grid method was used to ensure equal probability selection at the household level. Recruiters listed all eligible household members (within the target age groups) in order of age, from oldest to youngest. The Kish Grid was integrated into a mobile data collection tool, which randomly selected the respondent to be interviewed. Recruitment locations within each region of a country were carefully sampled for both qualitative and quantitative phases to ensure comprehensive coverage and avoid double recruitment.

### 2.2 Qualitative phase

#### 2.2.1 Sample and data collection

Qualitative data were collected via IDIs and FGs. IDIs lasted 60 min and FGs lasted 120 min. We spoke with a total of 236 end-users. The breakdown of the sample can be seen in Table 4. Focus groups were carried out with men and Young Women, and face-to-face IDIs with key populations (Men who have Sex with Men, Female Sex Workers, Transgender and Gender Diverse people, People Who Inject Drugs) because of a heightened requirement for confidentiality and respondent comfort with discussing sensitive topics.

**Table 4:** Sample breakdown.

Country	Kenya		Nigeria		Zimbabwe	
Phase	Qualitative	Quantitative	Qualitative	Quantitative	Qualitative	Quantitative
Respondent	End-users (*n* = 80)	End-users (*n* = 309)	End-users (*n* = 76)	End-users (*n* = 307)	End-users (*n* = 80)	End-users (*n* = 304)
	PE (*n* = 31)	PE (*n* = 145)	PE (*n* = 22)	PE (*n* = 39^a^)	PE (*n* = 66)	PE (*n* = 120)
	NE (*n* = 49)	NE (*n* = 164)	NE (*n* = 54)	NE (*n* = 268)	NE (*n* = 14)	NE (*n* = 184)
Respondent sub-type	YW (*n* = 25, 5FGs)	YW (*n* = 101)	YW (*n* = 25, 5FGs)	YW (*n* = 102)	YW (*n* = 30, 6FGs)	YW (*n* = 100)
	Men (*n* = 25, 5FGs)	Men (*n* = 100)	Men (*n* = 30, 6 FGs)	Men (*n* = 104)	Men (*n* = 30, 6FGs)	Men (*n* = 100)
	MSM (*n* = 10)	MSM (*n* = 57)	MSM (*n* = 10)	MSM (*n* = 51)	MSM (*n* = 10)	MSM (*n* = 52)
	FSW (*n* = 10)	FSW (*n* = 51)	FSW (*n* = 11)	FSW (*n* = 50)	FSW (*n* = 10)	FSW (*n* = 52)
	PWID (*n* = 5)	–	–	–	–	–
	TGD (*n* = 5)	–	–	–	–	–
Experience level – PE = PEP experienced NE = Non PEP- experienced	YW – PE (*n* = 10, 2FGs)	YW – PE (*n* = 46^a^)	YW – PE (*n* = 15, 3FGs)	YW – PE (*n* = 12^a^)	YW – PE (*n* = 25, 5FGs)	YW – PE (*n* = 40^a^)
	YW – NE (*n* = 15, 3FGs)	YW – NE (*n* = 55)	YW – NE (*n* = 10, 2FGs)	YW – NE (*n* = 90)	YW – NE (*n* = 5, 1FG)	YW – NE (*n* = 60)
	Men – PE (*n* = 10, 2FGs)	Men – PE (*n* = 47^a^)	Men – PE (*n* = 0)	Men – PE (*n* = 16^a^)	Men – PE (*n* = 25, 5FGs)	Men – PE (*n* = 41)
	Men – NE (*n* = 15, 3FGs)	Men – NE (*n* = 53)	Men – NE (*n* = 30, 6FGs)	Men – NE (*n* = 88)	Men – NE (*n* = 5, 1FG)	Men – NE (*n* = 59)
	MSM – PE (*n* = 2)	MSM – PE (*n* = 26^a^)	MSM – PE (*n* = 4)	MSM – PE (*n* = 6^a^)	MSM – PE (*n* = 10)	MSM – PE (*n* = 20^a^)
	MSM – NE (*n* = 8)	MSM – NE (*n* = 31^a^)	MSM – NE (*n* = 6)	MSM – NE (*n* = 45)	MSM – NE (*n* = 0)	MSM – NE (*n* = 32^a^)
	FSW – PE (*n* = 5)	FSW – PE (*n* = 26^a^)	FSW – PE (*n* = 3)	FSW – PE (*n* = 5^a^)	FSW – PE (*n* = 6)	FSW – PE (*n* = 19^a^)
	FSW – NE (*n* = 5)	FSW – NE (*n* = 25^a^)	FSW – NE (*n* = 8)	FSW – NE (*n* = 45)	FSW – NE (*n* = 4)	FSW – NE (*n* = 33^a^)

FG, focus group; FSW, female sex workers; MSM, men who have sex with men; NE, non-PEP experienced; PE, PEP-experienced; PWID, people who inject drugs; TGD, transgender and gender diverse, YW, young women.

^a^ low base size.

Infotrak enlisted experienced researchers across the three countries to conduct the data collection exercise. Interviewers selected for the study were required to have two to five years of experience in household data collection, while supervisors needed a minimum of three years of experience overseeing similar exercises. The recruitment process also considered factors such as gender balance and the ability of field staff to communicate effectively with respondents.

To ensure strict adherence to study protocols, Routes2Results and Infotrak developed a comprehensive training manual detailing the procedures and responsibilities for the data collection process. The field teams in each country participated in an intensive three-day training session covering the study background, methodology, research tools (recruitment questionnaires, discussion guides, and structured questionnaires), ethical guidelines (informed consent and participant management), and data quality control measures. The training included mock interviews, role-playing exercises, and discussions on potential field challenges and mitigation strategies. Following the training, a two-day pilot exercise was conducted to familiarize the field team with questionnaire administration and refine interview techniques. Debriefing sessions were then held with both the field team and the Routes2Results (R2R) team to review experiences from the pilot exercise and fine-tune the data collection tools before proceeding with full-scale data collection.

#### 2.2.2 Data analysis

For the qualitative data, codebooks were developed iteratively following review of transcripts by the core study team of four research directors, with at least five years of qualitative analysis experience. This framework was used to code transcripts and identify key themes that emerged from the data. An iterative and systematic process of content and pattern analysis was carried out. The study team used the analytical categories developed as part of the coding framework to derive meaning from the various pieces of evidence to answer the research questions. The study analysis team met regularly to review codebook outputs, with a view to align, calibrate and/or resolve coding challenges; this included discussion and consensus-building, revisiting the codebook, and third-party review.

### 2.3 Quantitative phase

#### 2.3.1 Sample and data collection

The sample size was drawn on a stratified quota basis. Data were collected via face-to-face CAPI with a total of 920 end-users, stratified into two major cities and areas within them in Kenya (Nairobi and Mombasa), Nigeria (Lagos and Abuja) and Zimbabwe (Harare and Bulawayo) (Supplementary Table S3). Stratified quotas indicate that there was a predetermined number of study respondents to be recruited per location and category. Each study region was assigned a specific quota per respondent category to ensure adequate representation of the target respondents across the three countries.

Quota sampling was used to allocate samples for each respondent category. Surveys lasted between 35 and 48 min, with the consent of the respondents granted where interviews overran. The target population for the quantitative phase mirrored that of the qualitative phase. Other demographic information for end-users was captured during the interviews (Supplementary Table S3); this did not, however, determine eligibility.

For data collection, mobile phones/tablets with offline data storage capability were used and data were automatically uploaded when an internet connection was available. All interviews were audio-recorded, transcribed, and, where necessary, translated prior to analysis.

The research teams, comprising female interviewers and recruiters and mixed-sex supervisors, were briefed and trained. Pilot interviews were observed across all countries, ensuring adherence to objectives, processes and ethical considerations. Interviews were conducted by experienced interviewers in the local language or English, based on respondent preference.

### 2.4 Data analysis

The closed-ended quantitative data were analyzed using International Business Machines (IBM)'s Statistical Package for the Social Sciences (SPSS). Data were initially analyzed by total base size and were then further analyzed after division into PEP-experienced and non-experienced respondents, and, for end-users, into specific populations. Results from Young Women and Men are presented separately for each country; although results from the Female Sex Worker and Men who have Sex with Men samples are also presented separately, these are indicative findings because of the low sample size.

The clean dataset was coded prior to statistical analysis. All statistical analysis was undertaken using SPSS. Advanced analytical tools and approaches were used to make inferences about the target populations. Statistical adjustments of the data were applied where necessary. In accordance with the conventional acceptance of statistical significance at a *P*-value of 0.05 or 5%, confidence intervals (CI) were calculated at a confidence level of 95%.

In general, if an observed result was statistically significant at a *P*-value of 0.05, then the null hypothesis did not fall within the 95% CI. Statistical significance is observed when data in one country was different from findings in the others and not due to chance. Significance was analyzed via column proportions and column means tests.

### 2.5 Stimuli

**Figure 1:**
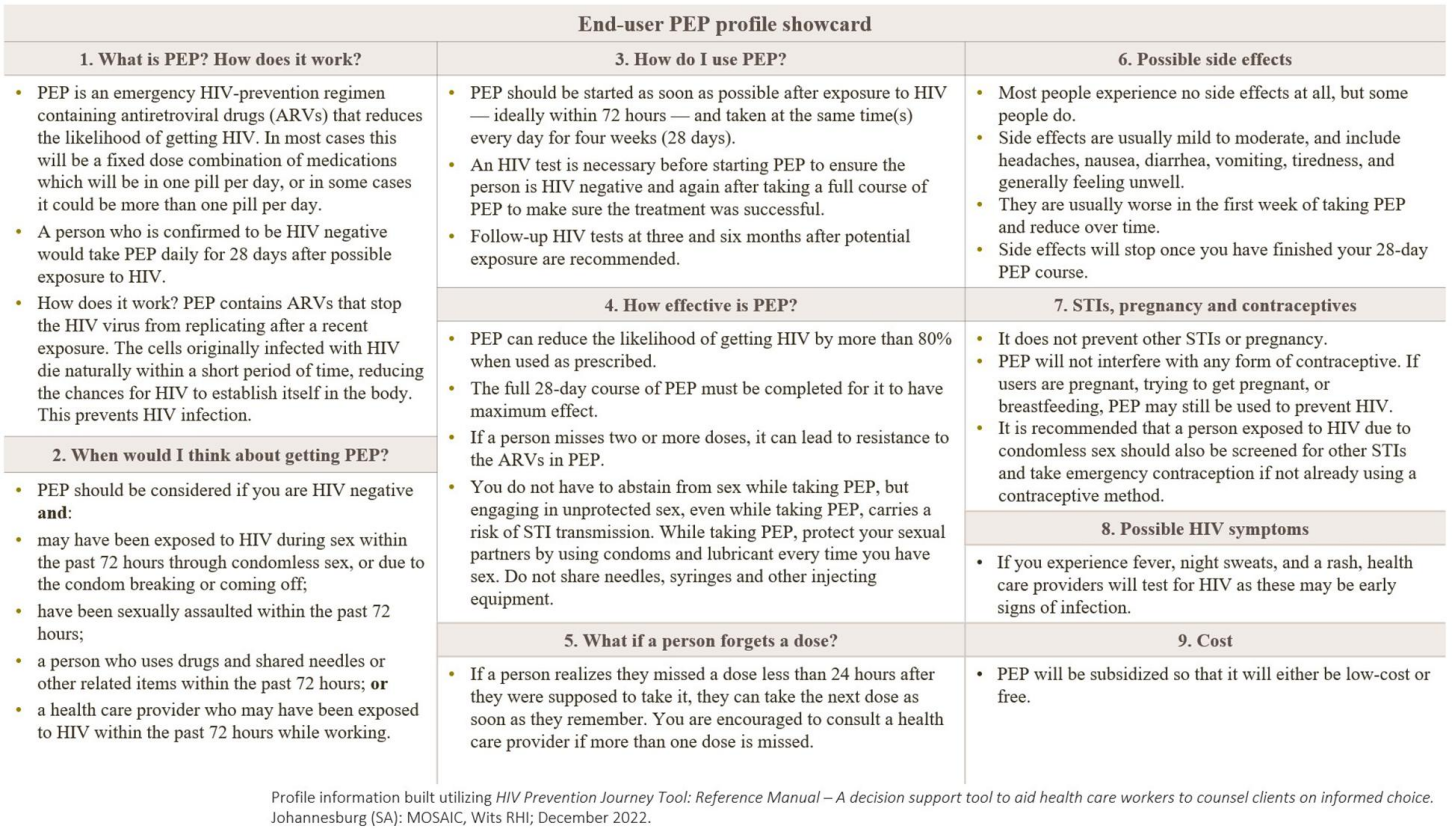
End-user PEP product profile (showcard) presented to survey participants. Information stimulus. The showcard summarizes: what PEP is and how it works (28-day antiretroviral regimen initiated ≤72 hours after exposure); when to consider PEP (sexual, needle-sharing, occupational exposure, or sexual assault among HIV-negative individuals); how to use PEP (daily dosing for 28 days with HIV testing before, at completion, and at 3 and 6 months); effectiveness with adherence; guidance for missed doses; common, usually mild side effects; notes on STIs, pregnancy, and contraceptives; symptoms suggestive of acute HIV infection; and typical cost/subsidy information. After viewing the profile, participants were asked follow-up questions about the content. ARV, antiretroviral; STI, sexually transmitted infection.

**Figure 2:**
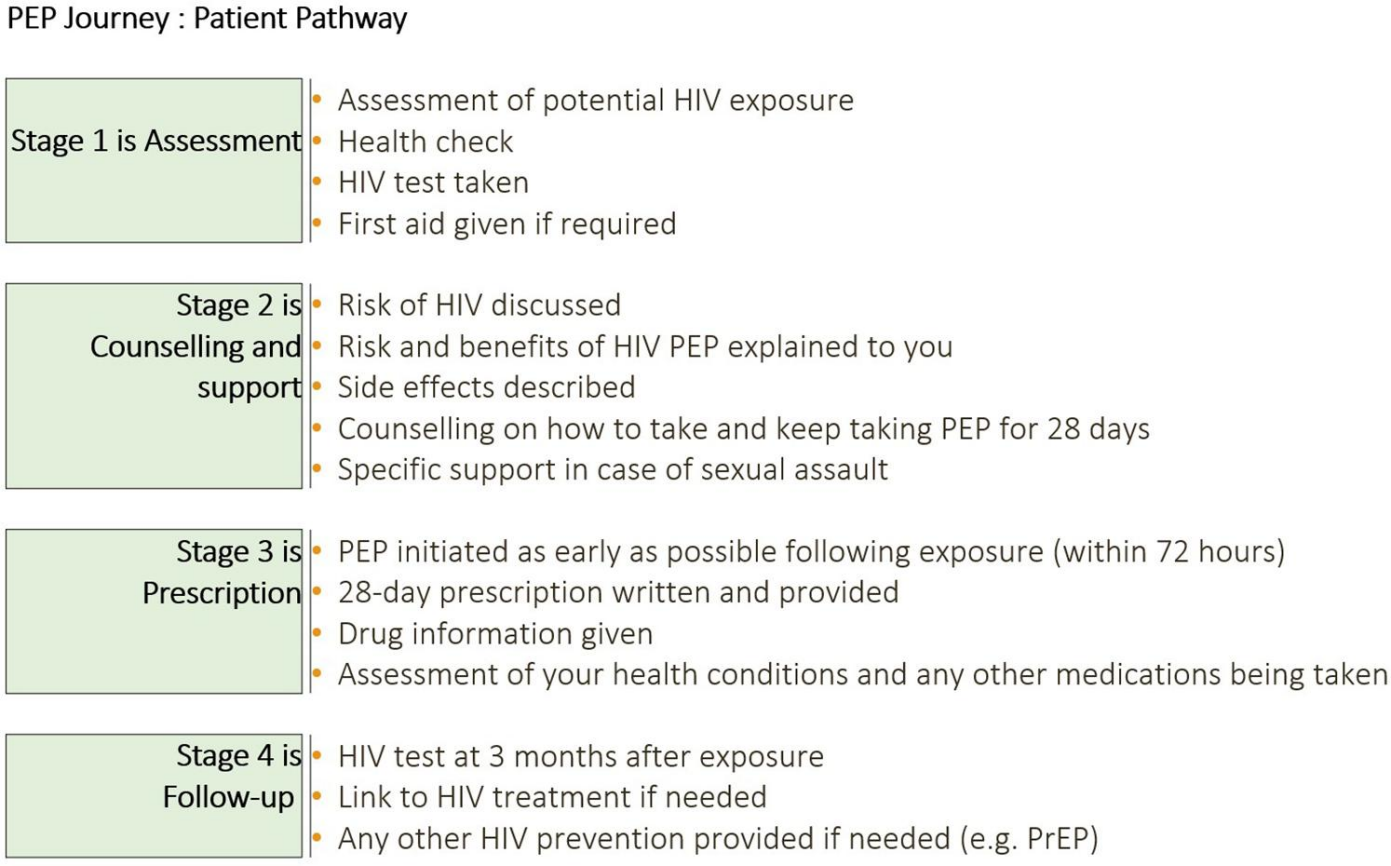
PEP journey—patient pathway showcard presented to survey participants. A visual used to prime respondents on the steps of HIV post-exposure prophylaxis care: Stage 1—Assessment (exposure assessment, health check, HIV test, first aid as needed); Stage 2—Counselling and support (discussion of HIV risk; explanation of PEP risks/benefits and possible side effects; adherence counselling for 28 days; specific support after sexual assault); Stage 3—Prescription (PEP initiation as early as possible and within 72 hours; 28-day prescription; drug information; review of co-morbidities and concomitant medicines); Stage 4—Follow-up (HIV test at 3 months; linkage to HIV treatment if positive; provision of other prevention options, e.g., PrEP). PEP, post-exposure prophylaxis; PrEP, pre-exposure prophylaxis.

End-users were shown stimuli (Figures 1, 2). All materials were translated, and translations were offered throughout the interview to ensure optimal understanding of the research questions and maximize ease of conversation for the respondent.

### 2.6 Translations

All informed consent forms, stimuli and research materials were translated into the main languages spoken in the areas where fieldwork was conducted (Kiswahili in Kenya, Pidgin and Yoruba in Nigeria and Shona and Ndebele in Zimbabwe). Respondents were able to choose languages for written materials and discussion, and to switch if preferred.

### 2.7 Data collection

All interviews were conducted by interviewers from Infotrak (fieldwork partner) who were trained in market research methodologies and were native speakers.

## 3 Results

The results of this research are ordered according to the research objectives outlined in Table 1.

### 3.1 Sources of information on HIV prevention

#### 3.1.1 HIV prevention methods

*Quantitative* Table 5, unprompted recall of known HIV prevention methods, shows that condom use was the most widely known method across all countries (Kenya 86.1%, Nigeria 94.8% and Zimbabwe 98.0%—significantly high) followed by abstinence (61.5%, 45.3% and 88.8% respectively, Zimbabwe significantly high) and not sharing needles [28.8%, 70.7% (Nigeria—significantly high) and 51.3%, respectively].

**Table 5:** Unprompted awareness of methods that can prevent HIV.

USERS		COUNTRY			KENYA					NIGERIA					ZIMBABWE					KENYA		NIGERIA		ZIMBABWE	
	TOTAL	Kenya	Nigeria	Zimbabwe	TOTAL	YW	Men	MSM	FSW	TOTAL	YW	Men	MSM	FSW	TOTAL	YW	Men	MSM	FSW	PEP	NON-PEP	PEP	NON-PEP	PEP	NON-PEP
Base	920	309	307	304	309	101	100	57	51	307	102	104	51	50^d^	304	100	100	52	52	145	164	39^d^	268	120	184
Use condoms	92.9%	86.1%	94.8%	98.0%^b^	**86**.**1%**	86.1%	83.0%	87.7%	90.2%	**94**.**8%**	95.1%	100.0%	84.3%	94.0%	**98**.**0%**	98.0%	97.0%	98.1%	100.0%	88.3%	84.1%	97.4%	94.4%	98.3%	97.8%
Abstinence	65.1%	61.5%	45.3%^a^	88.8%^b^	**61**.**5%**	67.3%	65.0%	56.1%	49.0%	**45**.**3%**	59.8%	51.0%	27.5%	22.0%	**88**.**8%**	88.0%	87.0%	98.1%	84.6%	62.1%	61.0%	41.0%	45.9%	95.0%^c^	84.8%
Do not share needles	50.2%	28.8%^a^	70.7%^b^	51.3%	**28**.**8%**	32.7%	25.0%	31.6%	25.5%	**70**.**7%**	85.3%	83.7%	35.3%	50.0%	**51**.**3%**	50.0%	41.0%	61.5%	63.5%	31.0%	26.8%	71.8%	70.5%	52.5%	50.5%
Get HIV tested	28.7%	34.0%	27.4%	24.7%	**34**.**0%**	15.8%	43.0%	47.4%	37.3%	**27**.**4%**	27.5%	32.7%	21.6%	22.0%	**24**.**7%**	33.0%	29.0%	5.8%	19.2%	38.6%	29.9%	38.5%	25.7%	28.3%	22.3%
Limit the number of sexual partners	27.2%	24.3%	25.7%	31.6%	**24**.**3%**	13.9%	26.0%	40.4%	23.5%	**25**.**7%**	35.3%	37.5%	3.9%	4.0%	**31**.**6%**	37.0%	38.0%	25.0%	15.4%	24.8%	23.8%	38.5%	23.9%	42.5%^c^	24.5%
Use PEP	25.8%	35.3%	4.6%^a^	37.5%	**35**.**3%**	33.7%	24.0%	43.9%	51.0%	**4**.**6%**	5.9%	5.8%	*–*	4.0%	**37**.**5%**	37.0%	32.0%	42.3%	44.2%	55.9%^c^	17.1%	33.3%	0.4%	70.0%^c^	16.3%
Use PrEP	21.8%	28.8%	2.6%^a^	34.2%	**28**.**8%**	20.8%	22.0%	45.6%	39.2%	**2**.**6%**	2.9%	2.9%	3.9%	*–*	**34**.**2%**	29.0%	29.0%	46.2%	42.3%	34.5%^c^	23.8%	10.3%^c^	1.5%	50.0%^c^	23.9%
Do not inject drugs	18.2%	15.5%	16.0%	23.0%^b^	**15**.**5%**	10.9%	15.0%	21.1%	19.6%	**16**.**0%**	12.7%	22.1%	17.6%	8.0%	**23**.**0%**	18.0%	23.0%	36.5%	19.2%	16.6%	14.6%	28.2%^c^	14.2%	25.8%	21.2%
VMMC	11.2%	5.8%	1.0%^a^	27.0%^b^	**5**.**8%**	1.0%	8.0%	12.3%	3.9%	**1**.**0%**	1.0%	*–*	*–*	4.0%	**27**.**0%**	19.0%	41.0%	34.6%	7.7%	6.2%	5.5%	2.6%	0.7%	37.5%^c^	20.1%
Get tested and treated for STDs	10.5%	15.5%	4.6%^a^	11.5%	**15**.**5%**	3.0%	16.0%	33.3%	19.6%	**4**.**6%**	6.9%	4.8%	2.0%	2.0%	**11**.**5%**	13.0%	14.0%	9.6%	5.8%	18.6%	12.8%	7.7%	4.1%	18%^c^	7.1%
I do not know	0.2%	0.6%	*–*	*–*	**0**.**6%**	2.0%	*–*	*–*	*–*	***–***	*–*	*–*	*–*	*–*	***–***	*–*	*–*	*–*	*–*	1.4%	*–*	*–*	*–*	*–*	*–*

HIV, human immunodeficiency virus; PEP, post-exposure prophylaxis; PrEP, pre-exposure prophylaxis; STD, sexually transmitted diseases; VMMC, voluntary medical male circumcision, YW, young women. NB. Key Population includes Female Sex Workers and Men who have Sex with Men.

Bold values highlight the % in the total columns.

^a^ Significantly lower than the other 2 countries.

^b^ Significantly higher than the other 2 countries.

^c^ Statistically higher than PEP/Non-PEP sample.

^d^ low base size.

PrEP (Pre-Exposure Prophylaxis) or PEP use were cited via unprompted recall by 21.8 and 25.8% respectively of end-users overall. In Kenya and Zimbabwe, Female Sex Workers and Men who have Sex with Men demonstrated greater awareness of PrEP and PEP as an HIV prevention method (Kenya, awareness of PrEP: Female Sex Workers 39.2% and Men who have Sex with Men 45.6%; Zimbabwe 42.3% and 46.2% respectively. Kenya, awareness of PEP: Female Sex Workers 51.0% and Men who have Sex with Men 43.9%; Zimbabwe 44.2% and 42.3% respectively).

#### 3.1.2 Awareness of PEP

*Quantitative* Table 6 illustrates that prompted awareness of PEP (i.e., those who did not spontaneously cite PEP as an HIV prevention method as an answer to the previous question) varied across the countries. While overall almost 3 in 5 end-users cited that they had not heard of PEP (56.2%), this was driven by lack of awareness in Nigeria (79.9%) which was significantly high, followed by Zimbabwe (51.6%) and Kenya (26.0%). There was significantly higher awareness of PEP across all end-user types in Kenya (70.2–73.1%), followed by Female Sex Workers and Men who have Sex with Men in Zimbabwe (58.6% and 53.3% respectively). PEP awareness was lowest in Nigeria (15.4%), with the majority of end-user types (Young Women 83.3%, men 82.7%, Female Sex Workers 77.1% and Men who have Sex with Men 70.6%) not aware of PEP. To note, discrepancy on awareness of PEP (unprompted recall 25.8% overall, Table 5 vs. prompted recall 41.1% overall, Table 6) is expected, as prompted awareness typically reveals latent familiarity; while many participants may not think of PEP spontaneously, a proportion do recognize it when prompted.

**Table 6:** Prompted awareness of PEP (asked to users who did not mention PEP previously as a method to prevent HIV).

USERS		COUNTRY			KENYA					NIGERIA					ZIMBABWE					KENYA		NIGERIA		ZIMBABWE	
Has heard of PEP	TOTAL	Kenya	Nigeria	Zimbabwe	TOTAL	YW	Men	MSM	FSW	TOTAL	YW	Men	MSM	FSW	TOTAL	YW	Men	MSM	FSW	PEP	NON-PEP	PEP	NON-PEP	PEP	NON-PEP
Base	683	200	293	190	200	67	76	32^d^	25^d^	293	96	98	51	48^d^	190	63	68	30^d^	29^d^	64	136	26^d^	267	36^d^	154
Yes	**41.1%**	72.0%^b^	15.4%^a^	48.4%	**72.0%**	73.1%	72.4%	65.6%	76.0%	**15**.**4%**	14.6%	15.3%	21.6%	10.4%	**48.4%**	41.3%	48.5%	53.3%	58.6%	98.4%*	59.6%	100.0%*	7.1%	97.2%*	37.0%
No	**56.2%**	26.0%	79.9%^b^	51.6%	**26.0%**	23.9%	26.3%	34.4%	20.0%	**79**.**9%**	83.3%	82.7%	70.6%	77.1%	**51.6%**	58.7%	51.5%	46.7%	41.4%	1.6%	37.5%*	–	87.6%*	2.8%	63.0%*
I do not know	**2.6%**	2.0%	4.8%	–	**2.0%**	3.0%	1.3%	-	4.0%	**4**.**8%**	2.1%	2.0%	7.8%	12.5%	**–**	–	–	–	–	–	2.9%	–	5.2%	–	–

PEP, post-exposure prophylaxis; YW, young women. NB. Key Population includes Female Sex Workers and Men who have Sex with Men.

Bold values highlight the % in the total columns.

^a^ Significantly lower than the other 2 countries.

^b^ Significantly higher than the other 2 countries.

^c^ Statistically higher than PEP/Non-PEP sample.

^d^ low base size.

#### 3.1.3 HIV/PEP: information sources

*Quantitative* Tables 7, 8 display all sources used to obtain information about HIV and PEP across all respondent types. Overall, the greatest number of participants cited doctors as a current source of information on HIV (48.7%, significantly higher in Nigeria) and PEP (44.1%), followed by nurses (40.7% for HIV and 29.3% for PEP), friends (32.9% for HIV and 21.5% for PEP) and the internet (27.6% for HIV and 26.0% for PEP).

**Table 7:** Information sources on HIV (Top 10).

USERS		COUNTRY			KENYA					NIGERIA					ZIMBABWE					KENYA		NIGERIA		ZIMBABWE	
	TOTAL	Kenya	Nigeria	Zimbabwe	TOTAL	YW	Men	MSM	FSW	TOTAL	YW	Men	MSM	FSW	TOTAL	YW	Men	MSM	FSW	PEP	NON-PEP	PEP	NON-PEP	PEP	NON-PEP
Base	920	309	307	304	309	101	100	57	51	307	102	104	51	50	304	100	100	52	52	145	164	39^d^	268	120	184
A doctor	**48**.**7%**	46.6%	67.4%^b^	31.9%^a^	**46**.**6%**	55.4%	60.0%	22.8%	29.4%	**67**.**4%**	76.5%	71.2%	51.0%	58.0%	**31**.**9%**	28.0%	24.0%	50.0%	36.5%	43.4%	49.4%	79.5%	65.7%	34.2%	30.4%
A nurse	**40**.**7%**	26.5%^a^	52.8%^b^	42.8%	**26**.**5%**	38.6%	25.0%	10.5%	23.5%	**52**.**8%**	60.8%	49.0%	43.1%	54.0%	**42**.**8%**	36.0%	32.0%	59.6%	59.6%	24.1%	28.7%	66.7%	50.7%	50.8%^c^	37.5%
Friends	**32**.**9%**	33.0%	21.2%^a^	44.7%^b^	**33**.**0%**	38.6%	26.0%	33.3%	35.3%	**21**.**2%**	19.6%	20.2%	23.5%	24.0%	**44**.**7%**	44.0%	44.0%	34.6%	57.7%	31.7%	34.1%	35.9%^c^	19.0%	50.8%	40.8%
Internet (Google)	**27**.**6%**	29.4%	22.1%^a^	31.2%	**29**.**4%**	23.8%	29.0%	35.1%	35.3%	**22**.**1%**	20.6%	26.9%	27.5%	10.0%	**31**.**2%**	35.0%	35.0%	34.6%	13.5%	26.2%	32.3%	17.9%	22.8%	35.8%	28.3%
Social media (Facebook/Instagram/Twitter)	**24**.**6%**	24.9%	21.2%	27.6%	**24**.**9%**	27.7%	27.0%	17.5%	23.5%	**21**.**2%**	27.5%	26.9%	5.9%	12.0%	**27**.**6%**	32.0%	28.0%	26.9%	19.2%	18.6%	31.2%^c^	17.9%	21.6%	31.7%	25.0%
Health clinic	**23**.**2%**	15.2%	14.3%	40.1%^b^	**15**.**2%**	8.9%	28.0%	8.8%	9.8%	**14**.**3%**	18.6%	18.3%	3.9%	8.0%	**40**.**1%**	37.0%	35.0%	53.8%	42.3%	17.2%	13.4%	25.6%^c^	12.7%	48.3%^c^	34.8%
TV	**20**.**8%**	13.6%^a^	22.1%	26.6%	**13**.**6%**	9.9%	21.0%	12.3%	7.8%	**22**.**1%**	21.6%	31.7%	15.7%	10.0%	**26**.**6%**	30.0%	25.0%	21.2%	28.8%	13.1%	14.0%	15.4%	23.1%	25.8%	27.2%
A community health worker	**20**.**5%**	13.6%^a^	24.8%	23.4%	**13**.**6%**	13.9%	15.0%	10.5%	13.7%	**24**.**8%**	28.4%	26.0%	17.6%	22.0%	**23**.**4%**	24.0%	14.0%	15.4%	48.1%	19.3%^c^	8.5%	25.6%	24.6%	26.7%	21.2%
NGOs/advocacy leaders/outreach organisations	**19**.**6%**	14.6%	19.2%	25.0%	**14**.**6%**	14.9%	16.0%	15.8%	9.8%	**19**.**2%**	11.8%	18.3%	33.3%	22.0%	**25**.**0%**	21.0%	25.0%	44.2%	13.5%	17.2%	12.2%	25.6%	18.3%	30.8%	21.2%
A pharmacist	**19**.**5%**	13.6%	27.7%^b^	17.1%	**13**.**6%**	7.9%	24.0%	8.8%	9.8%	**27**.**7%**	25.5%	26.0%	37.3%	26.0%	**17**.**1%**	10.0%	15.0%	32.7%	19.2%	16.6%	11.0%	30.8%	27.2%	20.0%	15.2%

HIV, human immunodeficiency disease, NGO, non-governmental organization, PEP, post-exposure prophylaxis; TV, television, YW, young women. NB. Key Population includes Female Sex Workers and Men who have Sex with Men.

Bold values highlight the % in the total columns.

^a^ Significantly lower than the other 2 countries.

^b^ Significantly higher than the other 2 countries.

^c^ Statistically higher than PEP/Non-PEP sample.

^d^ low base size.

**Table 8:** Information sources on PEP (Top 10).

USERS		COUNTRY			KENYA					NIGERIA					ZIMBABWE					KENYA		NIGERIA		ZIMBABWE	
	TOTAL	Kenya	Nigeria	Zimbabwe	TOTAL	YW	Men	MSM	FSW	TOTAL	YW	Men	MSM	FSW	TOTAL	YW	Men	MSM	FSW	PEP	NON-PEP	PEP	NON-PEP	PEP	NON-PEP
Base	920	309	307	304	309	101	100	57	51	307	102	104	51	50^d^	304	100	100	52	52	145	164	39^d^	268	120	184
A doctor	**44**.**1%**	47.6%	55.0%	29.6%^a^	**47**.**6%**	51.5%	56.0%	31.6%	41.2%	**55**.**0%**	52.9%	54.8%	60.8%	54.0%	**29**.**6%**	25.0%	24.0%	50.0%	28.8%	37.2%	56.7%^c^	66.7%	53.4%	13.3%	40.2%^c^
A nurse	**29**.**3%**	15.9%^a^	35.2%	37.2%	**15**.**9%**	26.7%	9.0%	3.5%	21.6%	**35**.**2%**	31.4%	36.5%	39.2%	36.0%	**37**.**2%**	36.0%	27.0%	36.5%	59.6%	10.3%	20.7%^c^	43.6%	34.0%	28.3%	42.9%^c^
Internet (Google)	**26**.**0%**	24.3%	25.7%	28.0%	**24**.**3%**	21.8%	19.0%	35.1%	27.5%	**25**.**7%**	22.5%	33.7%	31.4%	10.0%	**28**.**0%**	35.0%	25.0%	36.5%	11.5%	17.2%	30.5%^c^	5.1%	28.7%^c^	26.7%	28.8%
Social media (Facebook/Instagram/Twitter)	**21**.**7%**	19.7%	25.4%	20.1%	**19**.**7%**	23.8%	17.0%	17.5%	19.6%	**25**.**4%**	27.5%	33.7%	13.7%	16.0%	**20**.**1%**	26.0%	17.0%	21.2%	13.5%	13.1%	25.6%^c^	7.7%	28.0%^c^	17.5%	21.7%
Friends	**21**.**5%**	26.2%	7.8%^a^	30.6%	**26**.**2%**	36.6%	18.0%	17.5%	31.4%	**7**.**8%**	6.9%	6.7%	9.8%	10.0%	**30**.**6%**	40.0%	33.0%	9.6%	28.8%	30.3%	22.6%	15.4%	6.7%	51.7%^c^	16.8%
Health clinic	**21**.**5%**	13.6%	12.1%	39.1%^b^	**13**.**6%**	11.9%	23.0%	-	13.7%	**12**.**1%**	12.7%	14.4%	13.7%	4.0%	**39**.**1%**	41.0%	30.0%	50.0%	42.3%	8.3%	18.3%^c^	7.7%	12.7%	35.0%	41.8%
NGOs/advocacy leaders/outreach organisations	**18**.**3%**	10.7%^a^	21.2%	23.0%	**10**.**7%**	14.9%	6.0%	15.8%	5.9%	**21**.**2%**	9.8%	11.5%	51.0%	34.0%	**23**.**0%**	19.0%	24.0%	38.5%	13.5%	10.3%	11.0%	7.7%	23.1%^c^	25.0%	21.7%
A pharmacist	**16**.**1%**	10.7%^a^	19.5%	18.1%	**10**.**7%**	11.9%	12.0%	7.0%	9.8%	**19**.**5%**	15.7%	20.2%	25.5%	20.0%	**18**.**1%**	15.0%	12.0%	30.8%	23.1%	7.6%	13.4%	12.8%	20.5%	12.5%	21.7%^c^
TV	**15**.**8%**	9.4%^a^	20.5%	17.4%	**9**.**4%**	10.9%	12.0%	5.3%	5.9%	**20**.**5%**	24.5%	24.0%	11.8%	14.0%	**17**.**4%**	24.0%	15.0%	13.5%	13.5%	6.2%	12.2%	5.1%	22.8%^c^	13.3%	20.1%
A community health worker	**15**.**4%**	10%^a^	15.6%	20.7%	**10**.**0%**	10.9%	8.0%	5.3%	17.6%	**15**.**6%**	14.7%	13.5%	21.6%	16.0%	**20**.**7%**	20.0%	14.0%	1.9%	53.8%	11.0%	9.1%	15.4%	15.7%	23.3%	19.0%

NGO, non-governmental organization, PEP, post-exposure prophylaxis; TV, television, YW, young women. NB. Key Population includes Female Sex Workers and Men who have Sex with Men.

Bold values highlight the % in the total columns.

^a^ Significantly lower than the other 2 countries.

^b^ Significantly higher than the other 2 countries.

^c^ Statistically higher than PEP/Non-PEP sample.

^d^ Low base size.

### 3.2 Reactions to PEP

#### 3.2.1 PEP profile evaluation

*Quantitative* End-users were shown PEP information in the form of a profile (Figure 1). The top four statements selected as positives across all respondent types are shown in Table 9. The two most selected statements illustrate how messages around PEP as an emergency HIV prevention option were considered positive.

**Table 9:** PEP profile evaluation (Top 10 positives).

COUNTRY					KENYA					NIGERIA					ZIMBABWE					KENYA		NIGERIA		ZIMBABWE	
USERS	TOTAL	Kenya	Nigeria	Zimbabwe	TOTAL	YW	Men	MSM	FSW	TOTAL	YW	Men	MSM	FSW	TOTAL	YW	Men	MSM	FSW	PEP	NON-PEP	PEP	NON-PEP	PEP	NON-PEP
Base	920	309	307	304	309	101	100	57	51	307	102	104	51	50^d^	304	100	100	52	52	145	164	39^d^	268	120	184
PEP is an emergency HIV-prevention regimen containing ARVs that reduces the likelihood of getting HIV.	**66**.**3%**	60.5%	82.1%^b^	56.2%	**60**.**5%**	66.3%	50.0%	63.2%	66.7%	**82**.**1%**	89.2%	85.6%	66.7%	76.0%	**56**.**2%**	53.0%	50.0%	67.3%	63.5%	65.5%	56.1%	87.2%	81.3%	45.8%	63.0%
PEP contains ARVs that stop the HIV virus from replicating after a recent exposure.This prevents HIV.	**52**.**3%**	48.5%	48.5%	59.9%^b^	**48**.**5%**	59.4%	34.0%	57.9%	45.1%	**48**.**5%**	47.1%	41.3%	54.9%	60.0%	**59**.**9%**	47.0%	50.0%	84.6%	78.8%	49.7%	47.6%	53.8%	47.8%	55.0%	63.0%
A person who is confirmed to be HIV negative would take PEP daily for 28 days after possible exposure to HIV.	**43**.**6%**	42.7%	49.2%	38.8%	**42**.**7%**	52.5%	38.0%	45.6%	29.4%	**49**.**2%**	47.1%	51.9%	37.3%	60.0%	**38**.**8%**	41.0%	40.0%	44.2%	26.9%	48.3%	37.8%	71.8%^c^	45.9%	35.0%	41.3%
PEP should be considered..72 h through condomless sex, or due to the condom breaking or coming off.	**34**.**7%**	32.0%	29.3%	42.8%^b^	**32**.**0%**	40.6%	22.0%	35.1%	31.4%	**29**.**3%**	36.3%	26.0%	27.5%	24.0%	**42**.**8%**	34.0%	42.0%	48.1%	55.8%	29.0%	34.8%	33.3%	28.7%	35.8%	47.3%^c^
PEP can reduce the likelihood of getting HIV by more than 80% when used as prescribed.	**34**.**6%**	16.8%^a^	34.2%	53.0%^b^	**16**.**8%**	17.8%	22.0%	8.8%	13.7%	**34**.**2%**	37.3%	44.2%	27.5%	14.0%	**53**.**0%**	45.0%	43.0%	69.2%	71.2%	13.8%	19.5%	35.9%	34.0%	54.2%	52.2%
PEP should be started as soon as possible after exposure to HIV—ideally within 72 h.	**29**.**0%**	27.8%	29.6%	29.6%	**27**.**8%**	35.6%	30.0%	17.5%	19.6%	**29**.**6%**	40.2%	36.5%	13.7%	10.0%	**29**.**6%**	34.0%	28.0%	30.8%	23.1%	23.4%	31.7%	35.9%	28.7%	29.2%	29.9%
PEP should be considered..72 h having been sexually assaulted.	**28**.**4%**	20.1%	25.4%	39.8%^b^	**20**.**1%**	28.7%	14.0%	14.0%	21.6%	**25**.**4%**	30.4%	24.0%	19.6%	24.0%	**39**.**8%**	40.0%	33.0%	50.0%	42.3%	16.6%	23.2%	28.2%	25.0%	39.2%	40.2%
In most cases this will be a fixed dose combination of medications which will be in one pill per day..	**27**.**9%**	22.7%	39.4%^b^	21.7%	**22**.**7%**	30.7%	17.0%	29.8%	9.8%	**39**.**4%**	24.5%	31.7%	54.9%	70.0%	**21**.**7%**	19.0%	23.0%	30.8%	15.4%	22.8%	22.6%	53.8%^c^	37.3%	22.5%	21.2%
The full 28-day course of PEP must be completed for it to have a maximum effect.	**22**.**0%**	12.9%^a^	23.1%	29.9%	**12**.**9%**	17.8%	16.0%	7.0%	3.9%	**23**.**1%**	23.5%	26.0%	31.4%	8.0%	**29**.**9%**	26.0%	24.0%	44.2%	34.6%	9.7%	15.9%	30.8%	22.0%	40.8%^c^	22.8%
PEP should be considered..72 h as a person who uses drugs and shared needles or other related items.	**21**.**7%**	18.8%	19.2%	27.3%^b^	**18**.**8%**	27.7%	12.0%	15.8%	17.6%	**19**.**2%**	26.5%	18.3%	13.7%	12.0%	**27**.**3%**	23.0%	30.0%	36.5%	21.2%	17.2%	20.1%	20.5%	19.0%	32.5%	23.9%

ARVs, anti-retrovirals; HIV, human immunodeficiency Disease; PEP, post-exposure prophylaxis; YW, young women. NB. Key Population includes Female Sex Workers and Men who have Sex with Men.

Bold values highlight the % in the total columns.

^a^ Significantly lower than the other 2 countries.

^b^ Significantly higher than the other 2 countries.

^c^ Statistically higher than PEP/Non-PEP sample.

^d^ low base size.

*Qualitative* The scenarios outlined in the profile which would prompt seeking PEP that resonated most for end-users were those considered emergency situations, including condomless sex or condom malfunction, sexual assault/rape, not knowing the status of a (new) sexual partner, needle sharing or accidental cuts/exposure to blood, and financial pressures (accepting more money for condomless sex, e.g., when monthly rent is due). Sex workers explained during IDIs how financial pressures can result in taking risks:

“PEP is a very great tool in my life. As a sex worker, these HIV prevention methods are not 100% efficient. Usually, I use condoms, and sometimes they break, exposing us. Some clients will come and pay good money and say I don't want a condom; I need money, so I sleep with such a man without protection.” Female Sex Worker

The PEP profile information (Figure 1) was described as educational, clear and helpful; many respondents asked to keep the profile, and/or stated that it should be shared widely within the community.

“Ok, I like that it has been explained—how the PEP actually works in the body and how it prevents it because I only knew that it prevents but not how it does that. Because I think it’s explaining every detail.” Transgender or Gender Diverse respondent

Those who had taken PEP before had mixed views as to whether they were given the same information as in the profile:

“I didn’t get all this information when I got PEP. It was a friend of mine, so the friend of mine just told me that I need to use it daily for 28 days, that I should swallow this thing that it will help to prevent HIV basically.” Female Sex Worker

“So, this is more information than the hospital. In the hospital, they tell you to take it for 28 days, and you go sort yourself out thereafter. Seriously, I wasn't told anything else; I was just instructed to go and get tested, then come back and get the medication and go home.” Female Sex Worker

#### 3.2.2 Perceived likelihood to use PEP

*Quantitative* Table 10 shows the perceived likelihood to use across all respondent types. The vast majority of end-users reported likelihood to use PEP if exposed to HIV (86.4% overall), after reading the PEP profile (Figure 1) which included a list of scenarios defining exposure.

**Table 10:** Perceived likelihood to use PEP if exposed to HIV.

USERS		COUNTRY			KENYA					NIGERIA					ZIMBABWE					KENYA		NIGERIA		ZIMBABWE	
	TOTAL	Kenya	Nigeria	Zimbabwe	TOTAL	YW	Men	MSM	FSW	TOTAL	YW	Men	MSM	FSW	TOTAL	YW	Men	MSM	FSW	PEP	NON-PEP	PEP	NON-PEP	PEP	NON-PEP
Base	920	309	307	304	309	101	100	57	51	307	102	104	51	50^d^	304	100	100	52	52	145	164	39^d^	268	120	184
Yes, I would definitely/probably use it again	**86**.**4%**	85.8%	96.4%^b^	77%^a^	**85**.**8%**	92.1%	84.0%	78.9%	84.3%	**96**.**4%**	97.1%	94.2%	98.0%	98.0%	**77**.**0%**	73.0%	77.0%	82.7%	78.8%	89.0%	82.9%	82.1%	98.5%^c^	60.8%	87.5%
*Yes, I would definitely use it again*	**70**.**1%**	68.6%	87.0%^b^	54.6%^a^	**68**.**6%**	77.2%	63.0%	64.9%	66.7%	**87**.**0%**	90.2%	90.4%	80.4%	80.0%	**54**.**6%**	47.0%	53.0%	67.3%	59.6%	72.4%	65.2%	74.4%	88.8%^c^	39.2%	64.7%
*I would probably use it again*	**16**.**3%**	17.2%	9.4%^a^	22.4%	**17**.**2%**	14.9%	21.0%	14.0%	17.6%	**9**.**4%**	6.9%	3.8%	17.6%	18.0%	**22**.**4%**	26.0%	24.0%	15.4%	19.2%	16.6%	17.7%	7.7%	9.7%	21.7%	22.8%
I am not sure whether I would use it again	**8**.**5%**	10.4%	2.0%^a^	13.2%	**10**.**4%**	7.9%	9.0%	15.8%	11.8%	**2**.**0%**	2.0%	3.8%	-	-	**13**.**2%**	17.0%	10.0%	11.5%	13.5%	8.3%	12.2%	12.8%^c^	0.4%	16.7%	10.9%
No, I would definitely/probably not use it again	**5**.**1%**	3.9%	1.6%	9.9%^b^	**3**.**9%**	-	7.0%	5.3%	3.9%	**1**.**6%**	1.0%	1.9%	2.0%	2.0%	**9**.**9%**	10.0%	13.0%	5.8%	7.7%	2.8%	4.9%	5.1%	1.1%	22.5%^c^	1.6%
*I would probably not use it again*	**3**.**4%**	2.3%	1.0%	6.9%^b^	**2**.**3%**	-	3.0%	5.3%	2.0%	**1**.**0%**	1.0%	-	2.0%	2.0%	**6**.**9%**	6.0%	8.0%	5.8%	7.7%	2.1%	2.4%	2.6%	0.7%	15.8%^c^	1.1%
*No, I definitely would not use it again*	**1**.**7%**	1.6%	0.7%	3.0%	**1**.**6%**	-	4.0%	-	2.0%	**0**.**7%**	-	1.9%	-	-	**3**.**0%**	4.0%	5.0%	-	-	0.7%	2.4%	2.6%	0.4%	6.7%^c^	0.5%

HIV, human immunodeficiency disease, PEP, post-exposure prophylaxis; YW, young women. NB. Key Population includes Female Sex Workers and Men who have Sex with Men.

Bold values highlight the % in the total columns.

^a^ Significantly lower than the other 2 countries.

^b^ Significantly higher than the other 2 countries.

^c^ Statistically higher than PEP/Non-PEP sample.

^d^ Low base size.

## 4 Preferred access points for PEP

*Quantitative* Table 11 shows perceived preference for places where PEP is or could be provided. Most respondents selected, from a pre-defined list, a general hospital as the place where they would prefer to access PEP (81.2% overall). Most respondents from Zimbabwe found all options acceptable (at least 58.6%), with a significantly higher number selecting community-based and outreach services. In Kenya and Zimbabwe, sexual health and HIV clinics were selected more often by Men who have Sex with Men than general hospitals, with the converse being the case in Nigeria.

**Table 11:** Locations respondents are willing to access PEP from (Top 10).

USERS		COUNTRY			KENYA					NIGERIA					ZIMBABWE					KENYA		NIGERIA		ZIMBABWE	
	TOTAL	Kenya	Nigeria	Zimbabwe	TOTAL	YW	Men	MSM	FSW	TOTAL	YW	Men	MSM	FSW	TOTAL	YW	Men	MSM	FSW	PEP	NON-PEP	PEP	NON-PEP	PEP	NON-PEP
Base	920	309	307	304	309	101	100	57	51	307	102	104	51	50^d^	304	100	100	52	52	145	164	39^d^	268	120	184
General hospital	**81**.**2%**	73.1%^a^	86.6%	83.9%	**73**.**1%**	77.2%	74.0%	63.2%	74.5%	**86**.**6%**	82.4%	87.5%	92.2%	88.0%	**83**.**9%**	93.0%	92.0%	48.1%	86.5%	72.4%	73.8%	92.3%	85.8%	86.7%	82.1%
A clinic	**74**.**8%**	63.4%^a^	74.9%	86.2%^b^	**63**.**4%**	65.3%	67.0%	56.1%	60.8%	**74**.**9%**	70.6%	80.8%	82.4%	64.0%	**86**.**2%**	92.0%	87.0%	75.0%	84.6%	67.6%	59.8%	69.2%	75.7%	95.0%^c^	80.4%
An independent pharmacy	**65**.**3%**	39.2%^a^	72.3%	84.9%^b^	**39**.**2%**	30.7%	50.0%	40.4%	33.3%	**72**.**3%**	62.7%	66.3%	92.2%	84.0%	**84**.**9%**	81.0%	85.0%	94.2%	82.7%	42.8%	36.0%	53.8%	75.0%^c^	91.7%^c^	80.4%
A pharmacy at a hospital	**64**.**3%**	46.6%^a^	69.7%	77.0%^b^	**46**.**6%**	42.6%	49.0%	49.1%	47.1%	**69**.**7%**	69.6%	66.3%	76.5%	70.0%	**77**.**0%**	85.0%	85.0%	50.0%	73.1%	48.3%	45.1%	66.7%	70.1%	85.0%^c^	71.7%
An HIV clinic	**63**.**9%**	70.2%	46.6%^a^	75.0%	**70**.**2%**	62.4%	72.0%	77.2%	74.5%	**46**.**6%**	46.1%	42.3%	54.9%	48.0%	**75**.**0%**	75.0%	76.0%	57.7%	90.4%	75.9%^c^	65.2%	59.0%	44.8%	80.8%	71.2%
A sexual health clinic	**63**.**7%**	70.9%	45.0%^a^	75.3%	**70**.**9%**	60.4%	74.0%	82.5%	72.5%	**45**.**0%**	40.2%	41.3%	56.9%	50.0%	**75**.**3%**	68.0%	78.0%	67.3%	92.3%	75.9%	66.5%	48.7%	44.4%	82.5%^c^	71.2%
Community-based clinic	**57**.**6%**	39.5%^a^	57.7%	76.0%^b^	**39**.**5%**	30.7%	41.0%	36.8%	56.9%	**57**.**7%**	57.8%	50.0%	70.6%	60.0%	**76**.**0%**	83.0%	76.0%	46.2%	92.3%	42.8%	36.6%	43.6%	59.7%	82.5%	71.7%
A pharmacy next to a clinic	**54**.**3%**	38.8%^a^	48.2%	76.3%^b^	**38**.**8%**	32.7%	44.0%	40.4%	39.2%	**48**.**2%**	44.1%	45.2%	58.8%	52.0%	**76**.**3%**	79.0%	81.0%	59.6%	78.8%	43.4%	34.8%	38.5%	49.6%	85.8%^c^	70.1%
Mobile clinic	**39**.**7%**	33.3%	26.4%	59.5%^b^	**33**.**3%**	19.8%	50.0%	36.8%	23.5%	**26**.**4%**	25.5%	20.2%	39.2%	28.0%	**59**.**5%**	60.0%	65.0%	50.0%	57.7%	32.4%	34.1%	25.6%	26.5%	73.3%^c^	50.5%
Outreach organisation	**39**.**5%**	29.4%	30.6%	58.6%^b^	**29**.**4%**	19.8%	43.0%	28.1%	23.5%	**30**.**6%**	14.7%	16.3%	74.5%	48.0%	**58**.**6%**	48.0%	65.0%	65.4%	59.6%	29.0%	29.9%	23.1%	31.7%	69.2%^c^	51.6%

HIV, human immunodeficiency disease, PEP, post-exposure prophylaxis; YW, young women. NB. Key Population includes Female Sex Workers and Men who have Sex with Men.

Bold values highlight the % in the total columns.

^a^ Significantly lower than the other 2 countries.

^b^ Significantly higher than the other 2 countries.

^c^ Statistically higher than PEP/Non-PEP sample.

^d^ Low base size.

*Convenience* was cited as the top reason (with moderators using a pre-defined list to code participants' verbal responses) in all three countries for choice of location (68.2% overall) (Table 12). This was followed by *trustworthiness* (82.4% in Nigeria, significantly high) or *trust in upholding confidentiality* (59.2% in Zimbabwe). Furthermore, the need for rapid access was reported by end-users in Nigeria—*close to my home* (73.3%) – and Zimbabwe: *quick access to PEP* (63.5%). While convenience reasons were frequently cited, key populations in Nigeria and Zimbabwe tended to value *trust regarding confidentiality* (mean values 60.4% and 67.3%) and *discretion* (mean values 51.5% and 60.6% respectively) most highly. Lastly, end-users cited *knowledgeable staff* as an important reason in choosing a location to access PEP (55.8% overall; 43.4% in Kenya, 75.2% in Nigeria and 48.7% in Zimbabwe).

**Table 12:** Reasons for choice of location to access PEP (Top 10).

USERS		COUNTRY			KENYA					NIGERIA					ZIMBABWE					KENYA		NIGERIA		ZIMBABWE	
	TOTAL	Kenya	Nigeria	Zimbabwe	TOTAL	YW	Men	MSM	FSW	TOTAL	YW	Men	MSM	FSW	TOTAL	YW	Men	MSM	FSW	PEP	NON-PEP	PEP	NON-PEP	PEP	NON-PEP
Base	920	309	307	304	309	101	100	57	51	307	102	104	51	50^d^	304	100	100	52	52	145	164	39^d^	268	120	184
Convenience	**68**.**2%**	54.4%^a^	83.4%^b^	66.8%	**54**.**4%**	64.4%	49.0%	57.9%	41.2%	**83**.**4%**	86.3%	79.8%	92.2%	76.0%	**66**.**8%**	64.0%	65.0%	75.0%	67.3%	27.6%	78.0%^c^	38.5%	89.9%^c^	34.2%	88.0%^c^
Trustworthiness	**56**.**5%**	44.7%	82.4%^b^	42.4%	**44**.**7%**	58.4%	36.0%	36.8%	43.1%	**82**.**4%**	82.4%	78.8%	88.2%	84.0%	**42**.**4%**	44.0%	42.0%	40.4%	42.3%	17.9%	68.3%^c^	35.9%	89.2%^c^	13.3%	61.4%^c^
Knowledgeable	**55**.**8%**	43.4%	75.2%^b^	48.7%	**43**.**4%**	47.5%	45.0%	40.4%	35.3%	**75**.**2%**	74.5%	73.1%	82.4%	74.0%	**48**.**7%**	51.0%	48.0%	42.3%	51.9%	-	81.7%^c^	-	86.2%^c^	-	80.4%^c^
Trusted regarding confidentiality/confidential	**54**.**6%**	46.9%^a^	57.7%	59.2%	**46**.**9%**	61.4%	36.0%	43.9%	43.1%	**57**.**7%**	60.8%	51.9%	66.7%	54.0%	**59**.**2%**	55.0%	55.0%	82.7%	51.9%	29.0%	62.8%^c^	15.4%	63.8%^c^	24.2%	82.1%^c^
Close to my home/Closest place to where I live/convenient location	**53**.**6%**	27.5%^a^	73.3%^b^	60.2%	**27**.**5%**	24.8%	29.0%	21.1%	37.3%	**73**.**3%**	74.5%	70.2%	72.5%	78.0%	**60**.**2%**	62.0%	53.0%	51.9%	78.8%	26.2%	28.7%	23.1%	80.6%^c^	30.0%	79.9%^c^
Speed/quick access to PEP	**53**.**2%**	37.9%^a^	58.3%	63.5%	**37**.**9%**	52.5%	28.0%	40.4%	25.5%	**58**.**3%**	72.5%	66.3%	37.3%	34.0%	**63**.**5%**	54.0%	63.0%	80.8%	65.4%	11.0%	61.6%^c^	23.1%	63.4%^c^	24.2%	89.1%^c^
A place I already know/trust, A place I already know	**47**.**6%**	21.4%^a^	68.4%^b^	53.3%	**21**.**4%**	28.7%	13.0%	17.5%	27.5%	**68**.**4%**	71.6%	68.3%	68.6%	62.0%	**53**.**3%**	48.0%	52.0%	57.7%	61.5%	18.6%	23.8%	38.5%	72.8%^c^	27.5%	70.1%^c^
Price/more affordable	**42**.**3%**	24.6%^a^	53.1%	49.3%	**24**.**6%**	38.6%	15.0%	17.5%	23.5%	**53**.**1%**	49.0%	49.0%	62.7%	60.0%	**49**.**3%**	49.0%	45.0%	36.5%	71.2%	6.9%	40.2%^c^	12.8%	59.0%^c^	25.8%	64.7%^c^
Discreet/people don't know I'm accessing it	**40**.**5%**	27.8%	43.0%^a^	51.0%^b^	**27**.**8%**	27.7%	24.0%	36.8%	25.5%	**43**.**0%**	42.2%	35.6%	56.9%	46.0%	**51**.**0%**	47.0%	45.0%	69.2%	51.9%	4.8%	48.2%^c^	12.8%	47.4%^c^	18.3%	72.3%^c^
There would not be judgement/bad treatment/stigma	**39**.**3%**	23.9%^a^	45.6%	48.7%	**23**.**9%**	29.7%	18.0%	17.5%	31.4%	**45**.**6%**	46.1%	50.0%	41.2%	40.0%	**48**.**7%**	44.0%	43.0%	71.2%	46.2%	5.5%	40.2%^c^	20.5%	49.3%^c^	20.0%	67.4%^c^

PEP, post-exposure prophylaxis; YW, young women. NB. Key Population includes Female Sex Workers and Men who have Sex with Men.

Bold values highlight the % in the total columns.

^a^ Significantly lower than the other 2 countries.

^b^ Significantly higher than the other 2 countries.

^c^ Statistically higher than PEP/Non-PEP sample.

^d^ Low base size.

## 5 PEP pathway

### 5.1 Recall of PEP pathway among end-users with PEP experience

*Quantitative* Among PEP-experienced end-users (*n* = 304), there was high recall of the steps in the WHO PEP pathway having been carried out or available when they accessed PEP (Figure 2). Excluding some steps that would be case-specific (such as provision of first aid, specific support in case of sexual assault, and link to HIV treatment if needed), recall scores ranged from 81.6–98.4% overall (Supplementary Table S4).

### 5.2 Perceived ease/difficulty of each step of PEP pathway

End-users also perceived all steps of the PEP pathway to be very easy or easy, reporting overall scores of 77.2–92.0% (Supplementary Table S1).

*Qualitative* Nearly all end-users described the counseling and support stage of the WHO PEP pathway (Figure 2) as the most important. End-users described a set of information and support points which they felt would assist PEP treatment initiation and completion, which included: empathetic assessment, clear and correct advice at prescription, checking in to support with side effects within the first and second weeks, and reminders for follow-up HIV tests at 3 and 6 months. Prospects of PEP completion were considered compromised in the absence of correct advice and support:

“I think counseling and support are the most important aspects because when seeking PEP, individuals are often traumatized and uncertain about the consequences of their actions, so l think having adequate proper counseling and support is crucial during such a vulnerable time.” MSM

“I consider counseling essential in the whole experience because it was counseling that made me resilient in taking the PEP pills. My health advisor encouraged me to keep on taking the PEP pills, so I would say counseling is critical in the experience of side effects as you take PEP.” Young Woman

**Figure 3:**
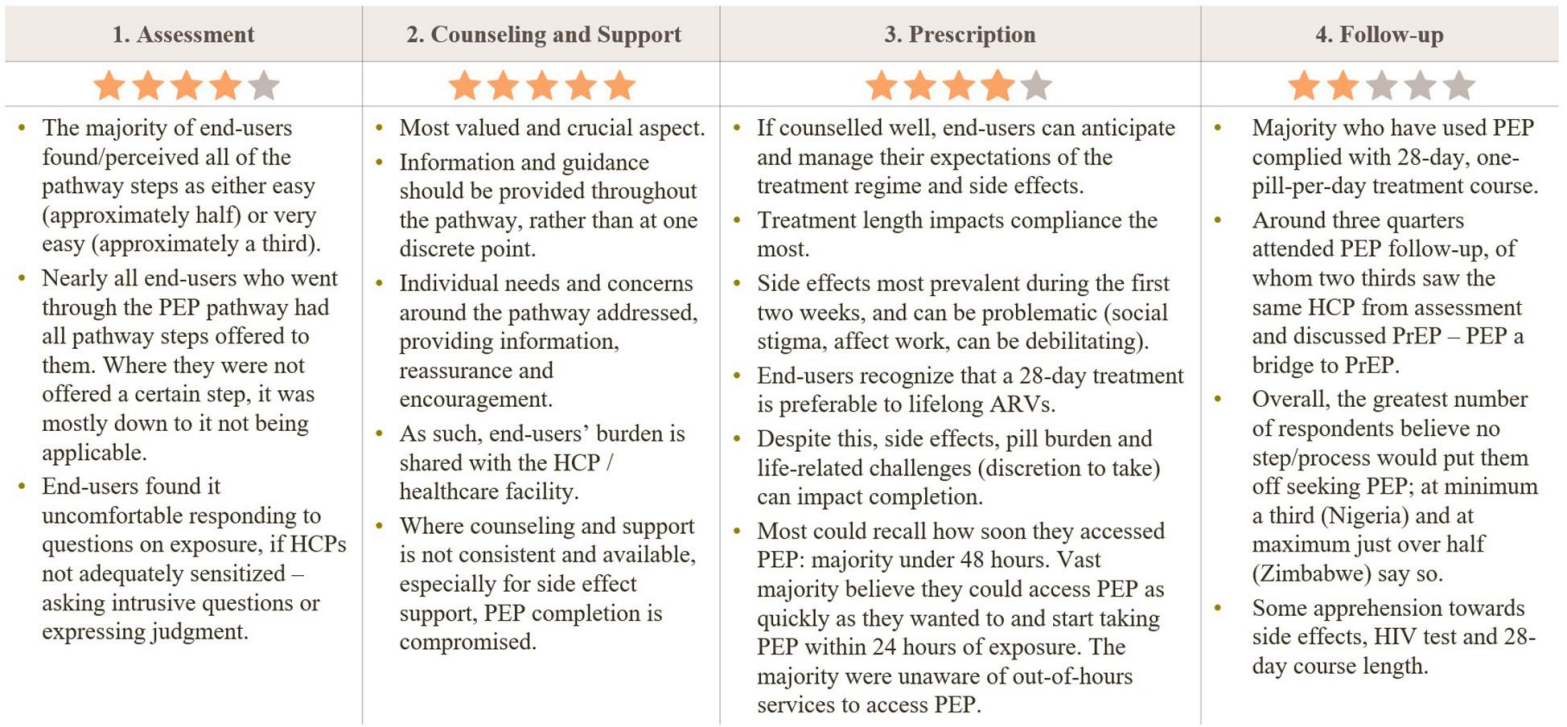
Participant perceptions of the PEP care pathway by stage. Star ratings summarize how positively end-users viewed each stage (1 = lowest, 5 = highest): Assessment ★★★★☆; Counseling & Support ★★★★★; Prescription ★★★★☆; Follow-up ★★★☆☆. Key themes: most steps were perceived as easy/very easy and usually offered when applicable; some discomfort answering exposure questions if health-care providers (HCPs) were not sensitized. Counseling/support was the most valued element-information should be continuous, tailored to needs, and available for side-effect management; when inconsistent, completion suffers. During prescription, good counseling helps set expectations; 28-day duration most affects adherence; early side effects (first two weeks) and pill burden can hinder completion; many accessed PEP within 48 hours but were often unaware of out-of-hours services. For follow-up, most completed the 28-day course and ∼¾ attended follow-up, ∼⅔ with the same HCP; PEP often served as a bridge to PrEP. Overall, many reported that no step would deter them, though some expressed apprehension about side effects, HIV testing, and the 28-day course. HCP, health-care provider; ARV, antiretroviral; PrEP, pre-exposure prophylaxis.

Figure 3 PEP pathway support points.

## 6 Broadening access

*Quantitative* Clinical routes (general hospital, clinic, pharmacy) were perceived as the most important means of broadening access to PEP (60+% overall) (Supplementary Table S2). Outreach and community-based services were perceived by many as potentially acceptable access points (39.5%, 57.6%). Non-clinical and alternative point-of-sale (POS) technologies (such as vending machines and websites) were least preferred by end-users (<30%), along with police stations (9.1%).

**Figure 4:**
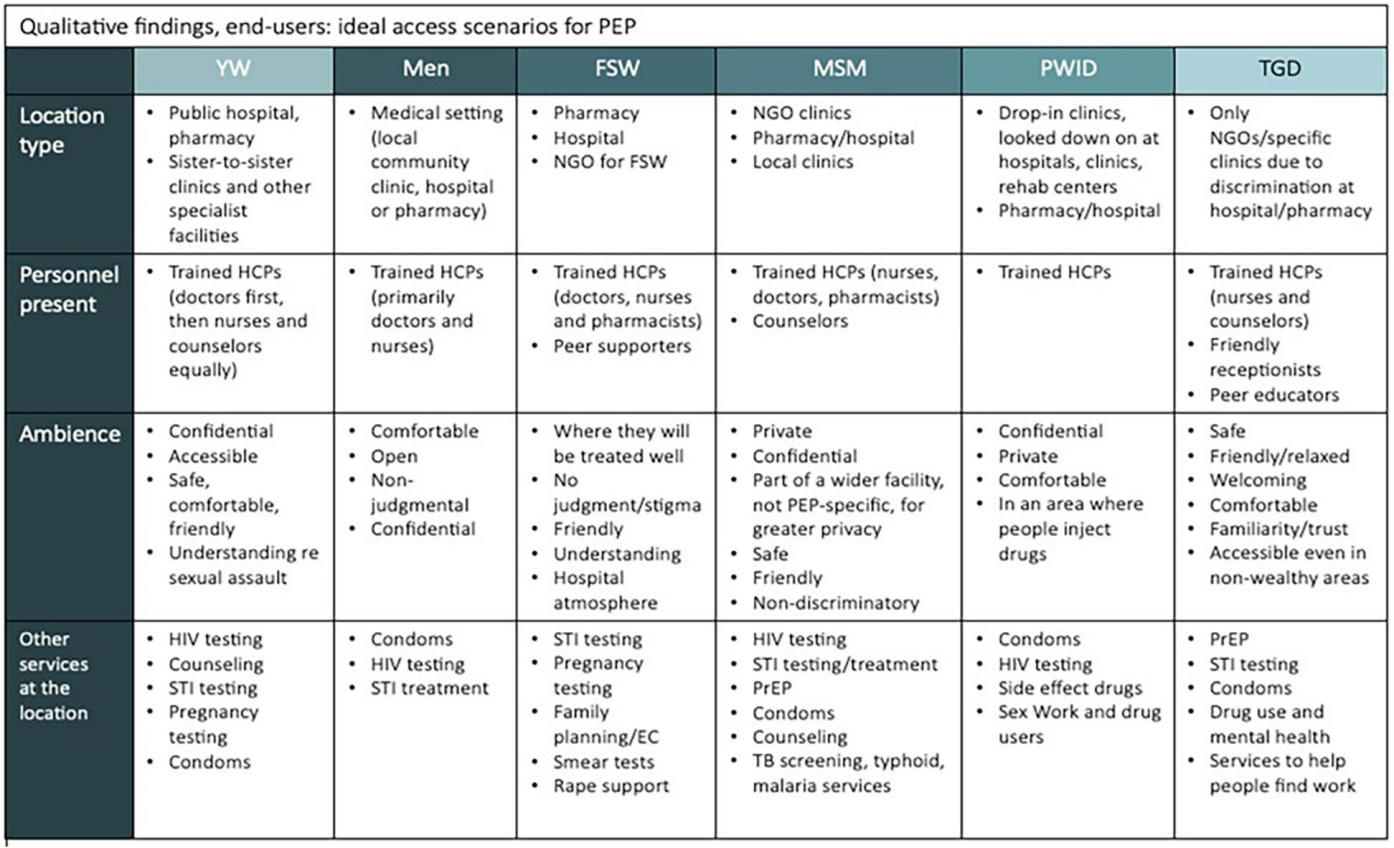
Ideal access scenarios for PEP across end-user groups (qualitative findings). Matrix summarizing what participants said would make PEP most accessible for them, by population group—location type, personnel present, ambience, and co-located services. Common preferences included access through routine clinical venues (hospitals, pharmacies, clinics), interaction with trained health-care providers, and private, non-judgmental settings. Group-specific notes: YW favored public hospitals/pharmacies and “sister-to-sister” clinics; men preferred general medical settings; FSW preferred pharmacies/hospitals and NGO facilities with peer supporters; MSM preferred NGO/local clinics and pharmacies; PWID emphasized drop-in or rehab-linked services in comfortable, stigma-free spaces; TGD favored NGO/specialty clinics due to discrimination in mainstream facilities, with friendly reception and peer educators. Desired co-services included HIV and STI testing/treatment, PrEP, condoms, pregnancy testing and emergency contraception, TB/malaria screening, mental-health and substance-use support, and services for sex workers and people who inject drugs. YW, young women; FSW, female sex workers; MSM, men who have sex with men; PWID, people who inject drugs; TGD, transgender and gender diverse; HCP, health-care provider; PrEP, pre-exposure prophylaxis.

*Qualitative* Figure 4 outlines the preferences of each group of respondents regarding their ideal scenarios for accessing PEP, including locations, personnel and environment.

The majority of end-users stated they would want to access PEP in a clinical setting:

“It would be either a clinic or hospital. There will be a qualified medical practitioner. It would be an enclosed, clean and well-equipped room.” Man aged 18–40

Through qualitative discussions it became clear that those prioritizing privacy, in particular respondents from key populations, felt uncomfortable with accessing PEP through community avenues:

“I'll go to the clinics but not the community facilities. The outreach is better than the community-based facilities. If you go to the community, everyone has their thoughts and can even tell people about your status.” Female Sex Worker

There were mixed responses to the technology-based options (vending machines and websites/apps), with those who were in favor praising the convenience, speed, non-time-limited access and perceived privacy. Objections unique to the technology-based options could be divided into two categories, principled and practical. Principled concerns included lack of counseling and ability to ask questions, lack of privacy (vending machines) and potential for misuse, especially for a sensitive and time-dependent intervention like PEP:

“Someone who doesn't have full information about it might dispense it, use it, maybe overdose or under-dose. It does not have the relevant information yet. It is not something like a rubber that you use and throw away. This is something that goes into your body, and it might affect the functioning of your body.” Transgender or Gender Diverse respondent

This highlights the need to balance innovation with safeguards that address user trust and support needs, particularly in contexts where stigma and misinformation may already undermine service uptake.

Practical concerns included whether the medication in machines would be in-date, whether the medication from websites or machines would be genuine, whether the websites would be trustworthy, and that there would be a need for reliable access to the internet:

“There are lots of fake websites and to avoid fraudsters who might be making wrong drugs.” Man aged 18–40

## 7 Discussion

The study results indicate that there is latent potential to expand access to and use of PEP for HIV prevention through increased awareness, broadened accessibility, and enhanced support for initiation and continuation. These findings align with WHO's recent recommendations and suggestions in the updated HIV PEP guidelines (1). This study contributes knowledge on PEP preferences, awareness and access among end-users. It also examines preferred PEP access points for different populations, evaluates the WHO PEP pathway, and provides recommendations for expanding access to PEP and support for continuation.

### 7.1 Awareness and acceptability of PEP

Recommendations for use of PEP to prevent HIV infection were developed decades ago, but awareness remains low (4, 7, 8, 10, 11, 15–19). Our study confirms low awareness of PEP with less than 40% of end-users in Kenya and Zimbabwe and less than 5% in Nigeria spontaneously identifying PEP as an HIV prevention method. When prompted, a greater proportion of respondents knew of PEP, particularly in Kenya (72.0%), but still less than 50% in Nigeria and Zimbabwe. In particular, Nigeria's notably low PEP awareness likely reflects broader structural challenges i.e., pervasive HIV-related stigma and limited access to tailored services for key populations in healthcare settings affecting uptake and engagement of such services (20). These findings reiterate the need to increase awareness and knowledge of PEP through multiple channels and to include key messages around when to seek PEP, urgency around initiation, and reassurance that PEP's benefits outweigh the downsides of taking it (10, 11, 15–19). This would facilitate greater uptake and completion of PEP, and potentially lower HIV transmission in the community (21, 22).

End-user acceptability of PEP has not been widely studied, but it has been found to be acceptable in some settings (15, 23). Our study found high acceptability, indicated by more than 80% of all end-users stating they are likely to use PEP if exposed to HIV. Furthermore, the PEP profile information was found to be useful, and in some cases, more useful than information received by those who had previously used PEP. Barriers such as poor counseling, limited ongoing support, misinformation or no information, and stigma against certain groups or behaviors can contribute to limited uptake or adherence of PEP (4, 16, 19, 24–31). This emphasizes a need for ongoing support to improve PEP uptake and completion.

### 7.2 PEP pathway

Our study illustrates the benefits and utility of WHO's PEP Pathway (assessment, counseling and support, prescription, and follow-up), with most end-users indicating that the steps are largely followed. End-users recommended consistent and appropriate support points throughout the pathway to ensure successful completion of the regimen and management of side effects. Fundamentally, this includes sharing more information about PEP in the counseling stage, such as the profile used in our study. Providing follow-up reminders or visits for medication management (and side effects) or other related needs are also recommended (32, 33). These findings align with other studies suggesting enhanced counseling and support for clients seeking PEP could increase continuation and improve clinical outcomes (11, 14, 28, 34, 35).

### 7.3 Broadening access

As with other HIV services, expanding access to PEP will require consideration of the needs and preferences of different populations and adaptation of delivery models for different needs. Our study reinforced this with a variety of preferred locations for accessing PEP identified. Most preferred a clinical setting, but the type varied from public hospitals or pharmacies for Young Women to community clinics for men to drop-in clinics for People Who Inject Drugs. Universally, end-users expressed the need for confidential, private services where they will be treated well without judgment or stigma, which was reiterated throughout the study and aligns with findings from other studies on the need to maintain privacy and confidentiality for PEP, and HIV services more broadly (7, 8, 19, 24, 25, 36–38).

Expanding access points for PEP will be important to increase use. Although our study found that most end-users prefer a general hospital for accessing PEP, this may be a reflection of the current PEP availability (19). Outreach and community-based outlets could be important for broadening access as end-users also value convenience and speed in accessing PEP. Community Health Workers (CHWs) may be well-placed to provide ongoing support and follow-up functions from the PEP pathway if the locations and providers exhibit other valued factors such as trustworthiness, confidentiality and knowledge of PEP. Other studies also point to the potential for expanded access to PEP through new channels including pharmacies and community-based distribution (16, 38–43). The least preferred locations for accessing PEP are point-of-sale outlets like vending machines or websites. Increased access to PEP through diverse, convenient locations shows promise, but more research is needed to understand and address end-user concerns related to privacy and confidentiality in these locations. These concerns are of high importance and must be addressed for any access point.

### 7.4 Strengths and limitations

Our study has many limitations as it focused on people's perception of PEP and, in some cases, their experience using PEP. The findings are largely formative, with the output intended to support WHO's updated PEP guidelines for expanding access through community-based channels and task-sharing. It was not within the scope of the study to identify any causal links to use or continuation of PEP. The themes and user insights identified are not generalizable or representative of the national populations, as the sample of participants was only a partial geographic sample within the target countries. It is likely that the views of some groups were not covered, and others may be under-represented in these data; furthermore, there could be confounding factors influencing preferences such as gender, age, and previous PEP experience.

### 7.5 Implications for practice and future research

This study confirms that PEP remains under-utilized as an HIV prevention method. WHO's new PEP guidelines recommend providing access at the community level and through task-sharing and suggest that consistent and appropriate support can improve uptake and adherence. Our study provides additional insights into the needs and preferences of different groups when accessing PEP and suggests that programs ensure access through diverse settings. The study also highlights low awareness of PEP and recommends generating broad awareness through diverse communication channels, as preferred information sources vary by group and country. Most importantly, the study highlights the need for multiple support points during the PEP pathway to ensure adherence. This will require leveraging the pathway to provide non-judgmental and informative access points for end-users to seek PEP and advice involving the community and other healthcare professionals who provide this kind of support efficiently (due to the 48–72 h window time restriction).

## 8 Conclusions

Preventing new HIV acquisition is becoming more challenging as incidence declines in many countries. Increasing access and use of PEP when someone has had a possible exposure is a promising approach to help close the gap on new acquisitions. Our study confirmed the overall appeal of PEP as a prevention method and offered insights into expanding access and improving uptake and continuation. WHO's recent update to the PEP guidelines recommends community-based distribution and task-sharing, which will help expand access points. Minimizing stigma associated with PEP use, however, will be critical for uptake, and this includes ensuring confidentiality and privacy of services, while increasing awareness and understanding of the prevention method. Additionally, provision of PEP from any type of provider needs to incorporate an empathetic approach and critical access points during PEP treatment to ensure correct and timely use, as well as to provide accurate information and emotional support for greater adherence. Our study highlights the different preferences among end-users regarding PEP, and future research should explore the programmatic requirements for offering PEP within a community through diverse channels and approaches to meet the needs of the different populations, as well as innovations to focus support points for greater continuation rates.

## Data Availability

The raw data supporting the conclusions of this article will be made available by the authors, without undue reservation.

## Supplementary material

Supplementary Table S3 Reported demographics: Relationship status, number of children, healthcare facility most often used, healthcare insurance, work status, highest level of schooling achieved, religion and regional split.

Supplementary Table S4 (PEP Experienced Users only): Recall of specific services offered within the PEP pathway.
