# Supplementary material for: Qualitative and quantitative research on preferences and perceptions regarding HIV post-exposure prophylaxis among young women, men, female sex workers, members of the LGBTQ + community and people who inject drugs in Kenya, Nigeria and Zimbabwe
Source: Front Reprod Health. 2025 Oct 9;7:1606013. doi: 10.3389/frph.2025.1606013 (PMC12546014; doi:10.3389/frph.2025.1606013)
Supplement: Supplementary Table S3 — Reported demographics: Relationship status, number of children, healthcare facility most often used, healthcare insurance, work status, highest level of schooling achieved, religion and regional split. [file Table1.docx]

| **S1. Perceived ease / difficulty of each step of the PEP pathway** | | | | | | | | | | | | | | | | | | | | | | | | | | | |
| --- | --- | --- | --- | --- | --- | --- | --- | --- | --- | --- | --- | --- | --- | --- | --- | --- | --- | --- | --- | --- | --- | --- | --- | --- | --- | --- | --- |
|  |  |  | **USERS** | **COUNTRY** | | | **KENYA** | | | | | **NIGERIA** | | | | | **ZIMBABWE** | | | | | **KENYA** | | **NIGERIA** | | **ZIMBABWE** | |
| **From PEP journey profile:** |  |  | **TOTAL** | **Kenya** | **Nigeria** | **Zimbabwe** | **TOTAL** | **YW** | **Men** | **MSM** | **FSW** | **TOTAL** | **YW** | **Men** | **MSM** | **FSW** | **TOTAL** | **YW** | **Men** | **MSM** | **FSW** | **PEP** | **NON-PEP** | **PEP** | **NON-PEP** | **PEP** | **NON-PEP** |
| **Showcard 3: Assessment** | **Assessment of potential HIV exposure** | **Base** | **904** | **302** | **305** | **297** | **302** | **100** | **96** | **56** | **50†** | **305** | **102** | **103** | **50†** | **50†** | **297** | **97** | **97** | **52** | **51** | **138** | **164** | **37†** | **268** | **113** | **184** |
|  | **Assessment of potential HIV exposure** | Very easy / easy | ***85.8%*** | *81.1%* | *93.4%^* | *82.8%* | ***81.1%*** | *78.0%* | *85.4%* | *85.7%* | *74.0%* | ***93.4%*** | *90.2%* | *91.3%* | *100.0%* | *98.0%* | ***82.8%*** | *77.3%* | *84.5%* | *90.4%* | *82.4%* | *89.1%** | *74.4%* | *100.0%* | *92.5%* | *81.4%* | *83.7%* |
|  | **Assessment of potential HIV exposure** | *Very easy* | ***37.8%*** | *28.8%§* | *42.0%* | *42.8%* | ***28.8%*** | *35.0%* | *30.2%* | *21.4%* | *22.0%* | ***42.0%*** | *31.4%* | *33.0%* | *66.0%* | *58.0%* | ***42.8%*** | *44.3%* | *43.3%* | *42.3%* | *39.2%* | *30.4%* | *27.4%* | *59.5%** | *39.6%* | *40.7%* | *44.0%* |
|  | **Assessment of potential HIV exposure** | *Easy* | ***48.0%*** | *52.3%* | *51.5%* | *40.1%§* | ***52.3%*** | *43.0%* | *55.2%* | *64.3%* | *52.0%* | ***51.5%*** | *58.8%* | *58.3%* | *34.0%* | *40.0%* | ***40.1%*** | *33.0%* | *41.2%* | *48.1%* | *43.1%* | *58.7%** | *47.0%* | *40.5%* | *53.0%* | *40.7%* | *39.7%* |
|  | **Assessment of potential HIV exposure** | Neutral | ***8.6%*** | *9.9%* | *2.3%§* | *13.8%* | ***9.9%*** | *15.0%* | *7.3%* | *8.9%* | *6.0%* | ***2.3%*** | *3.9%* | *2.9%* | *-* | *-* | ***13.8%*** | *18.6%* | *13.4%* | *3.8%* | *15.7%* | *5.1%* | *14.0%** | *-* | *2.6%* | *15.0%* | *13.0%* |
|  | **Assessment of potential HIV exposure** | Very difficult / difficult | ***3.4%*** | *6.6%^* | *1.0%* | *2.7%* | ***6.6%*** | *6.0%* | *7.3%* | *-* | *14.0%* | ***1.0%*** | *-* | *1.9%* | *-* | *2.0%* | ***2.7%*** | *3.1%* | *1.0%* | *5.8%* | *2.0%* | *5.8%* | *7.3%* | *-* | *1.1%* | *3.5%* | *2.2%* |
|  | **Assessment of potential HIV exposure** | *Difficult* | ***2.3%*** | *3.3%* | *1.0%* | *2.7%* | ***3.3%*** | *3.0%* | *3.1%* | *-* | *8.0%* | ***1.0%*** | *-* | *1.9%* | *-* | *2.0%* | ***2.7%*** | *3.1%* | *1.0%* | *5.8%* | *2.0%* | *2.9%* | *3.7%* | *-* | *1.1%* | *3.5%* | *2.2%* |
|  | **Assessment of potential HIV exposure** | *Very difficult* | ***1.1%*** | *3.3%^* | *-* | *-* | ***3.3%*** | *3.0%* | *4.2%* | *-* | *6.0%* | ***-*** | *-* | *-* | *-* | *-* | ***-*** | *-* | *-* | *-* | *-* | *2.9%* | *3.7%* | *-* | *-* | *-* | *-* |
|  | **Assessment of potential HIV exposure** | I do not know | ***2.1%*** | *2.3%* | *3.3%* | *0.7%* | ***2.3%*** | *1.0%* | *-* | *5.4%* | *6.0%* | ***3.3%*** | *5.9%* | *3.9%* | *-* | *-* | ***0.7%*** | *1.0%* | *1.0%* | *-* | *-* | *-* | *4%** | *-* | *3.7%* | *-* | *1.1%* |
|  | **Health check** | **Base** | **883** | **291** | **306** | **286** | **291** | **98** | **94** | **53** | **46†** | **306** | **101** | **104** | **51** | **50†** | **286** | **93** | **95** | **50†** | **48†** | **127** | **164** | **38†** | **268** | **102** | **184** |
|  | **Health check** | Very easy / easy | ***84.1%*** | *76.6%* | *96.4%^* | *78.7%* | ***76.6%*** | *71.4%* | *84.0%* | *81.1%* | *67.4%* | ***96.4%*** | *94.1%* | *96.2%* | *100.0%* | *98.0%* | ***78.7%*** | *79.6%* | *80.0%* | *78.0%* | *75.0%* | *84.3%** | *70.7%* | *97.4%* | *96.3%* | *85.3%** | *75.0%* |
|  | **Health check** | *Very easy* | ***34.4%*** | *31.3%* | *37.6%* | *34.3%* | ***31.3%*** | *35.7%* | *40.4%* | *17.0%* | *19.6%* | ***37.6%*** | *26.7%* | *26.9%* | *56.9%* | *62.0%* | ***34.3%*** | *37.6%* | *29.5%* | *38.0%* | *33.3%* | *29.9%* | *32.3%* | *57.9%** | *34.7%* | *40.2%* | *31.0%* |
|  | **Health check** | *Easy* | ***49.7%*** | *45.4%* | *58.8%^* | *44.4%* | ***45.4%*** | *35.7%* | *43.6%* | *64.2%* | *47.8%* | ***58.8%*** | *67.3%* | *69.2%* | *43.1%* | *36.0%* | ***44.4%*** | *41.9%* | *50.5%* | *40.0%* | *41.7%* | *54.3%** | *38.4%* | *39.5%* | *61.6%** | *45.1%* | *44.0%* |
|  | **Health check** | Neutral | ***10.2%*** | *13.4%* | *2.3%§* | *15.4%* | ***13.4%*** | *18.4%* | *8.5%* | *13.2%* | *13.0%* | ***2.3%*** | *4.0%* | *2.9%* | *-* | *-* | ***15.4%*** | *11.8%* | *17.9%* | *18.0%* | *14.6%* | *8.7%* | *17.1%** | *2.6%* | *2.2%* | *10.8%* | *17.9%* |
|  | **Health check** | Very difficult / difficult | ***3.7%*** | *7.2%^* | *1.0%* | *3.1%* | ***7.2%*** | *8.2%* | *7.4%* | *-* | *13.0%* | ***1.0%*** | *2.0%* | *1.0%* | *-* | *-* | ***3.1%*** | *5.4%* | *1.1%* | *2.0%* | *4.2%* | *7.1%* | *7.3%* | *-* | *1.1%* | *1.0%* | *4.3%* |
|  | **Health check** | *Difficult* | ***2.6%*** | *4.5%* | *0.7%§* | *2.8%* | ***4.5%*** | *7.1%* | *2.1%* | *-* | *8.7%* | ***0.7%*** | *1.0%* | *1.0%* | *-* | *-* | ***2.8%*** | *4.3%* | *1.1%* | *2.0%* | *4.2%* | *4.7%* | *4.3%* | *-* | *0.7%* | *1.0%* | *3.8%* |
|  | **Health check** | *Very difficult* | ***1.1%*** | *2.7%^* | *0.3%* | *0.3%* | ***2.7%*** | *1.0%* | *5.3%* | *-* | *4.3%* | ***0.3%*** | *1.0%* | *-* | *-* | *-* | ***0.3%*** | *1.1%* | *-* | *-* | *-* | *2.4%* | *3.0%* | *-* | *0.4%* | *-* | *0.5%* |
|  | **Health check** | I do not know | ***1.9%*** | *2.7%* | *0.3%§* | *2.8%* | ***2.7%*** | *2.0%* | *-* | *5.7%* | *6.5%* | ***0.3%*** | *-* | *-* | *-* | *2.0%* | ***2.8%*** | *3.2%* | *1.1%* | *2.0%* | *6.2%* | *-* | *4.9%** | *-* | *0.4%* | *2.9%* | *2.7%* |
|  | **HIV test** | **Base** | **915** | **305** | **306** | **304** | **305** | **99** | **99** | **57** | **50†** | **306** | **101** | **104** | **51** | **50†** | **304** | **100** | **100** | **52** | **52** | **141** | **164** | **38†** | **268** | **120** | **184** |
|  | **HIV test** | Very easy / easy | ***83.2%*** | *73.1%§* | *93.8%^* | *82.6%* | ***73.1%*** | *59.6%* | *83.8%* | *78.9%* | *72.0%* | ***93.8%*** | *91.1%* | *92.3%* | *98.0%* | *98.0%* | ***82.6%*** | *84.0%* | *79.0%* | *88.5%* | *80.8%* | *81.6%** | *65.9%* | *92.1%* | *94.0%* | *85.8%* | *80.4%* |
|  | **HIV test** | *Very easy* | ***34.4%*** | *25.6%* | *32.0%* | *45.7%^* | ***25.6%*** | *30.3%* | *29.3%* | *14.0%* | *22.0%* | ***32.0%*** | *30.7%* | *27.9%* | *35.3%* | *40.0%* | ***45.7%*** | *52.0%* | *46.0%* | *44.2%* | *34.6%* | *29.8%* | *22.0%* | *28.9%* | *32.5%* | *38.3%* | *50.5%** |
|  | **HIV test** | *Easy* | ***48.7%*** | *47.5%* | *61.8%^* | *36.8%§* | ***47.5%*** | *29.3%* | *54.5%* | *64.9%* | *50.0%* | ***61.8%*** | *60.4%* | *64.4%* | *62.7%* | *58.0%* | ***36.8%*** | *32.0%* | *33.0%* | *44.2%* | *46.2%* | *51.8%* | *43.9%* | *63.2%* | *61.6%* | *47.5%** | *29.9%* |
|  | **HIV test** | Neutral | ***10.2%*** | *12.8%* | *3.9%§* | *13.8%* | ***12.8%*** | *24.2%* | *5.1%* | *10.5%* | *8.0%* | ***3.9%*** | *7.9%* | *2.9%* | *2.0%* | *-* | ***13.8%*** | *8.0%* | *19.0%* | *11.5%* | *17.3%* | *7.8%* | *17.1%** | *2.6%* | *4.1%* | *12.5%* | *14.7%* |
|  | **HIV test** | Very difficult / difficult | ***6.1%*** | *12.8%^* | *2.3%* | *3.3%* | ***12.8%*** | *15.2%* | *11.1%* | *8.8%* | *16.0%* | ***2.3%*** | *1.0%* | *4.8%* | *-* | *2.0%* | ***3.3%*** | *8.0%* | *2.0%* | *-* | *-* | *10.6%* | *14.6%* | *5.3%* | *1.9%* | *1.7%* | *4.3%* |
|  | **HIV test** | *Difficult* | ***3.0%*** | *3.6%* | *2.3%* | *3.0%* | ***3.6%*** | *6.1%* | *5.1%* | *-* | *-* | ***2.3%*** | *1.0%* | *4.8%* | *-* | *2.0%* | ***3.0%*** | *7.0%* | *2.0%* | *-* | *-* | *4.3%* | *3.0%* | *5.3%* | *1.9%* | *0.8%* | *4.3%* |
|  | **HIV test** | *Very difficult* | ***3.2%*** | *9.2%^* | *-* | *0.3%* | ***9.2%*** | *9.1%* | *6.1%* | *8.8%* | *16.0%* | ***-*** | *-* | *-* | *-* | *-* | ***0.3%*** | *1.0%* | *-* | *-* | *-* | *6.4%* | *11.6%* | *-* | *-* | *0.8%* | *-* |
|  | **HIV test** | I do not know | ***0.5%*** | *1.3%* | *-* | *0.3%* | ***1.3%*** | *1.0%* | *-* | *1.8%* | *4.0%* | ***-*** | *-* | *-* | *-* | *-* | ***0.3%*** | *-* | *-* | *-* | *1.9%* | *-* | *2.4%* | *-* | *-* | *-* | *0.5%* |
|  | **First aid in case of broken skin or other wound** | **Base** | **764** | **252** | **280** | **232** | **252** | **83** | **80** | **50†** | **39†** | **280** | **94** | **92** | **48†** | **46†** | **232** | **72** | **70** | **45†** | **45†** | **88** | **164** | **12†** | **268** | **†** | **184** |
|  | **First aid in case of broken skin or other wound** | Very easy / easy | ***83.4%*** | *75.8%* | *93.2%^* | *79.7%* | ***75.8%*** | *74.7%* | *76.2%* | *80.0%* | *71.8%* | ***93.2%*** | *92.6%* | *89.1%* | *95.8%* | *100.0%* | ***79.7%*** | *73.6%* | *75.7%* | *93.3%* | *82.2%* | *85.2%** | *70.7%* | *75.0%* | *94.0%** | *85.4%* | *78.3%* |
|  | **First aid in case of broken skin or other wound** | *Very easy* | ***34.9%*** | *28.2%* | *43.6%^* | *31.9%* | ***28.2%*** | *27.7%* | *32.5%* | *18.0%* | *33.3%* | ***43.6%*** | *36.2%* | *34.8%* | *60.4%* | *58.7%* | ***31.9%*** | *25.0%* | *30.0%* | *42.2%* | *35.6%* | *29.5%* | *27.4%* | *33.3%* | *44.0%* | *37.5%* | *30.4%* |
|  | **First aid in case of broken skin or other wound** | *Easy* | ***48.4%*** | *47.6%* | *49.6%* | *47.8%* | ***47.6%*** | *47.0%* | *43.8%* | *62.0%* | *38.5%* | ***49.6%*** | *56.4%* | *54.3%* | *35.4%* | *41.3%* | ***47.8%*** | *48.6%* | *45.7%* | *51.1%* | *46.7%* | *55.7%* | *43.3%* | *41.7%* | *50.0%* | *47.9%* | *47.8%* |
|  | **First aid in case of broken skin or other wound** | Neutral | ***11.4%*** | *16.3%* | *6.1%§* | *12.5%* | ***16.3%*** | *16.9%* | *18.8%* | *16.0%* | *10.3%* | ***6.1%*** | *7.4%* | *9.8%* | *2.1%* | *-* | ***12.5%*** | *11.1%* | *17.1%* | *4.4%* | *15.6%* | *13.6%* | *17.7%* | *16.7%* | *5.6%* | *4.2%* | *14.7%* |
|  | **First aid in case of broken skin or other wound** | Very difficult / difficult | ***2.1%*** | *3.6%* | *0.4%§* | *2.6%* | ***3.6%*** | *6.0%* | *1.2%* | *-* | *7.7%* | ***0.4%*** | *-* | *1.1%* | *-* | *-* | ***2.6%*** | *5.6%* | *1.4%* | *2.2%* | *-* | *1.1%* | *4.9%* | *-* | *0.4%* | *-* | *3.3%* |
|  | **First aid in case of broken skin or other wound** | *Difficult* | ***1.6%*** | *2.4%* | *0.4%* | *2.2%* | ***2.4%*** | *3.6%* | *1.2%* | *-* | *5.1%* | ***0.4%*** | *-* | *1.1%* | *-* | *-* | ***2.2%*** | *4.2%* | *1.4%* | *2.2%* | *-* | *1.1%* | *3.0%* | *-* | *0.4%* | *-* | *2.7%* |
|  | **First aid in case of broken skin or other wound** | *Very difficult* | ***0.5%*** | *1.2%* | *-* | *0.4%* | ***1.2%*** | *2.4%* | *-* | *-* | *2.6%* | ***-*** | *-* | *-* | *-* | *-* | ***0.4%*** | *1.4%* | *-* | *-* | *-* | *-* | *1.8%* | *-* | *-* | *-* | *0.5%* |
|  | **First aid in case of broken skin or other wound** | I do not know | ***3.1%*** | *4.4%* | *0.4%§* | *5.2%* | ***4.4%*** | *2.4%* | *3.8%* | *4.0%* | *10.3%* | ***0.4%*** | *-* | *-* | *2.1%* | *-* | ***5.2%*** | *9.7%* | *5.7%* | *-* | *2.2%* | *-* | *6.7%** | *8.3%** | *-* | *10.4%* | *3.8%* |
| **From PEP journey profile:  Showcard 3: Counselling and support** | **Risk of HIV discussed** | **Base** | **901** | **297** | **303** | **301** | **297** | **100** | **98** | **54** | **45** | **303** | **102** | **102** | **50†** | **49†** | **301** | **100** | **98** | **51** | **52** | **133** | **164** | **35†** | **268** | **117** | **184** |
|  | **Risk of HIV discussed** | Very easy / easy | ***88.6%*** | *86.2%* | *95.0%^* | *84.4%* | ***86.2%*** | *83.0%* | *89.8%* | *90.7%* | *80.0%* | ***95.0%*** | *91.2%* | *94.1%* | *100.0%* | *100.0%* | ***84.4%*** | *81.0%* | *85.7%* | *86.3%* | *86.5%* | *91.0%** | *82.3%* | *97.1%* | *94.8%* | *82.1%* | *85.9%* |
|  | **Risk of HIV discussed** | *Very easy* | ***37.5%*** | *32.0%* | *43.9%* | *36.5%* | ***32.0%*** | *39.0%* | *34.7%* | *18.5%* | *26.7%* | ***43.9%*** | *40.2%* | *36.3%* | *62.0%* | *49.0%* | ***36.5%*** | *34.0%* | *34.7%* | *51.0%* | *30.8%* | *33.1%* | *31.1%* | *57.1%* | *42.2%* | *38.5%* | *35.3%* |
|  | **Risk of HIV discussed** | *Easy* | ***51.1%*** | *54.2%* | *51.2%* | *47.8%* | ***54.2%*** | *44.0%* | *55.1%* | *72.2%* | *53.3%* | ***51.2%*** | *51.0%* | *57.8%* | *38.0%* | *51.0%* | ***47.8%*** | *47.0%* | *51.0%* | *35.3%* | *55.8%* | *57.9%* | *51.2%* | *40.0%* | *52.6%* | *43.6%* | *50.5%* |
|  | **Risk of HIV discussed** | Neutral | ***9.1%*** | *10.1%* | *4.6%§* | *12.6%* | ***10.1%*** | *15.0%* | *9.2%* | *3.7%* | *8.9%* | ***4.6%*** | *8.8%* | *4.9%* | *-* | *-* | ***12.6%*** | *14.0%* | *12.2%* | *11.8%* | *11.5%* | *8.3%* | *11.6%* | *2.9%* | *4.9%* | *16.2%* | *10.3%* |
|  | **Risk of HIV discussed** | Very difficult / difficult | ***1.1%*** | *1.3%* | *0.3%* | *1.7%* | ***1.3%*** | *1.0%* | *1.0%* | *1.9%* | *2.2%* | ***0.3%*** | *-* | *1.0%* | *-* | *-* | ***1.7%*** | *2.0%* | *1.0%* | *2.0%* | *1.9%* | *0.8%* | *1.8%* | *-* | *0.4%* | *0.9%* | *2.2%* |
|  | **Risk of HIV discussed** | *Difficult* | ***1.0%*** | *1.0%* | *0.3%* | *1.7%* | ***1.0%*** | *-* | *1.0%* | *1.9%* | *2.2%* | ***0.3%*** | *-* | *1.0%* | *-* | *-* | ***1.7%*** | *2.0%* | *1.0%* | *2.0%* | *1.9%* | *0.8%* | *1.2%* | *-* | *0.4%* | *0.9%* | *2.2%* |
|  | **Risk of HIV discussed** | *Very difficult* | ***0.1%*** | *0.3%* | *-* | *-* | ***0.3%*** | *1.0%* | *-* | *-* | *-* | ***-*** | *-* | *-* | *-* | *-* | ***-*** | *-* | *-* | *-* | *-* | *-* | *0.6%* | *-* | *-* | *-* | *-* |
|  | **Risk of HIV discussed** | I do not know | ***1.2%*** | *2.4%* | *-* | *1.3%* | ***2.4%*** | *1.0%* | *-* | *3.7%* | *8.9%* | ***-*** | *-* | *-* | *-* | *-* | ***1.3%*** | *3.0%* | *1.0%* | *-* | *-* | *-* | *4.3%** | *-* | *-* | *0.9%* | *1.6%* |
|  | **Risk and benefits of HIV PEP explained to you** | **Base** | **908** | **304** | **301** | **303** | **304** | **99** | **98** | **56** | **51** | **301** | **99** | **102** | **50†** | **50†** | **303** | **100** | **99** | **52** | **52** | **140** | **164** | **33†** | **268** | **119** | **184** |
|  | **Risk and benefits of HIV PEP explained to you** | Very easy / easy | ***91.1%*** | *87.2%* | *97.0%^* | *89.1%* | ***87.2%*** | *88.9%* | *86.7%* | *83.9%* | *88.2%* | ***97.0%*** | *96.0%* | *96.1%* | *98.0%* | *100.0%* | ***89.1%*** | *86.0%* | *88.9%* | *92.3%* | *92.3%* | *92.9%** | *82.3%* | *97.0%* | *97.0%* | *87.4%* | *90.2%* |
|  | **Risk and benefits of HIV PEP explained to you** | *Very easy* | ***40.3%*** | *33.2%§* | *44.5%* | *43.2%* | ***33.2%*** | *38.4%* | *33.7%* | *19.6%* | *37.3%* | ***44.5%*** | *41.4%* | *33.3%* | *66.0%* | *52.0%* | ***43.2%*** | *46.0%* | *46.5%* | *46.2%* | *28.8%* | *37.1%* | *29.9%* | *48.5%* | *44.0%* | *45.4%* | *41.8%* |
|  | **Risk and benefits of HIV PEP explained to you** | *Easy* | ***50.8%*** | *53.9%* | *52.5%* | *45.9%* | ***53.9%*** | *50.5%* | *53.1%* | *64.3%* | *51.0%* | ***52.5%*** | *54.5%* | *62.7%* | *32.0%* | *48.0%* | ***45.9%*** | *40.0%* | *42.4%* | *46.2%* | *63.5%* | *55.7%* | *52.4%* | *48.5%* | *53.0%* | *42.0%* | *48.4%* |
|  | **Risk and benefits of HIV PEP explained to you** | Neutral | ***7.4%*** | *9.9%* | *3.0%§* | *9.2%* | ***9.9%*** | *10.1%* | *12.2%* | *10.7%* | *3.9%* | ***3.0%*** | *4.0%* | *3.9%* | *2.0%* | *-* | ***9.2%*** | *10.0%* | *10.1%* | *7.7%* | *7.7%* | *6.4%* | *12.8%* | *3.0%* | *3.0%* | *10.9%* | *8.2%* |
|  | **Risk and benefits of HIV PEP explained to you** | Very difficult / difficult | ***0.3%*** | *0.7%* | *-* | *0.3%* | ***0.7%*** | *-* | *1.0%* | *-* | *2.0%* | ***-*** | *-* | *-* | *-* | *-* | ***0.3%*** | *1.0%* | *-* | *-* | *-* | *0.7%* | *0.6%* | *-* | *-* | *0.8%* | *-* |
|  | **Risk and benefits of HIV PEP explained to you** | *Difficult* | ***0.3%*** | *0.7%* | *-* | *0.3%* | ***0.7%*** | *-* | *1.0%* | *-* | *2.0%* | ***-*** | *-* | *-* | *-* | *-* | ***0.3%*** | *1.0%* | *-* | *-* | *-* | *0.7%* | *0.6%* | *-* | *-* | *0.8%* | *-* |
|  | **Risk and benefits of HIV PEP explained to you** | *Very difficult* | ***-*** | *-* | *-* | *-* | ***-*** | *-* | *-* | *-* | *-* | ***-*** | *-* | *-* | *-* | *-* | ***-*** | *-* | *-* | *-* | *-* | *-* | *-* | *-* | *-* | *-* | *-* |
|  | **Risk and benefits of HIV PEP explained to you** | I do not know | ***1.2%*** | *2.3%* | *-* | *1.3%* | ***2.3%*** | *1.0%* | *-* | *5.4%* | *5.9%* | ***-*** | *-* | *-* | *-* | *-* | ***1.3%*** | *3.0%* | *1.0%* | *-* | *-* | *-* | *4.3%** | *-* | *-* | *0.8%* | *1.6%* |
|  | **Side effects described** | **Base** | **904** | **300** | **303** | **301** | **300** | **97** | **98** | **56** | **49†** | **303** | **101** | **103** | **50†** | **49†** | **301** | **99** | **98** | **52** | **52** | **136** | **164** | **35†** | **268** | **117** | **184** |
|  | **Side effects described** | Very easy / easy | ***83.3%*** | *80.7%* | *91.4%^* | *77.7%* | ***80.7%*** | *77.3%* | *84.7%* | *76.8%* | *83.7%* | ***91.4%*** | *90.1%* | *87.4%* | *96.0%* | *98.0%* | ***77.7%*** | *76.8%* | *79.6%* | *84.6%* | *69.2%* | *89.0%** | *73.8%* | *94.3%* | *91.0%* | *81.2%* | *75.5%* |
|  | **Side effects described** | *Very easy* | ***35.1%*** | *31.0%* | *36.0%* | *38.2%* | ***31.0%*** | *35.1%* | *37.8%* | *19.6%* | *22.4%* | ***36.0%*** | *30.7%* | *26.2%* | *56.0%* | *46.9%* | ***38.2%*** | *36.4%* | *44.9%* | *44.2%* | *23.1%* | *31.6%* | *30.5%* | *42.9%* | *35.1%* | *39.3%* | *37.5%* |
|  | **Side effects described** | *Easy* | ***48.2%*** | *49.7%* | *55.4%* | *39.5%§* | ***49.7%*** | *42.3%* | *46.9%* | *57.1%* | *61.2%* | ***55.4%*** | *59.4%* | *61.2%* | *40.0%* | *51.0%* | ***39.5%*** | *40.4%* | *34.7%* | *40.4%* | *46.2%* | *57.4%** | *43.3%* | *51.4%* | *56.0%* | *41.9%* | *38.0%* |
|  | **Side effects described** | Neutral | ***13.6%*** | *14.3%* | *7.3%§* | *19.3%* | ***14.3%*** | *20.6%* | *11.2%* | *16.1%* | *6.1%* | ***7.3%*** | *9.9%* | *10.7%* | *-* | *2.0%* | ***19.3%*** | *16.2%* | *18.4%* | *15.4%* | *30.8%* | *10.3%* | *17.7%* | *2.9%* | *7.8%* | *17.1%* | *20.7%* |
|  | **Side effects described** | Very difficult / difficult | ***2.1%*** | *2.7%* | *1.3%* | *2.3%* | ***2.7%*** | *1.0%* | *4.1%* | *1.8%* | *4.1%* | ***1.3%*** | *-* | *1.9%* | *4.0%* | *-* | ***2.3%*** | *6.1%* | *1.0%* | *-* | *-* | *0.7%* | *4.3%* | *2.9%* | *1.1%* | *1.7%* | *2.7%* |
|  | **Side effects described** | *Difficult* | ***1.9%*** | *2.3%* | *1.3%* | *2.0%* | ***2.3%*** | *1.0%* | *4.1%* | *-* | *4.1%* | ***1.3%*** | *-* | *1.9%* | *4.0%* | *-* | ***2.0%*** | *5.1%* | *1.0%* | *-* | *-* | *0.7%* | *3.7%* | *2.9%* | *1.1%* | *0.9%* | *2.7%* |
|  | **Side effects described** | *Very difficult* | ***0.2%*** | *0.3%* | *-* | *0.3%* | ***0.3%*** | *-* | *-* | *1.8%* | *-* | ***-*** | *-* | *-* | *-* | *-* | ***0.3%*** | *1.0%* | *-* | *-* | *-* | *-* | *0.6%* | *-* | *-* | *0.9%* | *-* |
|  | **Side effects described** | I do not know | ***1.0%*** | *2.3%* | *-* | *0.7%* | ***2.3%*** | *1.0%* | *-* | *5.4%* | *6.1%* | ***-*** | *-* | *-* | *-* | *-* | ***0.7%*** | *1.0%* | *1.0%* | *-* | *-* | *-* | *4%** | *-* | *-* | *-* | *1.1%* |
|  | **Counselling on how to take and keep taking PEP for 28 days** | **Base** | **909** | **304** | **305** | **300** | **304** | **101** | **98** | **56** | **49†** | **305** | **101** | **103** | **51** | **50†** | **300** | **98** | **100** | **52** | **50†** | **140** | **164** | **37†** | **268** | **116** | **184** |
|  | **Counselling on how to take and keep taking PEP for 28 days** | Very easy / easy | ***89.7%*** | *86.2%* | *93.4%* | *89.3%* | ***86.2%*** | *85.1%* | *90.8%* | *83.9%* | *81.6%* | ***93.4%*** | *90.1%* | *91.3%* | *100.0%* | *98.0%* | ***89.3%*** | *89.8%* | *89.0%* | *98.1%* | *80.0%* | *90.7%** | *82.3%* | *94.6%* | *93.3%* | *94.0%** | *86.4%* |
|  | **Counselling on how to take and keep taking PEP for 28 days** | *Very easy* | ***42.0%*** | *38.5%* | *43.3%* | *44.3%* | ***38.5%*** | *45.5%* | *39.8%* | *17.9%* | *44.9%* | ***43.3%*** | *31.7%* | *36.9%* | *60.8%* | *62.0%* | ***44.3%*** | *45.9%* | *46.0%* | *44.2%* | *38.0%* | *37.9%* | *39.0%* | *59.5%** | *41.0%* | *48.3%* | *41.8%* |
|  | **Counselling on how to take and keep taking PEP for 28 days** | *Easy* | ***47.6%*** | *47.7%* | *50.2%* | *45.0%* | ***47.7%*** | *39.6%* | *51.0%* | *66.1%* | *36.7%* | ***50.2%*** | *58.4%* | *54.4%* | *39.2%* | *36.0%* | ***45.0%*** | *43.9%* | *43.0%* | *53.8%* | *42.0%* | *52.9%* | *43.3%* | *35.1%* | *52.2%* | *45.7%* | *44.6%* |
|  | **Counselling on how to take and keep taking PEP for 28 days** | Neutral | ***8.1%*** | *8.9%* | *5.6%* | *10.0%* | ***8.9%*** | *10.9%* | *7.1%* | *7.1%* | *10.2%* | ***5.6%*** | *8.9%* | *7.8%* | *-* | *-* | ***10.0%*** | *10.2%* | *9.0%* | *1.9%* | *20.0%* | *7.9%* | *9.8%* | *2.7%* | *6.0%* | *5.2%* | *13.0%** |
|  | **Counselling on how to take and keep taking PEP for 28 days** | Very difficult / difficult | ***1.3%*** | *2.6%* | *1.0%* | *0.3%* | ***2.6%*** | *3.0%* | *2.0%* | *3.6%* | *2.0%* | ***1.0%*** | *1.0%* | *1.0%* | *-* | *2.0%* | ***0.3%*** | *-* | *1.0%* | *-* | *-* | *1.4%* | *3.7%* | *2.7%* | *0.7%* | *0.9%* | *-* |
|  | **Counselling on how to take and keep taking PEP for 28 days** | *Difficult* | ***1.3%*** | *2.6%* | *1.0%* | *0.3%* | ***2.6%*** | *3.0%* | *2.0%* | *3.6%* | *2.0%* | ***1.0%*** | *1.0%* | *1.0%* | *-* | *2.0%* | ***0.3%*** | *-* | *1.0%* | *-* | *-* | *1.4%* | *3.7%* | *2.7%* | *0.7%* | *0.9%* | *-* |
|  | **Counselling on how to take and keep taking PEP for 28 days** | *Very difficult* | ***-*** | *-* | *-* | *-* | ***-*** | *-* | *-* | *-* | *-* | ***-*** | *-* | *-* | *-* | *-* | ***-*** | *-* | *-* | *-* | *-* | *-* | *-* | *-* | *-* | *-* | *-* |
|  | **Counselling on how to take and keep taking PEP for 28 days** | I do not know | ***0.9%*** | *2.3%^* | *-* | *0.3%* | ***2.3%*** | *1.0%* | *-* | *5.4%* | *6.1%* | ***-*** | *-* | *-* | *-* | *-* | ***0.3%*** | *-* | *1.0%* | *-* | *-* | *-* | *4%** | *-* | *-* | *-* | *0.5%* |
|  | **Specific support in case of sexual assault** | **Base** | **788** | **258** | **285** | **245** | **258** | **89** | **82** | **46†** | **41†** | **285** | **97** | **92** | **48†** | **48†** | **245** | **76** | **75** | **47†** | **47†** | **94** | **164** | **17†** | **268** | **61** | **184** |
|  | **Specific support in case of sexual assault** | Very easy / easy | ***77.2%*** | *73.6%* | *83.2%^* | *73.9%* | ***73.6%*** | *70.8%* | *76.8%* | *73.9%* | *73.2%* | ***83.2%*** | *78.4%* | *75.0%* | *95.8%* | *95.8%* | ***73.9%*** | *73.7%* | *66.7%* | *83.0%* | *76.6%* | *81.9%** | *68.9%* | *88.2%* | *82.8%* | *60.7%* | *78.3%** |
|  | **Specific support in case of sexual assault** | *Very easy* | ***28.7%*** | *28.3%* | *27.0%* | *31.0%* | ***28.3%*** | *31.5%* | *31.7%* | *13.0%* | *31.7%* | ***27.0%*** | *10.3%* | *19.6%* | *58.3%* | *43.8%* | ***31.0%*** | *31.6%* | *30.7%* | *27.7%* | *34.0%* | *29.8%* | *27.4%* | *23.5%* | *27.2%* | *27.9%* | *32.1%* |
|  | **Specific support in case of sexual assault** | *Easy* | ***48.5%*** | *45.3%* | *56.1%^* | *42.9%* | ***45.3%*** | *39.3%* | *45.1%* | *60.9%* | *41.5%* | ***56.1%*** | *68.0%* | *55.4%* | *37.5%* | *52.1%* | ***42.9%*** | *42.1%* | *36.0%* | *55.3%* | *42.6%* | *52.1%* | *41.5%* | *64.7%* | *55.6%* | *32.8%* | *46.2%* |
|  | **Specific support in case of sexual assault** | Neutral | ***14.1%*** | *16.7%* | *7.4%§* | *19.2%* | ***16.7%*** | *16.9%* | *17.1%* | *19.6%* | *12.2%* | ***7.4%*** | *8.2%* | *10.9%* | *4.2%* | *2.1%* | ***19.2%*** | *11.8%* | *29.3%* | *10.6%* | *23.4%* | *11.7%* | *19.5%* | *11.8%* | *7.1%* | *31.0%** | *15.2%* |
|  | **Specific support in case of sexual assault** | Very difficult / difficult | ***3.9%*** | *6.2%* | *3.5%* | *2.0%* | ***6.2%*** | *10.1%* | *4.9%* | *-* | *7.3%* | ***3.5%*** | *3.1%* | *6.5%* | *-* | *2.1%* | ***2.0%*** | *6.6%* | *-* | *-* | *-* | *6.4%* | *6.1%* | *-* | *3.7%* | *-* | *2.7%* |
|  | **Specific support in case of sexual assault** | *Difficult* | ***2.7%*** | *3.5%* | *2.8%* | *1.6%* | ***3.5%*** | *5.6%* | *1.2%* | *-* | *7.3%* | ***2.8%*** | *3.1%* | *4.3%* | *-* | *2.1%* | ***1.6%*** | *5.3%* | *-* | *-* | *-* | *1.1%* | *4.9%* | *-* | *3.0%* | *-* | *2.2%* |
|  | **Specific support in case of sexual assault** | *Very difficult* | ***1.3%*** | *2.7%* | *0.7%* | *0.4%* | ***2.7%*** | *4.5%* | *3.7%* | *-* | *-* | ***0.7%*** | *-* | *2.2%* | *-* | *-* | ***0.4%*** | *1.3%* | *-* | *-* | *-* | *5.3%* | *1.2%* | *-* | *0.7%* | *-* | *0.5%* |
|  | **Specific support in case of sexual assault** | I do not know | ***4.8%*** | *3.5%* | *6.0%* | *4.9%* | ***3.5%*** | *2.2%* | *1.2%* | *6.5%* | *7.3%* | ***6.0%*** | *10.3%* | *7.6%* | *-* | *-* | ***4.9%*** | *7.9%* | *4.0%* | *6.4%* | *-* | *-* | *5.5%** | *-* | *6.3%* | *8.2%* | *3.8%* |
| **From PEP journey profile:  Showcard 3: Prescription** | **PEP initiated as early as possible following exposure (within 72 hours)** | **Base** | **907** | **304** | **305** | **298** | **304** | **100** | **99** | **56** | **49†** | **305** | **101** | **104** | **50†** | **50†** | **298** | **98** | **97** | **51** | **52** | **140** | **164** | **37†** | **268** | **114** | **184** |
|  | **PEP initiated as early as possible following exposure (within 72 hours)** | Very easy / easy | ***88.0%*** | *84.5%* | *94.4%^* | *84.9%* | ***84.5%*** | *83.0%* | *87.9%* | *83.9%* | *81.6%* | ***94.4%*** | *90.1%* | *94.2%* | *98.0%* | *100.0%* | ***84.9%*** | *79.6%* | *87.6%* | *90.2%* | *84.6%* | *85.0%* | *84.1%* | *97.3%* | *94.0%* | *92.1%** | *80.4%* |
|  | **PEP initiated as early as possible following exposure (within 72 hours)** | *Very easy* | ***37.9%*** | *34.9%* | *32.1%* | *47.0%^* | ***34.9%*** | *45.0%* | *34.3%* | *19.6%* | *32.7%* | ***32.1%*** | *31.7%* | *24.0%* | *36.0%* | *46.0%* | ***47.0%*** | *41.8%* | *46.4%* | *58.8%* | *46.2%* | *31.4%* | *37.8%* | *45.9%* | *30.2%* | *54.4%** | *42.4%* |
|  | **PEP initiated as early as possible following exposure (within 72 hours)** | *Easy* | ***50.1%*** | *49.7%* | *62.3%^* | *37.9%§* | ***49.7%*** | *38.0%* | *53.5%* | *64.3%* | *49.0%* | ***62.3%*** | *58.4%* | *70.2%* | *62.0%* | *54.0%* | ***37.9%*** | *37.8%* | *41.2%* | *31.4%* | *38.5%* | *53.6%* | *46.3%* | *51.4%* | *63.8%* | *37.7%* | *38.0%* |
|  | **PEP initiated as early as possible following exposure (within 72 hours)** | Neutral | ***9.0%*** | *12.2%* | *4.3%§* | *10.7%* | ***12.2%*** | *15.0%* | *10.1%* | *10.7%* | *12.2%* | ***4.3%*** | *6.9%* | *4.8%* | *2.0%* | *-* | ***10.7%*** | *17.3%* | *10.3%* | *2.0%* | *7.7%* | *14.3%* | *10.4%* | *2.7%* | *4.5%* | *7.9%* | *12.5%* |
|  | **PEP initiated as early as possible following exposure (within 72 hours)** | Very difficult / difficult | ***0.9%*** | *0.7%* | *1.3%* | *0.7%* | ***0.7%*** | *1.0%* | *1.0%* | *-* | *-* | ***1.3%*** | *3.0%* | *1.0%* | *-* | *-* | ***0.7%*** | *1.0%* | *1.0%* | *-* | *-* | *0.7%* | *0.6%* | *-* | *1.5%* | *-* | *1.1%* |
|  | **PEP initiated as early as possible following exposure (within 72 hours)** | *Difficult* | ***0.9%*** | *0.7%* | *1.3%* | *0.7%* | ***0.7%*** | *1.0%* | *1.0%* | *-* | *-* | ***1.3%*** | *3.0%* | *1.0%* | *-* | *-* | ***0.7%*** | *1.0%* | *1.0%* | *-* | *-* | *0.7%* | *0.6%* | *-* | *1.5%* | *-* | *1.1%* |
|  | **PEP initiated as early as possible following exposure (within 72 hours)** | *Very difficult* | ***-*** | *-* | *-* | *-* | ***-*** | *-* | *-* | *-* | *-* | ***-*** | *-* | *-* | *-* | *-* | ***-*** | *-* | *-* | *-* | *-* | *-* | *-* | *-* | *-* | *-* | *-* |
|  | **PEP initiated as early as possible following exposure (within 72 hours)** | I do not know | ***2.1%*** | *2.6%* | *-* | *3.7%* | ***2.6%*** | *1.0%* | *1.0%* | *5.4%* | *6.1%* | ***-*** | *-* | *-* | *-* | *-* | ***3.7%*** | *2.0%* | *1.0%* | *7.8%* | *7.7%* | *-* | *4.9%** | *-* | *-* | *-* | *6.0%** |
|  | **28-day prescription written and provided** | **Base** | **913** | **309** | **302** | **302** | **309** | **101** | **100** | **57** | **51** | **302** | **99** | **102** | **51** | **50†** | **302** | **99** | **99** | **52** | **52** | **145** | **164** | **34†** | **268** | **118** | **184** |
|  | **28-day prescription written and provided** | Very easy / easy | ***91.7%*** | *89.0%* | *93.0%* | *93.0%* | ***89.0%*** | *91.1%* | *90.0%* | *86.0%* | *86.3%* | ***93.0%*** | *86.9%* | *92.2%* | *100.0%* | *100.0%* | ***93.0%*** | *91.9%* | *90.9%* | *100.0%* | *92.3%* | *89.7%* | *88.4%* | *97.1%* | *92.5%* | *94.9%* | *91.8%* |
|  | **28-day prescription written and provided** | *Very easy* | ***36.1%*** | *33.0%* | *31.1%* | *44.4%^* | ***33.0%*** | *39.6%* | *34.0%* | *26.3%* | *25.5%* | ***31.1%*** | *21.2%* | *21.6%* | *49.0%* | *52.0%* | ***44.4%*** | *36.4%* | *49.5%* | *51.9%* | *42.3%* | *27.6%* | *37.8%* | *38.2%* | *30.2%* | *53.4%** | *38.6%* |
|  | **28-day prescription written and provided** | *Easy* | ***55.5%*** | *56.0%* | *61.9%* | *48.7%* | ***56.0%*** | *51.5%* | *56.0%* | *59.6%* | *60.8%* | ***61.9%*** | *65.7%* | *70.6%* | *51.0%* | *48.0%* | ***48.7%*** | *55.6%* | *41.4%* | *48.1%* | *50.0%* | *62.1%** | *50.6%* | *58.8%* | *62.3%* | *41.5%* | *53.3%** |
|  | **28-day prescription written and provided** | Neutral | ***5.9%*** | *8.1%* | *4.6%* | *5.0%* | ***8.1%*** | *7.9%* | *8.0%* | *8.8%* | *7.8%* | ***4.6%*** | *9.1%* | *4.9%* | *-* | *-* | ***5.0%*** | *5.1%* | *6.1%* | *-* | *7.7%* | *9.0%* | *7.3%* | *2.9%* | *4.9%* | *4.2%* | *5.4%* |
|  | **28-day prescription written and provided** | Very difficult / difficult | ***1.2%*** | *0.6%* | *2.0%* | *1.0%* | ***0.6%*** | *-* | *2.0%* | *-* | *-* | ***2.0%*** | *3.0%* | *2.9%* | *-* | *-* | ***1.0%*** | *1.0%* | *2.0%* | *-* | *-* | *1.4%* | *-* | *-* | *2.2%* | *0.8%* | *1.1%* |
|  | **28-day prescription written and provided** | *Difficult* | ***1.1%*** | *0.6%* | *1.7%* | *1.0%* | ***0.6%*** | *-* | *2.0%* | *-* | *-* | ***1.7%*** | *3.0%* | *2.0%* | *-* | *-* | ***1.0%*** | *1.0%* | *2.0%* | *-* | *-* | *1.4%* | *-* | *-* | *1.9%* | *0.8%* | *1.1%* |
|  | **28-day prescription written and provided** | *Very difficult* | ***0.1%*** | *-* | *0.3%* | *-* | ***-*** | *-* | *-* | *-* | *-* | ***0.3%*** | *-* | *1.0%* | *-* | *-* | ***-*** | *-* | *-* | *-* | *-* | *-* | *-* | *-* | *0.4%* | *-* | *-* |
|  | **28-day prescription written and provided** | I do not know | ***1.2%*** | *2.3%* | *0.3%* | *1.0%* | ***2.3%*** | *1.0%* | *-* | *5.3%* | *5.9%* | ***0.3%*** | *1.0%* | *-* | *-* | *-* | ***1.0%*** | *2.0%* | *1.0%* | *-* | *-* | *-* | *4%** | *-* | *0.4%* | *-* | *1.6%* |
|  | **Drug information given** | **Base** | **897** | **295** | **306** | **296** | **295** | **99** | **94** | **54** | **48†** | **306** | **101** | **104** | **51** | **50†** | **296** | **96** | **97** | **51** | **52** | **131** | **164** | **38†** | **268** | **112** | **184** |
|  | **Drug information given** | Very easy / easy | ***92.0%*** | *87.1%* | *98.4%^* | *90.2%* | ***87.1%*** | *84.8%* | *91.5%* | *85.2%* | *85.4%* | ***98.4%*** | *99.0%* | *99.0%* | *100.0%* | *94.0%* | ***90.2%*** | *89.6%* | *86.6%* | *96.1%* | *92.3%* | *92.4%** | *82.9%* | *100.0%* | *98.1%* | *92.0%* | *89.1%* |
|  | **Drug information given** | *Very easy* | ***38.6%*** | *36.3%* | *36.6%* | *42.9%* | ***36.3%*** | *46.5%* | *37.2%* | *20.4%* | *31.2%* | ***36.6%*** | *31.7%* | *29.8%* | *58.8%* | *38.0%* | ***42.9%*** | *43.8%* | *42.3%* | *52.9%* | *32.7%* | *32.1%* | *39.6%* | *50.0%* | *34.7%* | *46.4%* | *40.8%* |
|  | **Drug information given** | *Easy* | ***53.4%*** | *50.8%* | *61.8%^* | *47.3%* | ***50.8%*** | *38.4%* | *54.3%* | *64.8%* | *54.2%* | ***61.8%*** | *67.3%* | *69.2%* | *41.2%* | *56.0%* | ***47.3%*** | *45.8%* | *44.3%* | *43.1%* | *59.6%* | *60.3%** | *43.3%* | *50.0%* | *63.4%* | *45.5%* | *48.4%* |
|  | **Drug information given** | Neutral | ***5.8%*** | *8.1%* | *1.3%§* | *8.1%* | ***8.1%*** | *13.1%* | *5.3%* | *7.4%* | *4.2%* | ***1.3%*** | *-* | *1.0%* | *-* | *6.0%* | ***8.1%*** | *8.3%* | *11.3%* | *2.0%* | *7.7%* | *5.3%* | *10.4%* | *-* | *1.5%* | *6.2%* | *9.2%* |
|  | **Drug information given** | Very difficult / difficult | ***1.1%*** | *2.4%* | *-* | *1.0%* | ***2.4%*** | *1.0%* | *3.2%* | *1.9%* | *4.2%* | ***-*** | *-* | *-* | *-* | *-* | ***1.0%*** | *-* | *2.1%* | *2.0%* | *-* | *2.3%* | *2.4%* | *-* | *-* | *1.8%* | *0.5%* |
|  | **Drug information given** | *Difficult* | ***0.9%*** | *2.0%* | *-* | *0.7%* | ***2.0%*** | *-* | *3.2%* | *1.9%* | *4.2%* | ***-*** | *-* | *-* | *-* | *-* | ***0.7%*** | *-* | *2.1%* | *-* | *-* | *2.3%* | *1.8%* | *-* | *-* | *0.9%* | *0.5%* |
|  | **Drug information given** | *Very difficult* | ***0.2%*** | *0.3%* | *-* | *0.3%* | ***0.3%*** | *1.0%* | *-* | *-* | *-* | ***-*** | *-* | *-* | *-* | *-* | ***0.3%*** | *-* | *-* | *2.0%* | *-* | *-* | *0.6%* | *-* | *-* | *0.9%* | *-* |
|  | **Drug information given** | I do not know | ***1.1%*** | *2.4%* | *0.3%* | *0.7%* | ***2.4%*** | *1.0%* | *-* | *5.6%* | *6.2%* | ***0.3%*** | *1.0%* | *-* | *-* | *-* | ***0.7%*** | *2.1%* | *-* | *-* | *-* | *-* | *4%** | *-* | *0.4%* | *-* | *1.1%* |
|  | **Assessment of health conditions and any other medications** | **Base** | **865** | **285** | **301** | **279** | **285** | **90** | **95** | **53** | **47†** | **301** | **99** | **101** | **51** | **50†** | **279** | **92** | **89** | **50** | **48†** | **121** | **164** | **33†** | **268** | **95** | **184** |
|  | **Assessment of health conditions and any other medications** | Very easy / easy | ***82.0%*** | *79.6%* | *93.7%^* | *71.7%§* | ***79.6%*** | *73.3%* | *83.2%* | *79.2%* | *85.1%* | ***93.7%*** | *90.9%* | *93.1%* | *96.1%* | *98.0%* | ***71.7%*** | *78.3%* | *68.5%* | *64.0%* | *72.9%* | *88.4%** | *73.2%* | *97.0%* | *93.3%* | *77.9%* | *68.5%* |
|  | **Assessment of health conditions and any other medications** | *Very easy* | ***26.9%*** | *27.4%* | *26.2%* | *27.2%* | ***27.4%*** | *32.2%* | *30.5%* | *9.4%* | *31.9%* | ***26.2%*** | *18.2%* | *21.8%* | *39.2%* | *38.0%* | ***27.2%*** | *26.1%* | *23.6%* | *36.0%* | *27.1%* | *26.4%* | *28.0%* | *27.3%* | *26.1%* | *28.4%* | *26.6%* |
|  | **Assessment of health conditions and any other medications** | *Easy* | ***55.0%*** | *52.3%* | *67.4%^* | *44.4%* | ***52.3%*** | *41.1%* | *52.6%* | *69.8%* | *53.2%* | ***67.4%*** | *72.7%* | *71.3%* | *56.9%* | *60.0%* | ***44.4%*** | *52.2%* | *44.9%* | *28.0%* | *45.8%* | *62.0%** | *45.1%* | *69.7%* | *67.2%* | *49.5%* | *41.8%* |
|  | **Assessment of health conditions and any other medications** | Neutral | ***13.8%*** | *14.7%* | *5.3%§* | *21.9%^* | ***14.7%*** | *18.9%* | *13.7%* | *15.1%* | *8.5%* | ***5.3%*** | *8.1%* | *5.9%* | *2.0%* | *2.0%* | ***21.9%*** | *15.2%* | *27.0%* | *34.0%* | *12.5%* | *8.3%* | *20%** | *-* | *6.0%* | *21.1%* | *22.3%* |
|  | **Assessment of health conditions and any other medications** | Very difficult / difficult | ***2.3%*** | *3.2%* | *0.7%§* | *3.2%* | ***3.2%*** | *6.7%* | *3.2%* | *-* | *-* | ***0.7%*** | *1.0%* | *-* | *2.0%* | *-* | ***3.2%*** | *5.4%* | *3.4%* | *-* | *2.1%* | *3.3%* | *3.0%* | *-* | *0.7%* | *1.1%* | *4.3%* |
|  | **Assessment of health conditions and any other medications** | *Difficult* | ***2.1%*** | *2.8%* | *0.7%§* | *2.9%* | ***2.8%*** | *5.6%* | *3.2%* | *-* | *-* | ***0.7%*** | *1.0%* | *-* | *2.0%* | *-* | ***2.9%*** | *5.4%* | *2.2%* | *-* | *2.1%* | *3.3%* | *2.4%* | *-* | *0.7%* | *1.1%* | *3.8%* |
|  | **Assessment of health conditions and any other medications** | *Very difficult* | ***0.2%*** | *0.4%* | *-* | *0.4%* | ***0.4%*** | *1.1%* | *-* | *-* | *-* | ***-*** | *-* | *-* | *-* | *-* | ***0.4%*** | *-* | *1.1%* | *-* | *-* | *-* | *0.6%* | *-* | *-* | *-* | *0.5%* |
|  | **Assessment of health conditions and any other medications** | I do not know | ***2.0%*** | *2.5%* | *0.3%§* | *3.2%* | ***2.5%*** | *1.1%* | *-* | *5.7%* | *6.4%* | ***0.3%*** | *-* | *1.0%* | *-* | *-* | ***3.2%*** | *1.1%* | *1.1%* | *2.0%* | *12.5%* | *-* | *4.3%** | *3.0%** | *-* | *-* | *4.9%** |
| **From PEP journey profile:  Showcard 3: Follow-up** | **HIV test at 3 months after exposure** | **Base** | **881** | **291** | **296** | **294** | **291** | **94** | **95** | **56** | **46†** | **296** | **97** | **101** | **49†** | **49†** | **294** | **95** | **96** | **52** | **51** | **127** | **164** | **28†** | **268** | **110** | **184** |
|  | **HIV test at 3 months after exposure** | Very easy / easy | ***82.7%*** | *77.0%* | *89.2%^* | *82.0%* | ***77.0%*** | *74.5%* | *78.9%* | *83.9%* | *69.6%* | ***89.2%*** | *84.5%* | *87.1%* | *95.9%* | *95.9%* | ***82.0%*** | *78.9%* | *83.3%* | *86.5%* | *80.4%* | *83.5%** | *72.0%* | *92.9%* | *88.8%* | *82.7%* | *81.5%* |
|  | **HIV test at 3 months after exposure** | *Very easy* | ***28.9%*** | *24.7%* | *24.7%* | *37.4%^* | ***24.7%*** | *29.8%* | *29.5%* | *14.3%* | *17.4%* | ***24.7%*** | *18.6%* | *14.9%* | *44.9%* | *36.7%* | ***37.4%*** | *31.6%* | *37.5%* | *48.1%* | *37.3%* | *26.0%* | *23.8%* | *39.3%* | *23.1%* | *35.5%* | *38.6%* |
|  | **HIV test at 3 months after exposure** | *Easy* | ***53.8%*** | *52.2%* | *64.5%^* | *44.6%* | ***52.2%*** | *44.7%* | *49.5%* | *69.6%* | *52.2%* | ***64.5%*** | *66.0%* | *72.3%* | *51.0%* | *59.2%* | ***44.6%*** | *47.4%* | *45.8%* | *38.5%* | *43.1%* | *57.5%* | *48.2%* | *53.6%* | *65.7%* | *47.3%* | *42.9%* |
|  | **HIV test at 3 months after exposure** | Neutral | ***12.1%*** | *16.5%* | *6.1%§* | *13.9%* | ***16.5%*** | *22.3%* | *11.6%* | *10.7%* | *21.7%* | ***6.1%*** | *8.2%* | *8.9%* | *-* | *2.0%* | ***13.9%*** | *12.6%* | *12.5%* | *13.5%* | *19.6%* | *11.0%* | *20.7%** | *3.6%* | *6.3%* | *14.5%* | *13.6%* |
|  | **HIV test at 3 months after exposure** | Very difficult / difficult | ***3.6%*** | *3.4%* | *4.4%* | *3.1%* | ***3.4%*** | *2.1%* | *7.4%* | *-* | *2.2%* | ***4.4%*** | *6.2%* | *4.0%* | *4.1%* | *2.0%* | ***3.1%*** | *6.3%* | *3.1%* | *-* | *-* | *4.7%* | *2.4%* | *3.6%* | *4.5%* | *1.8%* | *3.8%* |
|  | **HIV test at 3 months after exposure** | *Difficult* | ***2.7%*** | *1.7%* | *3.7%* | *2.7%* | ***1.7%*** | *1.1%* | *4.2%* | *-* | *-* | ***3.7%*** | *4.1%* | *4.0%* | *4.1%* | *2.0%* | ***2.7%*** | *6.3%* | *2.1%* | *-* | *-* | *3.1%* | *0.6%* | *3.6%* | *3.7%* | *1.8%* | *3.3%* |
|  | **HIV test at 3 months after exposure** | *Very difficult* | ***0.9%*** | *1.7%* | *0.7%* | *0.3%* | ***1.7%*** | *1.1%* | *3.2%* | *-* | *2.2%* | ***0.7%*** | *2.1%* | *-* | *-* | *-* | ***0.3%*** | *-* | *1.0%* | *-* | *-* | *1.6%* | *1.8%* | *-* | *0.7%* | *-* | *0.5%* |
|  | **HIV test at 3 months after exposure** | I do not know | ***1.5%*** | *3.1%* | *0.3%* | *1.0%* | ***3.1%*** | *1.1%* | *2.1%* | *5.4%* | *6.5%* | ***0.3%*** | *1.0%* | *-* | *-* | *-* | ***1.0%*** | *2.1%* | *1.0%* | *-* | *-* | *0.8%* | *5%** | *-* | *0.4%* | *0.9%* | *1.1%* |
|  | **Link to HIV treatment if needed** | **Base** | **824** | **267** | **284** | **273** | **267** | **90** | **87** | **47†** | **43†** | **284** | **94** | **93** | **48†** | **49†** | **273** | **87** | **88** | **48** | **50†** | **103** | **164** | **16†** | **268** | **89** | **184** |
|  | **Link to HIV treatment if needed** | Very easy / easy | ***85.3%*** | *80.5%* | *92.3%^* | *82.8%* | ***80.5%*** | *77.8%* | *82.8%* | *80.9%* | *81.4%* | ***92.3%*** | *89.4%* | *91.4%* | *97.9%* | *93.9%* | ***82.8%*** | *80.5%* | *83.0%* | *87.5%* | *82.0%* | *88.3%** | *75.6%* | *93.8%* | *92.2%* | *87.6%* | *80.4%* |
|  | **Link to HIV treatment if needed** | *Very easy* | ***30.0%*** | *32.6%* | *21.8%§* | *35.9%* | ***32.6%*** | *37.8%* | *34.5%* | *17.0%* | *34.9%* | ***21.8%*** | *21.3%* | *18.3%* | *22.9%* | *28.6%* | ***35.9%*** | *40.2%* | *35.2%* | *35.4%* | *30.0%* | *37.9%* | *29.3%* | *25.0%* | *21.6%* | *32.6%* | *37.5%* |
|  | **Link to HIV treatment if needed** | *Easy* | ***55.3%*** | *47.9%* | *70.4%^* | *46.9%* | ***47.9%*** | *40.0%* | *48.3%* | *63.8%* | *46.5%* | ***70.4%*** | *68.1%* | *73.1%* | *75.0%* | *65.3%* | ***46.9%*** | *40.2%* | *47.7%* | *52.1%* | *52.0%* | *50.5%* | *46.3%* | *68.8%* | *70.5%* | *55.1%* | *42.9%* |
|  | **Link to HIV treatment if needed** | Neutral | ***11.8%*** | *14.2%* | *6.3%* | *15.0%* | ***14.2%*** | *17.8%* | *13.8%* | *12.8%* | *9.3%* | ***6.3%*** | *7.4%* | *7.5%* | *2.1%* | *6.1%* | ***15.0%*** | *16.1%* | *13.6%* | *12.5%* | *18.0%* | *9.7%* | *17.1%* | *6.2%* | *6.3%* | *11.2%* | *16.8%* |
|  | **Link to HIV treatment if needed** | Very difficult / difficult | ***1.1%*** | *1.5%* | *0.7%* | *1.1%* | ***1.5%*** | *2.2%* | *1.1%* | *-* | *2.3%* | ***0.7%*** | *2.1%* | *-* | *-* | *-* | ***1.1%*** | *2.3%* | *1.1%* | *-* | *-* | *1.0%* | *1.8%* | *-* | *0.7%* | *-* | *1.6%* |
|  | **Link to HIV treatment if needed** | *Difficult* | ***0.7%*** | *0.7%* | *0.7%* | *0.7%* | ***0.7%*** | *1.1%* | *1.1%* | *-* | *-* | ***0.7%*** | *2.1%* | *-* | *-* | *-* | ***0.7%*** | *2.3%* | *-* | *-* | *-* | *-* | *1.2%* | *-* | *0.7%* | *-* | *1.1%* |
|  | **Link to HIV treatment if needed** | *Very difficult* | ***0.4%*** | *0.7%* | *-* | *0.4%* | ***0.7%*** | *1.1%* | *-* | *-* | *2.3%* | ***-*** | *-* | *-* | *-* | *-* | ***0.4%*** | *-* | *1.1%* | *-* | *-* | *1.0%* | *0.6%* | *-* | *-* | *-* | *0.5%* |
|  | **Link to HIV treatment if needed** | I do not know | ***1.8%*** | *3.7%^* | *0.7%* | *1.1%* | ***3.7%*** | *2.2%* | *2.3%* | *6.4%* | *7.0%* | ***0.7%*** | *1.1%* | *1.1%* | *-* | *-* | ***1.1%*** | *1.1%* | *2.3%* | *-* | *-* | *1.0%* | *5.5%* | *-* | *0.7%* | *1.1%* | *1.1%* |
|  | **Any other HIV prevention provided if needed (e.g. PrEP)** | **Base** | **864** | **293** | **288** | **283** | **293** | **92** | **96** | **56** | **49†** | **288** | **97** | **97** | **47†** | **47†** | **283** | **89** | **92** | **52** | **50†** | **129** | **164** | **20†** | **268** | **99** | **184** |
|  | **Any other HIV prevention provided if needed (e.g. PrEP)** | Very easy / easy | ***90.0%*** | *85.7%§* | *92.0%* | *92.6%* | ***85.7%*** | *85.9%* | *88.5%* | *83.9%* | *81.6%* | ***92.0%*** | *91.8%* | *89.7%* | *95.7%* | *93.6%* | ***92.6%*** | *91.0%* | *93.5%* | *96.2%* | *90.0%* | *90.7%** | *81.7%* | *90.0%* | *92.2%* | *94.9%* | *91.3%* |
|  | **Any other HIV prevention provided if needed (e.g. PrEP)** | *Very easy* | ***33.4%*** | *32.8%* | *23.6%§* | *44.2%^* | ***32.8%*** | *39.1%* | *35.4%* | *21.4%* | *28.6%* | ***23.6%*** | *21.6%* | *21.6%* | *38.3%* | *17.0%* | ***44.2%*** | *41.6%* | *45.7%* | *51.9%* | *38.0%* | *34.1%* | *31.7%* | *15.0%* | *24.3%* | *51.5%* | *40.2%* |
|  | **Any other HIV prevention provided if needed (e.g. PrEP)** | *Easy* | ***56.6%*** | *52.9%* | *68.4%* | *48.4%* | ***52.9%*** | *46.7%* | *53.1%* | *62.5%* | *53.1%* | ***68.4%*** | *70.1%* | *68.0%* | *57.4%* | *76.6%* | ***48.4%*** | *49.4%* | *47.8%* | *44.2%* | *52.0%* | *56.6%* | *50.0%* | *75.0%* | *67.9%* | *43.4%* | *51.1%* |
|  | **Any other HIV prevention provided if needed (e.g. PrEP)** | Neutral | ***7.3%*** | *9.6%* | *6.6%* | *5.7%* | ***9.6%*** | *10.9%* | *8.3%* | *8.9%* | *10.2%* | ***6.6%*** | *6.2%* | *9.3%* | *4.3%* | *4.3%* | ***5.7%*** | *5.6%* | *5.4%* | *3.8%* | *8.0%* | *5.4%* | *13%** | *5.0%* | *6.7%* | *3.0%* | *7.1%* |
|  | **Any other HIV prevention provided if needed (e.g. PrEP)** | Very difficult / difficult | ***0.5%*** | *1.4%* | *-* | *-* | ***1.4%*** | *1.1%* | *2.1%* | *-* | *2.0%* | ***-*** | *-* | *-* | *-* | *-* | ***-*** | *-* | *-* | *-* | *-* | *2.3%* | *0.6%* | *-* | *-* | *-* | *-* |
|  | **Any other HIV prevention provided if needed (e.g. PrEP)** | *Difficult* | ***0.5%*** | *1.4%* | *-* | *-* | ***1.4%*** | *1.1%* | *2.1%* | *-* | *2.0%* | ***-*** | *-* | *-* | *-* | *-* | ***-*** | *-* | *-* | *-* | *-* | *2.3%* | *0.6%* | *-* | *-* | *-* | *-* |
|  | **Any other HIV prevention provided if needed (e.g. PrEP)** | *Very difficult* | ***-*** | *-* | *-* | *-* | ***-*** | *-* | *-* | *-* | *-* | ***-*** | *-* | *-* | *-* | *-* | ***-*** | *-* | *-* | *-* | *-* | *-* | *-* | *-* | *-* | *-* | *-* |
|  | **Any other HIV prevention provided if needed (e.g. PrEP)** | I do not know | ***2.2%*** | *3.4%* | *1.4%* | *1.8%* | ***3.4%*** | *2.2%* | *1.0%* | *7.1%* | *6.1%* | ***1.4%*** | *2.1%* | *1.0%* | *-* | *2.1%* | ***1.8%*** | *3.4%* | *1.1%* | *-* | *2.0%* | *1.6%* | *4.9%* | *5.0%* | *1.1%* | *2.0%* | *1.6%* |
| *Abbreviations: HIV, Human Immunodeficiency Disease, PEP, Post-Exposure Prophylaxis; PrEP, Pre-Exposure Prophylaxis; YW, Young Women. NB. Key Population includes Female Sex Workers and Men who have Sex with Men. § Significantly lower than the other 2 countries, ^ Significantly higher than the other 2 countries. * Statistically higher than PEP/Non-PEP sample. † Low base size.* | | | | | | | | | | | | | | | | | | | | | | | | | | | |

| **S2. Preferred places to access PEP** | | | | | | | | | | | | | | | | | | | | | | | | | | |
| --- | --- | --- | --- | --- | --- | --- | --- | --- | --- | --- | --- | --- | --- | --- | --- | --- | --- | --- | --- | --- | --- | --- | --- | --- | --- | --- |
|  |  | **USERS** | **COUNTRY** | | | **KENYA** | | | | | **NIGERIA** | | | | | **ZIMBABWE** | | | | | **KENYA** | | **NIGERIA** | | **ZIMBABWE** | |
|  |  | **TOTAL** | **Kenya** | **Nigeria** | **Zimbabwe** | **TOTAL** | **YW** | **Men** | **MSM** | **FSW** | **TOTAL** | **YW** | **Men** | **MSM** | **FSW** | **TOTAL** | **YW** | **Men** | **MSM** | **FSW** | **PEP** | **NON-PEP** | **PEP** | **NON-PEP** | **PEP** | **NON-PEP** |
|  | **Base** | **920** | **309** | **307** | **304** | **309** | **101** | **100** | **57** | **51** | **307** | **102** | **104** | **51** | **50** | **304** | **100** | **100** | **52** | **52** | **145** | **164** | **39†** | **268** | **120** | **184** |
| **General hospital** | Yes | **81.2%** | 73.1%§ | 86.6% | 83.9% | **73.1%** | 77.2% | 74.0% | 63.2% | 74.5% | **86.6%** | 82.4% | 87.5% | 92.2% | 88.0% | **83.9%** | 93.0% | 92.0% | 48.1% | 86.5% | 72.4% | 73.8% | 92.3% | 85.8% | 86.7% | 82.1% |
| **General hospital** | No | **14.1%** | 21.4%^ | 8.1% | 12.8% | **21.4%** | 16.8% | 19.0% | 35.1% | 19.6% | **8.1%** | 11.8% | 7.7% | 2.0% | 8.0% | **12.8%** | 4.0% | 5.0% | 50.0% | 7.7% | 24.1% | 18.9% | 7.7% | 8.2% | 9.2% | 15.2% |
| **General hospital** | Maybe | **4.7%** | 5.5% | 5.2% | 3.3% | **5.5%** | 5.9% | 7.0% | 1.8% | 5.9% | **5.2%** | 5.9% | 4.8% | 5.9% | 4.0% | **3.3%** | 3.0% | 3.0% | 1.9% | 5.8% | 3.4% | 7.3% | - | 6.0% | 4.2% | 2.7% |
| **A clinic** | Yes | **74.8%** | 63.4%§ | 74.9% | 86.2%^ | **63.4%** | 65.3% | 67.0% | 56.1% | 60.8% | **74.9%** | 70.6% | 80.8% | 82.4% | 64.0% | **86.2%** | 92.0% | 87.0% | 75.0% | 84.6% | 67.6% | 59.8% | 69.2% | 75.7% | 95.0%* | 80.4% |
| **A clinic** | No | **18.2%** | 23.6% | 18.6% | 12.2%§ | **23.6%** | 18.8% | 20.0% | 29.8% | 33.3% | **18.6%** | 21.6% | 12.5% | 13.7% | 30.0% | **12.2%** | 6.0% | 11.0% | 25.0% | 13.5% | 21.4% | 25.6% | 28.2% | 17.2% | 4.2% | 17.4%* |
| **A clinic** | Maybe | **7.1%** | 12.9%^ | 6.5% | 1.6%§ | **12.9%** | 15.8% | 13.0% | 14.0% | 5.9% | **6.5%** | 7.8% | 6.7% | 3.9% | 6.0% | **1.6%** | 2.0% | 2.0% | - | 1.9% | 11.0% | 14.6% | 2.6% | 7.1% | 0.8% | 2.2% |
| **An independent pharmacy** | Yes | **65.3%** | 39.2%§ | 72.3% | 84.9%^ | **39.2%** | 30.7% | 50.0% | 40.4% | 33.3% | **72.3%** | 62.7% | 66.3% | 92.2% | 84.0% | **84.9%** | 81.0% | 85.0% | 94.2% | 82.7% | 42.8% | 36.0% | 53.8% | 75.0%* | 91.7%* | 80.4% |
| **An independent pharmacy** | No | **22.9%** | 39.8%^ | 17.9% | 10.9%§ | **39.8%** | 45.5% | 28.0% | 42.1% | 49.0% | **17.9%** | 27.5% | 24.0% | - | 4.0% | **10.9%** | 11.0% | 11.0% | 5.8% | 15.4% | 41.4% | 38.4% | 28.2% | 16.4% | 6.7% | 13.6% |
| **An independent pharmacy** | Maybe | **11.7%** | 21.0%^ | 9.8% | 4.3%§ | **21.0%** | 23.8% | 22.0% | 17.5% | 17.6% | **9.8%** | 9.8% | 9.6% | 7.8% | 12.0% | **4.3%** | 8.0% | 4.0% | - | 1.9% | 15.9% | 25.6%* | 17.9% | 8.6% | 1.7% | 6.0% |
| **A pharmacy at a hospital** | Yes | **64.3%** | 46.6%§ | 69.7% | 77.0%^ | **46.6%** | 42.6% | 49.0% | 49.1% | 47.1% | **69.7%** | 69.6% | 66.3% | 76.5% | 70.0% | **77.0%** | 85.0% | 85.0% | 50.0% | 73.1% | 48.3% | 45.1% | 66.7% | 70.1% | 85.0%* | 71.7% |
| **A pharmacy at a hospital** | No | **24.9%** | 37.5%^ | 18.6% | 18.4% | **37.5%** | 35.6% | 34.0% | 40.4% | 45.1% | **18.6%** | 17.6% | 20.2% | 13.7% | 22.0% | **18.4%** | 8.0% | 11.0% | 50.0% | 21.2% | 37.9% | 37.2% | 25.6% | 17.5% | 12.5% | 22.3%* |
| **A pharmacy at a hospital** | Maybe | **10.8%** | 15.9% | 11.7% | 4.6%§ | **15.9%** | 21.8% | 17.0% | 10.5% | 7.8% | **11.7%** | 12.7% | 13.5% | 9.8% | 8.0% | **4.6%** | 7.0% | 4.0% | - | 5.8% | 13.8% | 17.7% | 7.7% | 12.3% | 2.5% | 6.0% |
| **An HIV clinic** | Yes | **63.9%** | 70.2% | 46.6%§ | 75.0% | **70.2%** | 62.4% | 72.0% | 77.2% | 74.5% | **46.6%** | 46.1% | 42.3% | 54.9% | 48.0% | **75.0%** | 75.0% | 76.0% | 57.7% | 90.4% | 75.9%* | 65.2% | 59.0% | 44.8% | 80.8% | 71.2% |
| **An HIV clinic** | No | **27.5%** | 20.1% | 43.6%^ | 18.8% | **20.1%** | 25.7% | 19.0% | 17.5% | 13.7% | **43.6%** | 47.1% | 51.9% | 21.6% | 42.0% | **18.8%** | 17.0% | 17.0% | 36.5% | 7.7% | 15.9% | 23.8% | 35.9% | 44.8% | 12.5% | 22.8%* |
| **An HIV clinic** | Maybe | **8.6%** | 9.7% | 9.8% | 6.2% | **9.7%** | 11.9% | 9.0% | 5.3% | 11.8% | **9.8%** | 6.9% | 5.8% | 23.5% | 10.0% | **6.2%** | 8.0% | 7.0% | 5.8% | 1.9% | 8.3% | 11.0% | 5.1% | 10.4% | 6.7% | 6.0% |
| **A sexual health clinic** | Yes | **63.7%** | 70.9% | 45.0%§ | 75.3% | **70.9%** | 60.4% | 74.0% | 82.5% | 72.5% | **45.0%** | 40.2% | 41.3% | 56.9% | 50.0% | **75.3%** | 68.0% | 78.0% | 67.3% | 92.3% | 75.9% | 66.5% | 48.7% | 44.4% | 81.7%* | 71.2% |
| **A sexual health clinic** | No | **25.1%** | 17.5% | 42.7%^ | 15.1% | **17.5%** | 24.8% | 14.0% | 12.3% | 15.7% | **42.7%** | 51.0% | 48.1% | 23.5% | 34.0% | **15.1%** | 17.0% | 11.0% | 26.9% | 7.7% | 15.2% | 19.5% | 38.5% | 43.3% | 10.8% | 17.9% |
| **A sexual health clinic** | Maybe | **11.2%** | 11.7% | 12.4% | 9.5% | **11.7%** | 14.9% | 12.0% | 5.3% | 11.8% | **12.4%** | 8.8% | 10.6% | 19.6% | 16.0% | **9.5%** | 15.0% | 11.0% | 5.8% | - | 9.0% | 14.0% | 12.8% | 12.3% | 7.5% | 10.9% |
| **Community-based clinic** | Yes | **57.6%** | 39.5%§ | 57.7% | 76.0%^ | **39.5%** | 30.7% | 41.0% | 36.8% | 56.9% | **57.7%** | 57.8% | 50.0% | 70.6% | 60.0% | **76.0%** | 83.0% | 76.0% | 46.2% | 92.3% | 42.8% | 36.6% | 43.6% | 59.7% | 82.5%* | 71.7% |
| **Community-based clinic** | No | **29.0%** | 35.9% | 32.2% | 18.8%§ | **35.9%** | 39.6% | 32.0% | 38.6% | 33.3% | **32.2%** | 28.4% | 38.5% | 23.5% | 36.0% | **18.8%** | 12.0% | 15.0% | 51.9% | 5.8% | 37.2% | 34.8% | 53.8%* | 29.1% | 10.8% | 23.9%* |
| **Community-based clinic** | Maybe | **13.4%** | 24.6%^ | 10.1% | 5.3%§ | **24.6%** | 29.7% | 27.0% | 24.6% | 9.8% | **10.1%** | 13.7% | 11.5% | 5.9% | 4.0% | **5.3%** | 5.0% | 9.0% | 1.9% | 1.9% | 20.0% | 28.7% | 2.6% | 11.2% | 6.7% | 4.3% |
| **A pharmacy next to a clinic** | Yes | **54.3%** | 38.8%§ | 48.2% | 76.3%^ | **38.8%** | 32.7% | 44.0% | 40.4% | 39.2% | **48.2%** | 44.1% | 45.2% | 58.8% | 52.0% | **76.3%** | 79.0% | 81.0% | 59.6% | 78.8% | 43.4% | 34.8% | 38.5% | 49.6% | 85.8%* | 70.1% |
| **A pharmacy next to a clinic** | No | **31.2%** | 40.5% | 34.9% | 18.1%§ | **40.5%** | 40.6% | 36.0% | 42.1% | 47.1% | **34.9%** | 38.2% | 36.5% | 23.5% | 36.0% | **18.1%** | 11.0% | 13.0% | 38.5% | 21.2% | 39.3% | 41.5% | 46.2% | 33.2% | 9.2% | 23.9%* |
| **A pharmacy next to a clinic** | Maybe | **14.5%** | 20.7% | 16.9% | 5.6%§ | **20.7%** | 26.7% | 20.0% | 17.5% | 13.7% | **16.9%** | 17.6% | 18.3% | 17.6% | 12.0% | **5.6%** | 10.0% | 6.0% | 1.9% | - | 17.2% | 23.8% | 15.4% | 17.2% | 5.0% | 6.0% |
| **Mobile clinic** | Yes | **39.7%** | 33.3% | 26.4% | 59.5%^ | **33.3%** | 19.8% | 50.0% | 36.8% | 23.5% | **26.4%** | 25.5% | 20.2% | 39.2% | 28.0% | **59.5%** | 60.0% | 65.0% | 50.0% | 57.7% | 32.4% | 34.1% | 25.6% | 26.5% | 73.3%* | 50.5% |
| **Mobile clinic** | No | **42.4%** | 43.0% | 54.4% | 29.6%§ | **43.0%** | 47.5% | 29.0% | 49.1% | 54.9% | **54.4%** | 57.8% | 60.6% | 29.4% | 60.0% | **29.6%** | 24.0% | 23.0% | 46.2% | 36.5% | 45.5% | 40.9% | 59.0% | 53.7% | 17.5% | 37.5%* |
| **Mobile clinic** | Maybe | **17.9%** | 23.6% | 19.2% | 10.9%§ | **23.6%** | 32.7% | 21.0% | 14.0% | 21.6% | **19.2%** | 16.7% | 19.2% | 31.4% | 12.0% | **10.9%** | 16.0% | 12.0% | 3.8% | 5.8% | 22.1% | 25.0% | 15.4% | 19.8% | 9.2% | 12.0% |
| **Outreach organisation** | Yes | **39.5%** | 29.4% | 30.6% | 58.6%^ | **29.4%** | 19.8% | 43.0% | 28.1% | 23.5% | **30.6%** | 14.7% | 16.3% | 74.5% | 48.0% | **58.6%** | 48.0% | 65.0% | 65.4% | 59.6% | 29.0% | 29.9% | 23.1% | 31.7% | 69.2%* | 51.6% |
| **Outreach organisation** | No | **38.9%** | 41.4% | 50.5%^ | 24.7%§ | **41.4%** | 45.5% | 32.0% | 45.6% | 47.1% | **50.5%** | 58.8% | 59.6% | 21.6% | 44.0% | **24.7%** | 23.0% | 20.0% | 30.8% | 30.8% | 40.7% | 42.1% | 51.3% | 50.4% | 14.2% | 31.5%* |
| **Outreach organisation** | Maybe | **21.6%** | 29.1%^ | 18.9% | 16.8% | **29.1%** | 34.7% | 25.0% | 26.3% | 29.4% | **18.9%** | 26.5% | 24.0% | 3.9% | 8.0% | **16.8%** | 29.0% | 15.0% | 3.8% | 9.6% | 30.3% | 28.0% | 25.6% | 17.9% | 16.7% | 16.8% |
| **Drop-in centre clinic** | Yes | **35.3%** | 22.7% | 24.1% | 59.5%^ | **22.7%** | 18.8% | 28.0% | 22.8% | 19.6% | **24.1%** | 15.7% | 17.3% | 45.1% | 34.0% | **59.5%** | 60.0% | 64.0% | 46.2% | 63.5% | 23.4% | 22.0% | 15.4% | 25.4% | 73.3%* | 50.0% |
| **Drop-in centre clinic** | No | **43.3%** | 48.2% | 53.1% | 28.3%§ | **48.2%** | 48.5% | 45.0% | 52.6% | 49.0% | **53.1%** | 56.9% | 61.5% | 27.5% | 54.0% | **28.3%** | 21.0% | 22.0% | 51.9% | 30.8% | 49.0% | 47.6% | 53.8% | 53.0% | 16.7% | 35.9%* |
| **Drop-in centre clinic** | Maybe | **21.4%** | 29.1% | 22.8% | 12.2%§ | **29.1%** | 32.7% | 27.0% | 24.6% | 31.4% | **22.8%** | 27.5% | 21.2% | 27.5% | 12.0% | **12.2%** | 19.0% | 14.0% | 1.9% | 5.8% | 27.6% | 30.5% | 30.8% | 21.6% | 9.2% | 14.1% |
| **A&E units at a hospital** | Yes | **33.6%** | 36.6% | 27.0%§ | 37.2% | **36.6%** | 53.5% | 40.0% | 15.8% | 19.6% | **27.0%** | 33.3% | 32.7% | 25.5% | 4.0% | **37.2%** | 36.0% | 49.0% | 17.3% | 36.5% | 35.2% | 37.8% | 38.5% | 25.4% | 46.7%* | 31.0% |
| **A&E units at a hospital** | No | **48.5%** | 47.6% | 55.0% | 42.8% | **47.6%** | 36.6% | 42.0% | 61.4% | 64.7% | **55.0%** | 54.9% | 52.9% | 43.1% | 72.0% | **42.8%** | 34.0% | 31.0% | 73.1% | 51.9% | 49.7% | 45.7% | 53.8% | 55.2% | 31.7% | 50.0% |
| **A&E units at a hospital** | Maybe | **17.9%** | 15.9% | 17.9% | 20.1% | **15.9%** | 9.9% | 18.0% | 22.8% | 15.7% | **17.9%** | 11.8% | 14.4% | 31.4% | 24.0% | **20.1%** | 30.0% | 20.0% | 9.6% | 11.5% | 15.2% | 16.5% | 7.7% | 19.4% | 21.7% | 19.0% |
| **A website** | Yes | **29.6%** | 22.0%§ | 31.3% | 35.5% | **22.0%** | 28.7% | 24.0% | 12.3% | 15.7% | **31.3%** | 25.5% | 28.8% | 43.1% | 36.0% | **35.5%** | 31.0% | 39.0% | 38.5% | 34.6% | 17.9% | 25.6% | 20.5% | 32.8% | 40.0% | 32.6% |
| **A website** | No | **52.2%** | 55.3% | 57.0% | 44.1%§ | **55.3%** | 55.4% | 54.0% | 56.1% | 56.9% | **57.0%** | 61.8% | 61.5% | 43.1% | 52.0% | **44.1%** | 38.0% | 46.0% | 51.9% | 44.2% | 62.1%* | 49.4% | 64.1% | 56.0% | 34.2% | 50.5% |
| **A website** | Maybe | **18.3%** | 22.7% | 11.7%§ | 20.4% | **22.7%** | 15.8% | 22.0% | 31.6% | 27.5% | **11.7%** | 12.7% | 9.6% | 13.7% | 12.0% | **20.4%** | 31.0% | 15.0% | 9.6% | 21.2% | 20.0% | 25.0% | 15.4% | 11.2% | 25.8% | 16.8% |
| **Peer provider** | Yes | **27.4%** | 24.3% | 19.2% | 38.8%^ | **24.3%** | 19.8% | 26.0% | 21.1% | 33.3% | **19.2%** | 7.8% | 7.7% | 47.1% | 38.0% | **38.8%** | 47.0% | 38.0% | 11.5% | 51.9% | 29.0% | 20.1% | 10.3% | 20.5% | 40.0% | 38.0% |
| **Peer provider** | No | **52.7%** | 51.5% | 57.0% | 49.7% | **51.5%** | 52.5% | 51.0% | 49.1% | 52.9% | **57.0%** | 62.7% | 63.5% | 33.3% | 56.0% | **49.7%** | 39.0% | 49.0% | 76.9% | 44.2% | 48.3% | 54.3% | 61.5% | 56.3% | 51.7% | 48.4% |
| **Peer provider** | Maybe | **19.9%** | 24.3% | 23.8% | 11.5%§ | **24.3%** | 27.7% | 23.0% | 29.8% | 13.7% | **23.8%** | 29.4% | 28.8% | 19.6% | 6.0% | **11.5%** | 14.0% | 13.0% | 11.5% | 3.8% | 22.8% | 25.6% | 28.2% | 23.1% | 8.3% | 13.6% |
| **A vending machine** | Yes | **17.8%** | 17.5% | 13.7% | 22.4% | **17.5%** | 10.9% | 32.0% | 14.0% | 5.9% | **13.7%** | 7.8% | 6.7% | 27.5% | 26.0% | **22.4%** | 21.0% | 24.0% | 26.9% | 17.3% | 13.8% | 20.7% | 5.1% | 14.9% | 25.0% | 20.7% |
| **A vending machine** | No | **64.2%** | 62.8% | 70.0% | 59.9% | **62.8%** | 70.3% | 51.0% | 64.9% | 68.6% | **70.0%** | 76.5% | 72.1% | 60.8% | 62.0% | **59.9%** | 55.0% | 57.0% | 63.5% | 71.2% | 69.7%* | 56.7% | 71.8% | 69.8% | 57.5% | 61.4% |
| **A vending machine** | Maybe | **17.9%** | 19.7% | 16.3% | 17.8% | **19.7%** | 18.8% | 17.0% | 21.1% | 25.5% | **16.3%** | 15.7% | 21.2% | 11.8% | 12.0% | **17.8%** | 24.0% | 19.0% | 9.6% | 11.5% | 16.6% | 22.6% | 23.1% | 15.3% | 17.5% | 17.9% |
| **Police station** | Yes | **9.1%** | 7.1% | 3.6% | 16.8%^ | **7.1%** | 5.0% | 16.0% | 1.8% | - | **3.6%** | 1.0% | 1.0% | 15.7% | 2.0% | **16.8%** | 25.0% | 20.0% | 1.9% | 9.6% | 7.6% | 6.7% | - | 4.1% | 27.5%* | 9.8% |
| **Police station** | No | **79.1%** | 82.2% | 84.4% | 70.7%§ | **82.2%** | 82.2% | 73.0% | 91.2% | 90.2% | **84.4%** | 93.1% | 87.5% | 60.8% | 84.0% | **70.7%** | 60.0% | 66.0% | 92.3% | 78.8% | 82.8% | 81.7% | 97.4%* | 82.5% | 63.3% | 75.5%* |
| **Police station** | Maybe | **11.7%** | 10.7% | 12.1% | 12.5% | **10.7%** | 12.9% | 11.0% | 7.0% | 9.8% | **12.1%** | 5.9% | 11.5% | 23.5% | 14.0% | **12.5%** | 15.0% | 14.0% | 5.8% | 11.5% | 9.7% | 11.6% | 2.6% | 13.4% | 9.2% | 14.7% |
|  |  |  |  |  |  |  |  |  |  |  |  |  |  |  |  |  |  |  |  |  |  |  |  |  |  |  |
| Abbreviations: HIV, Human Immunodeficiency Disease, PEP, Post-Exposure Prophylaxis; YW, Young Women. NB. Key Population includes Female Sex Workers and Men who have Sex with Men. § Significantly lower than the other 2 countries, ^ Significantly higher than the other 2 countries. * Statistically higher than PEP/Non-PEP sample. † Low base size. | | | | | | | | | | | | | | | | | | | | | | | | | | |
