# Supplementary material for: Qualitative and quantitative research on preferences and perceptions regarding HIV post-exposure prophylaxis among young women, men, female sex workers, members of the LGBTQ + community and people who inject drugs in Kenya, Nigeria and Zimbabwe
Source: Front Reprod Health. 2025 Oct 9;7:1606013. doi: 10.3389/frph.2025.1606013 (PMC12546014; doi:10.3389/frph.2025.1606013)
Supplement: Supplementary Table S4 — (PEP Experienced Users only): Recall of specific services offered within the PEP pathway. [file Table2.docx]

**Table S3: Reported demographics: Relationship status, number of children, healthcare facility most often used, healthcare insurance, work status, highest level of schooling achieved, religion and regional split.**

| **DEMOGRAPHICS** | **TOTAL (n=920)** | **Kenya (n=309)** | **Nigeria (n=307)** | **Zimbabwe (n=304)** | **TOTAL (n=309)** | **YW (n=101)** | **Men (n=100)** | **MSM (n=57)** | **FSW (n=51)** | **TOTAL (n=307)** | **YW (n=102)** | **Men (n=104)** | **MSM (n=51)** | **FSW (n=50†)** | **TOTAL (n=304)** | **YW (n=100)** | **Men (n=100)** | **MSM (n=52)** | **FSW (n=52)** |
| --- | --- | --- | --- | --- | --- | --- | --- | --- | --- | --- | --- | --- | --- | --- | --- | --- | --- | --- | --- |
| **Relationship Status** | **TOTAL** | **COUNTRY** | | | **KENYA** | | | | | **NIGERIA** | | | | | **ZIMBABWE** | | | | |
| Married, living together without times of separation | ***12.3%*** | *12.0%* | *14.3%* | *10.5%* | ***12.0%*** | *10.9%* | *24.0%* | *3.5%* | *-* | ***14.3%*** | *13.7%* | *22.1%* | *11.8%* | *2.0%* | ***10.5%*** | *9.0%* | *23.0%* | *-* | *-* |
| Married, living together, but with periods of separation of 2 or more weeks from time to time | ***4.2%*** | *5.5%* | *2.6%* | *4.6%* | ***5.5%*** | *3.0%* | *10.0%* | *3.5%* | *3.9%* | ***2.6%*** | *1.0%* | *5.8%* | *2.0%* | *-* | ***4.6%*** | *5.0%* | *9.0%* | *-* | *-* |
| Married, not living together | ***3.5%*** | *2.9%* | *4.6%* | *3.0%* | ***2.9%*** | *2.0%* | *4.0%* | *1.8%* | *3.9%* | ***4.6%*** | *2.0%* | *7.7%* | *3.9%* | *4.0%* | ***3.0%*** | *2.0%* | *3.0%* | *3.8%* | *3.8%* |
| Living together as partners, not married | ***7.8%*** | *11.7%* | *3.9%§* | *7.9%* | ***11.7%*** | *12.9%* | *11.0%* | *15.8%* | *5.9%* | ***3.9%*** | *-* | *7.7%* | *3.9%* | *4.0%* | ***7.9%*** | *9.0%* | *9.0%* | *9.6%* | *1.9%* |
| Single - with one partner | ***32.9%*** | *30.1%* | *39.1%^* | *29.6%* | ***30.1%*** | *50.5%* | *21.0%* | *26.3%* | *0.1* | ***39.1%*** | *63.7%* | *28.8%* | *29.4%* | *0.2* | ***29.6%*** | *46.0%* | *24.0%* | *36.5%* | *0.0* |
| Single - with more than one partner | ***23.3%*** | *24.9%* | *14.0%§* | *30.9%* | ***24.9%*** | *11.9%* | *22.0%* | *26.3%* | *54.9%* | ***14.0%*** | *4.9%* | *17.3%* | *15.7%* | *24.0%* | ***30.9%*** | *12.0%* | *18.0%* | *38.5%* | *84.6%* |
| Single - no partner | ***16.0%*** | *12.9%* | *21.5%^* | *13.5%* | ***12.9%*** | *8.9%* | *8.0%* | *22.8%* | *0.2* | ***21.5%*** | *14.7%* | *10.6%* | *33.3%* | *0.5* | ***13.5%*** | *17.0%* | *14.0%* | *11.5%* | *0.1* |
| **No. children** | **TOTAL** | **COUNTRY** | | | **KENYA** | | | | | **NIGERIA** | | | | | **ZIMBABWE** | | | | |
| Mean | **0.7** | 0.7 | 0.7 | 0.7 | **0.7** | 0.6 | 0.9 | 0.3 | *80.0%* | **0.7** | 0.3 | 0.9 | 0.8 | *100.0%* | **0.7** | 0.5 | 1.2 | 0.1 | *100.0%* |
| 0 | ***59.7%*** | *56.6%* | *64.8%* | *57.6%* | ***56.6%*** | *52.5%* | *49.0%* | *82.5%* | *51.0%* | ***64.8%*** | *79.4%* | *51.9%* | *68.6%* | *58.0%* | ***57.6%*** | *61.0%* | *45.0%* | *92.3%* | *40.4%* |
| 1 | ***20.4%*** | *26.2%* | *14.3%§* | *20.7%* | ***26.2%*** | *35.6%* | *26.0%* | *8.8%* | *27.5%* | ***14.3%*** | *16.7%* | *16.3%* | *5.9%* | *14.0%* | ***20.7%*** | *31.0%* | *17.0%* | *3.8%* | *25.0%* |
| 2 | ***11.8%*** | *11.7%* | *10.1%* | *13.8%* | ***11.7%*** | *9.9%* | *16.0%* | *7.0%* | *0.1* | ***10.1%*** | *2.9%* | *19.2%* | *7.8%* | *0.1* | ***13.8%*** | *6.0%* | *19.0%* | *1.9%* | *0.3* |
| 3 | ***6.5%*** | *4.5%* | *8.5%* | *6.6%* | ***4.5%*** | *2.0%* | *7.0%* | *1.8%* | *7.8%* | ***8.5%*** | *1.0%* | *10.6%* | *11.8%* | *16.0%* | ***6.6%*** | *2.0%* | *15.0%* | *1.9%* | *3.8%* |
| 4 | ***1.4%*** | *1.0%* | *2.0%* | *1.3%* | ***1.0%*** | *-* | *2.0%* | *-* | *0.0* | ***2.0%*** | *-* | *1.9%* | *5.9%* | *0.0* | ***1.3%*** | *-* | *4.0%* | *-* | *-* |
| 5 | ***0.1%*** | *-* | *0.3%* | *-* | ***-*** | *-* | *-* | *-* | *-* | ***0.0*** | *-* | *-* | *-* | *2.0%* | ***-*** | *-* | *-* | *-* | *-* |
| **Healthcare Facility most often used** | **TOTAL** | **COUNTRY** | | | **KENYA** | | | | | **NIGERIA** | | | | | **ZIMBABWE** | | | | |
| A public health clinic/ centre | ***24.1%*** | *15.2%* | *20.8%* | *36.5%^* | ***15.2%*** | *18.8%* | *11.0%* | *12.3%* | *19.6%* | ***20.8%*** | *20.6%* | *13.5%* | *25.5%* | *32.0%* | ***36.5%*** | *44.0%* | *36.0%* | *1.9%* | *57.7%* |
| A public / government hospital | ***39.2%*** | *44.7%* | *35.5%* | *37.5%* | ***44.7%*** | *50.5%* | *46.0%* | *33.3%* | *43.1%* | ***35.5%*** | *49.0%* | *39.4%* | *19.6%* | *16.0%* | ***37.5%*** | *40.0%* | *41.0%* | *25.0%* | *38.5%* |
| Another public health facility | ***0.1%*** | *-* | *-* | *0.3%* | ***-*** | *-* | *-* | *-* | *-* | ***-*** | *-* | *-* | *-* | *-* | ***0.0*** | *0.0* | *-* | *-* | *-* |
| A private health clinic / centre | ***12.3%*** | *17.5%* | *3.9%§* | *15.5%* | ***17.5%*** | *12.9%* | *17.0%* | *29.8%* | *13.7%* | ***3.9%*** | *2.0%* | *2.9%* | *2.0%* | *12.0%* | ***15.5%*** | *8.0%* | *11.0%* | *53.8%* | *-* |
| A private hospital | ***19.3%*** | *19.1%* | *30.0%^* | *8.9%* | ***19.1%*** | *16.8%* | *23.0%* | *15.8%* | *0.2* | ***30.0%*** | *22.5%* | *33.7%* | *47.1%* | *0.2* | ***8.9%*** | *7.0%* | *9.0%* | *17.3%* | *0.0* |
| Another private health facility | ***-*** | *-* | *-* | *-* | ***-*** | *-* | *-* | *-* | *-* | ***-*** | *-* | *-* | *-* | *-* | ***-*** | *-* | *-* | *-* | *-* |
| A pharmacy | ***4.9%*** | *3.6%* | *9.8%^* | *1.3%* | ***3.6%*** | *1.0%* | *3.0%* | *8.8%* | *3.9%* | ***9.8%*** | *5.9%* | *10.6%* | *5.9%* | *20.0%* | ***1.3%*** | *-* | *3.0%* | *1.9%* | *-* |
| **Healthcare Medical Insurance** | **TOTAL** | **COUNTRY** | | | **KENYA** | | | | | **NIGERIA** | | | | | **ZIMBABWE** | | | | |
| Yes | ***35.1%*** | *52.1%^* | *20.5%* | *32.6%* | ***52.1%*** | *38.6%* | *67.0%* | *56.1%* | *45.1%* | ***20.5%*** | *5.9%* | *18.3%* | *43.1%* | *32.0%* | ***32.6%*** | *34.0%* | *29.0%* | *55.8%* | *13.5%* |
| No | ***64.9%*** | *47.9%* | *79.5%^* | *67.4%* | ***47.9%*** | *61.4%* | *33.0%* | *43.9%* | *0.5* | ***79.5%*** | *94.1%* | *81.7%* | *56.9%* | *0.7* | ***67.4%*** | *66.0%* | *71.0%* | *44.2%* | *0.9* |
| **Working Status** | **TOTAL** | **COUNTRY** | | | **KENYA** | | | | | **NIGERIA** | | | | | **ZIMBABWE** | | | | |
| Full-time Employed | ***21.7%*** | *21.0%* | *20.2%* | *24.0%* | ***21.0%*** | *14.9%* | *29.0%* | *36.8%* | *-* | ***20.2%*** | *12.7%* | *35.6%* | *21.6%* | *2.0%* | ***24.0%*** | *15.0%* | *41.0%* | *32.7%* | *-* |
| Part-time Employed | ***20.9%*** | *25.6%* | *15.3%§* | *21.7%* | ***25.6%*** | *20.8%* | *29.0%* | *22.8%* | *31.4%* | ***15.3%*** | *15.7%* | *16.3%* | *17.6%* | *10.0%* | ***21.7%*** | *11.0%* | *21.0%* | *28.8%* | *36.5%* |
| Unemployed (but seeking work) | ***14.1%*** | *19.1%* | *4.6%§* | *18.8%* | ***19.1%*** | *22.8%* | *13.0%* | *8.8%* | *35.3%* | ***4.6%*** | *5.9%* | *1.9%* | *5.9%* | *6.0%* | ***18.8%*** | *22.0%* | *14.0%* | *15.4%* | *25.0%* |
| Unemployed (and NOT seeking work); homemaker | ***2.5%*** | *1.0%* | *1.6%* | *4.9%^* | ***1.0%*** | *2.0%* | *-* | *-* | *2.0%* | ***1.6%*** | *1.0%* | *-* | *0.0* | *6.0%* | ***4.9%*** | *10.0%* | *0.0* | *-* | *7.7%* |
| Student | ***12.1%*** | *7.1%* | *11.4%* | *17.8%^* | ***7.1%*** | *8.9%* | *6.0%* | *7.0%* | *5.9%* | ***11.4%*** | *27.5%* | *1.9%* | *3.9%* | *6.0%* | ***17.8%*** | *33.0%* | *13.0%* | *15.4%* | *-* |
| Self-employed | ***28.7%*** | *26.2%* | *46.9%^* | *12.8%* | ***26.2%*** | *30.7%* | *23.0%* | *24.6%* | *0.3* | ***46.9%*** | *37.3%* | *44.2%* | *49.0%* | *0.7* | ***12.8%*** | *9.0%* | *10.0%* | *7.7%* | *0.3* |
| **Highest Level of Schooling achieved** | **TOTAL** | **COUNTRY** | | | **KENYA** | | | | | **NIGERIA** | | | | | **ZIMBABWE** | | | | |
| No formal schooling | ***0.9%*** | *0.3%* | *1.3%* | *1.0%* | ***0.3%*** | *1.0%* | *-* | *-* | *-* | ***1.3%*** | *-* | *-* | *0.0* | *4.0%* | ***1.0%*** | *-* | *-* | *-* | *5.8%* |
| Grade 1 to 3 / KG 1-3 | ***-*** | *-* | *-* | *-* | ***-*** | *-* | *-* | *-* | *-* | ***-*** | *-* | *-* | *-* | *-* | ***-*** | *-* | *-* | *-* | *-* |
| Grade 4 to 7 / Lower primary | ***2.0%*** | *1.6%* | *0.7%* | *3.6%* | ***1.6%*** | *2.0%* | *1.0%* | *3.5%* | *-* | ***0.7%*** | *-* | *-* | *-* | *4.0%* | ***3.6%*** | *2.0%* | *2.0%* | *1.9%* | *11.5%* |
| Grade 8-11 / Upper primary | ***8.2%*** | *8.4%* | *4.9%* | *11.2%* | ***8.4%*** | *11.9%* | *6.0%* | *5.3%* | *9.8%* | ***4.9%*** | *1.0%* | *2.9%* | *5.9%* | *16.0%* | ***11.2%*** | *14.0%* | *8.0%* | *-* | *23.1%* |
| Grade 12 / High school | ***39.8%*** | *35.6%* | *41.4%* | *42.4%* | ***35.6%*** | *49.5%* | *22.0%* | *17.5%* | *54.9%* | ***41.4%*** | *48.0%* | *27.9%* | *35.3%* | *62.0%* | ***42.4%*** | *46.0%* | *40.0%* | *34.6%* | *48.1%* |
| Diploma/certificates/undergraduate | ***49.2%*** | *54.0%* | *51.8%* | *41.8%* | ***54.0%*** | *35.6%* | *71.0%* | *73.7%* | *0.4* | ***51.8%*** | *51.0%* | *69.2%* | *54.9%* | *0.1* | ***41.8%*** | *38.0%* | *50.0%* | *63.5%* | *0.1* |
| **Religion** | **TOTAL** | **COUNTRY** | | | **KENYA** | | | | | **NIGERIA** | | | | | **ZIMBABWE** | | | | |
| Christian - Traditional (Anglican, Baptist, Catholic, Lutheran, Methodist, Presbyterian)/Christian pentecoastal | ***66.2%*** | *78.6%* | *46.9%* | *73.0%* | ***78.6%*** | *77.2%* | *76.0%* | *84.2%* | *80.4%* | ***46.9%*** | *42.2%* | *50.0%* | *41.2%* | *56.0%* | ***73.0%*** | *73.0%* | *79.0%* | *71.2%* | *63.5%* |
| Christian - African/Zionist Churches (ZCC and independent African churches) | ***7.7%*** | *5.2%* | *5.9%* | *12.2%^* | ***5.2%*** | *5.0%* | *4.0%* | *5.3%* | *7.8%* | ***5.9%*** | *5.9%* | *4.8%* | *7.8%* | *6.0%* | ***12.2%*** | *20.0%* | *12.0%* | *3.8%* | *5.8%* |
| Charismatic/Born Again/Rhema | ***8.9%*** | *1.0%* | *25.1%^* | *0.7%* | ***1.0%*** | *2.0%* | *1.0%* | *-* | *-* | ***25.1%*** | *32.4%* | *22.1%* | *23.5%* | *18.0%* | ***0.7%*** | *-* | *1.0%* | *1.9%* | *-* |
| Muslim/Islam | ***9.9%*** | *11.7%* | *17.6%^* | *0.3%* | ***11.7%*** | *14.9%* | *17.0%* | *3.5%* | *3.9%* | ***17.6%*** | *19.6%* | *23.1%* | *11.8%* | *8.0%* | ***0.3%*** | *1.0%* | *-* | *-* | *-* |
| Agnostic or Atheist (no religion/belief in God) | ***2.1%*** | *1.9%* | *1.6%* | *2.6%* | ***1.9%*** | *-* | *-* | *0.1* | *3.9%* | ***1.6%*** | *-* | *-* | *0.1* | *4.0%* | ***2.6%*** | *0.0* | *0.0* | *0.1* | *5.8%* |
| Animist/ancestor worship/traditional tribal belief | ***0.8%*** | *0.6%* | *0.7%* | *1.0%* | ***0.6%*** | *-* | *1.0%* | *-* | *0.0* | ***0.7%*** | *-* | *-* | *2.0%* | *0.0* | ***1.0%*** | *-* | *1.0%* | *3.8%* | *-* |
| Do not want to say | ***1.8%*** | *0.3%§* | *2.3%* | *3.0%* | ***0.3%*** | *1.0%* | *-* | *-* | *-* | ***2.3%*** | *-* | *-* | *0.1* | *6.0%* | ***3.0%*** | *1.0%* | *0.0* | *0.0* | *5.8%* |
| Not stated | ***2.6%*** | *0.6%* | *-* | *7.2%^* | ***0.6%*** | *-* | *1.0%* | *-* | *0.0* | ***-*** | *-* | *-* | *-* | *-* | ***7.2%*** | *0.0* | *3.0%* | *9.6%* | *0.2* |
| **KENYA REGIONS** | **TOTAL** | **COUNTRY** | | | **KENYA** | | | | | **NIGERIA** | | | | | **ZIMBABWE** | | | | |
| **Nairobi (Net)** | **-** | **67%** | **-** | **-** | **67%** | **50%** | **48%** | **100%** | **100%** | **-** | **-** | **-** | **-** | **-** | **-** | **-** | **-** | **-** | **-** |
| *Dagoretti* | **-** | 7% | - | - | 7% | 5% | 5% | 7% | 18% | - | - | - | - | - | - | - | - | - | - |
| *Embakasi* | **-** | 10% | - | - | 10% | 12% | 12% | 12% | 2% | - | - | - | - | - | - | - | - | - | - |
| *Kamukunji* | **-** | 3% | - | - | 3% | 3% | 3% | 4% | 2% | - | - | - | - | - | - | - | - | - | - |
| *Kasarani* | **-** | 11% | - | - | 11% | 9% | 9% | 14% | 14% | - | - | - | - | - | - | - | - | - | - |
| *Kibra* | **-** | 4% | - | - | 4% | 2% | 2% | 11% | 4% | - | - | - | - | - | - | - | - | - | - |
| *Lang'ata* | **-** | 6% | - | - | 6% | 2% | 2% | 5% | 22% | - | - | - | - | - | - | - | - | - | - |
| *Makadara* | **-** | 3% | - | - | 3% | 2% | 2% | 2% | 6% | - | - | - | - | - | - | - | - | - | - |
| *Mathare* | **-** | 3% | - | - | 3% | 2% | 1% | 9% | 2% | - | - | - | - | - | - | - | - | - | - |
| *Njiru* | **-** | 6% | - | - | 6% | 7% | 6% | 5% | 2% | - | - | - | - | - | - | - | - | - | - |
| *Starehe* | **-** | 3% | - | - | 3% | 3% | 3% | 2% | 6% | - | - | - | - | - | - | - | - | - | - |
| *Westlands* | **-** | 12% | - | - | 12% | 4% | 3% | 30% | 24% | - | - | - | - | - | - | - | - | - | - |
| **Mombasa (Net)** | **-** | **33%** | **-** | **-** | **33%** | **50%** | **52%** | **0%** | **0%** | **-** | **-** | **-** | **-** | **-** | **-** | **-** | **-** | **-** | **-** |
| *Changamwe* | **-** | 3% | - | - | 3% | 5% | 5% | 0% | 0% | - | - | - | - | - | - | - | - | - | - |
| *Jomvu* | **-** | 5% | - | - | 5% | 6% | 8% | 0% | 0% | - | - | - | - | - | - | - | - | - | - |
| *Kisauni* | **-** | 8% | - | - | 8% | 12% | 13% | 0% | 0% | - | - | - | - | - | - | - | - | - | - |
| *Kikoni* | **-** | 7% | - | - | 7% | 11% | 11% | 0% | 0% | - | - | - | - | - | - | - | - | - | - |
| *Mvita* | **-** | 4% | - | - | 4% | 7% | 6% | 0% | 0% | - | - | - | - | - | - | - | - | - | - |
| *Nyali* | **-** | 6% | - | - | 6% | 9% | 9% | 0% | 0% | - | - | - | - | - | - | - | - | - | - |
| **NIGERIA REGIONS** | **TOTAL** | **COUNTRY** | | | **KENYA** | | | | | **NIGERIA** | | | | | **ZIMBABWE** | | | | |
| **Lagos (Net)** | **-** | **-** | **69%** | **-** | **-** | **-** | **-** | **-** | **-** | **69%** | **55%** | **54%** | **100%** | **100%** | **-** | **-** | **-** | **-** | **-** |
| *Agege* | - | - | 3% | - | - | - | - | - | - | 3% | 5% | 4% | 0% | 2% | - | - | - | - | - |
| *Ajeromi-Ifelodun* | - | - | 3% | - | - | - | - | - | - | 3% | 5% | 4% | 0% | 0% | - | - | - | - | - |
| *Alimosho* | - | - | 5% | - | - | - | - | - | - | 5% | 8% | 7% | 0% | 0% | - | - | - | - | - |
| *Amuwo-Odofin* | - | - | 2% | - | - | - | - | - | - | 2% | 3% | 2% | 0% | 0% | - | - | - | - | - |
| *Apapa* | - | - | 1% | - | - | - | - | - | - | 1% | 2% | 2% | 0% | 0% | - | - | - | - | - |
| *Badagry* | - | - | 1% | - | - | - | - | - | - | 1% | 1% | 1% | 0% | 0% | - | - | - | - | - |
| *Epe* | - | - | 1% | - | - | - | - | - | - | 1% | 1% | 1% | 0% | 0% | - | - | - | - | - |
| *Eti-Osa* | - | - | 2% | - | - | - | - | - | - | 2% | 2% | 3% | 0% | 0% | - | - | - | - | - |
| *Ibeju-Lekki* | - | - | 1% | - | - | - | - | - | - | 1% | 1% | 1% | 0% | 0% | - | - | - | - | - |
| *Ifako-Ijaiye* | - | - | 1% | - | - | - | - | - | - | 1% | 2% | 2% | 0% | 0% | - | - | - | - | - |
| *Ikeja* | - | - | 3% | - | - | - | - | - | - | 3% | 2% | 3% | 4% | 4% | - | - | - | - | - |
| *Ikorodu* | - | - | 2% | - | - | - | - | - | - | 2% | 3% | 3% | 0% | 0% | - | - | - | - | - |
| *Kosofe* | - | - | 3% | - | - | - | - | - | - | 3% | 4% | 4% | 0% | 4% | - | - | - | - | - |
| *Lagos Island* | - | - | 2% | - | - | - | - | - | - | 2% | 1% | 1% | 8% | 2% | - | - | - | - | - |
| *Lagos Mainland* | - | - | 29% | - | - | - | - | - | - | 29% | 2% | 3% | 88% | 80% | - | - | - | - | - |
| *Mushin* | - | - | 2% | - | - | - | - | - | - | 2% | 3% | 3% | 0% | 0% | - | - | - | - | - |
| *Ojo* | - | - | 2% | - | - | - | - | - | - | 2% | 3% | 3% | 0% | 2% | - | - | - | - | - |
| *Oshodi-Isolo* | - | - | 2% | - | - | - | - | - | - | 2% | 3% | 4% | 0% | 0% | - | - | - | - | - |
| *Shomolu* | - | - | 2% | - | - | - | - | - | - | 2% | 2% | 2% | 0% | 4% | - | - | - | - | - |
| *Surulere* | - | - | 2% | - | - | - | - | - | - | 2% | 3% | 3% | 0% | 2% | - | - | - | - | - |
| **Abuja (Net)** | **-** | **-** | **31%** | **-** | **-** | **-** | **-** | **-** | **-** | **31%** | **45%** | **46%** | **0%** | **0%** | **-** | **-** | **-** | **-** | **-** |
| *Abaji* | - | - | 1% | - | - | - | - | - | - | 1% | 1% | 2% | 0% | 0% | - | - | - | - | - |
| *Abuja Municipal Area Council* | - | - | 18% | - | - | - | - | - | - | 18% | 26% | 27% | 0% | 0% | - | - | - | - | - |
| *Bwari* | - | - | 5% | - | - | - | - | - | - | 5% | 7% | 8% | 0% | 0% | - | - | - | - | - |
| *Gwagwalada* | - | - | 4% | - | - | - | - | - | - | 4% | 5% | 6% | 0% | 0% | - | - | - | - | - |
| *Kuje* | - | - | 2% | - | - | - | - | - | - | 2% | 3% | 2% | 0% | 0% | - | - | - | - | - |
| *Kwali* | - | - | 2% | - | - | - | - | - | - | 2% | 3% | 2% | 0% | 0% | - | - | - | - | - |
| **ZIMBABWE REGIONS** | **TOTAL** | **COUNTRY** | | | **KENYA** | | | | | **NIGERIA** | | | | | **ZIMBABWE** | | | | |
| **Harare (Net)** | **-** | **-** | **-** | **67%** | **-** | **-** | **-** | **-** | **-** | **-** | **-** | **-** | **-** | **-** | **67%** | **51%** | **50%** | **100%** | **100%** |
| *Ward 01* | - | - | - | 20% | - | - | - | - | - | - | - | - | - | - | 20% | 19% | 20% | 13% | 29% |
| *Ward 15* | - | - | - | 5% | - | - | - | - | - | - | - | - | - | - | 5% | 4% | 3% | 12% | 6% |
| *Ward 23* | - | - | - | 12% | - | - | - | - | - | - | - | - | - | - | 12% | 4% | 3% | 40% | 17% |
| *Ward 37* | - | - | - | 5% | - | - | - | - | - | - | - | - | - | - | 5% | 4% | 4% | 6% | 8% |
| *Ward 43* | - | - | - | 3% | - | - | - | - | - | - | - | - | - | - | 3% | 5% | 5% | 0% | 0% |
| *Ward 30* | - | - | - | 3% | - | - | - | - | - | - | - | - | - | - | 3% | 3% | 3% | 6% | 0% |
| *Ward 22* | - | - | - | 3% | - | - | - | - | - | - | - | - | - | - | 3% | 4% | 2% | 0% | 6% |
| *Ward 09* | - | - | - | 9% | - | - | - | - | - | - | - | - | - | - | 9% | 3% | 3% | 23% | 17% |
| *Ward 33* | - | - | - | 5% | - | - | - | - | - | - | - | - | - | - | 5% | 3% | 3% | 0% | 17% |
| *Ward 40* | - | - | - | 3% | - | - | - | - | - | - | - | - | - | - | 3% | 4% | 4% | 0% | 0% |
| **Bulawayo (Net)** | **-** | **-** | **-** | **33%** | **-** | **-** | **-** | **-** | **-** | **-** | **-** | **-** | **-** | **-** | **33%** | **49%** | **50%** | **0%** | **0%** |
| *Ward 03* | - | - | - | 4% | - | - | - | - | - | - | - | - | - | - | 4% | 5% | 6% | 0% | 0% |
| *Ward 27* | - | - | - | 4% | - | - | - | - | - | - | - | - | - | - | 4% | 6% | 6% | 0% | 0% |
| *Ward 28* | - | - | - | 8% | - | - | - | - | - | - | - | - | - | - | 8% | 12% | 12% | 0% | 0% |
| *Ward 02* | - | - | - | 3% | - | - | - | - | - | - | - | - | - | - | 3% | 4% | 4% | 0% | 0% |
| *Ward 04* | - | - | - | 3% | - | - | - | - | - | - | - | - | - | - | 3% | 4% | 4% | 0% | 0% |
| *Ward 05* | - | - | - | 2% | - | - | - | - | - | - | - | - | - | - | 2% | 2% | 3% | 0% | 0% |
| *Ward 08* | - | - | - | 2% | - | - | - | - | - | - | - | - | - | - | 2% | 3% | 4% | 0% | 0% |
| *Ward 09* | - | - | - | 3% | - | - | - | - | - | - | - | - | - | - | 3% | 4% | 4% | 0% | 0% |
| *Ward 10* | - | - | - | 3% | - | - | - | - | - | - | - | - | - | - | 3% | 4% | 4% | 0% | 0% |
| *Ward 20* | - | - | - | 2% | - | - | - | - | - | - | - | - | - | - | 2% | 3% | 3% | 0% | 0% |
| *Abbreviations: KG, Kindergarden, YW, Young Women, ZCC, Zion Christian Church. NB. Key Population includes Female Sex Workers and Men who have Sex with Men. § Significantly lower than the other 2 countries, ^ Significantly higher than the other 2 countries. † low base size.* | | | | | | | | | | | | | | | | | | | |

**Table S4. (PEP Experienced Users only): Recall of specific services offered within the PEP pathway.**

|  |  |  | **USERS** | **COUNTRY** | | | **KENYA** | | | | | **NIGERIA** | | | | | **ZIMBABWE** | | | | |
| --- | --- | --- | --- | --- | --- | --- | --- | --- | --- | --- | --- | --- | --- | --- | --- | --- | --- | --- | --- | --- | --- |
|  |  |  | **TOTAL** | **Kenya** | **Nigeria** | **Zimbabwe** | **TOTAL** | **YW** | **Men** | **MSM** | **FSW** | **TOTAL** | **YW** | **Men** | **MSM** | **FSW** | **TOTAL** | **YW** | **Men** | **MSM** | **FSW** |
| **From PEP journey profile:** |  | **Base** | **304** | **145** | **39†** | **120** | **145** | **46†** | **47†** | **26†** | **26†** | **39†** | **12†** | **16†** | **6†** | **5†** | **120** | **40†** | **41†** | **20†** | **19†** |
| **Showcard 3: Assessment** | **Assessment of exposure.** | Yes | ***94.7%*** | *95.2%* | *94.9%* | *94.2%* | ***95.2%*** | *97.8%* | *91.5%* | *96.2%* | *96.2%* | ***94.9%*** | *100.0%* | *93.8%* | *83.3%* | *100.0%* | ***94.2%*** | *92.5%* | *92.7%* | *100.0%* | *94.7%* |
|  | **Assessment of exposure.** | No, it was not offered | ***2.0%*** | *2.8%* | *-* | *1.7%* | ***2.8%*** | *2.2%* | *4.3%* | *3.8%* | *-* | ***-*** | *-* | *-* | *-* | *-* | ***1.7%*** | *2.5%* | *2.4%* | *-* | *-* |
|  | **Assessment of exposure.** | No, I chose not to | ***2.6%*** | *2.1%* | *5.1%* | *2.5%* | ***2.1%*** | *-* | *4.3%* | *-* | *3.8%* | ***5.1%*** | *-* | *6.2%* | *16.7%* | *-* | ***2.5%*** | *2.5%* | *2.4%* | *-* | *5.3%* |
|  | **Assessment of exposure.** | I do not remember | ***0.7%*** | *-* | *-* | *1.7%* | ***-*** | *-* | *-* | *-* | *-* | ***-*** | *-* | *-* | *-* | *-* | ***1.7%*** | *2.5%* | *2.4%* | *-* | *-* |
|  | **Health check** | Yes | ***87.8%*** | *87.6%* | *97.4%* | *85.0%* | ***87.6%*** | *93.5%* | *87.2%* | *84.6%* | *80.8%* | ***97.4%*** | *91.7%* | *100.0%* | *100.0%* | *100.0%* | ***85.0%*** | *82.5%* | *87.8%* | *90.0%* | *78.9%* |
|  | **Health check** | No, it was not offered | ***7.9%*** | *9.7%* | *2.6%* | *7.5%* | ***9.7%*** | *2.2%* | *8.5%* | *15.4%* | *19.2%* | ***2.6%*** | *8.3%* | *-* | *-* | *-* | ***7.5%*** | *12.5%* | *4.9%* | *5.0%* | *5.3%* |
|  | **Health check** | No, I chose not to | ***3.6%*** | *2.1%* | *-* | *6.7%* | ***2.1%*** | *4.3%* | *2.1%* | *-* | *-* | ***-*** | *-* | *-* | *-* | *-* | ***6.7%*** | *5.0%* | *4.9%* | *5.0%* | *15.8%* |
|  | **Health check** | I do not remember | ***0.7%*** | *0.7%* | *-* | *0.8%* | ***0.7%*** | *-* | *2.1%* | *-* | *-* | ***-*** | *-* | *-* | *-* | *-* | ***0.8%*** | *-* | *2.4%* | *-* | *-* |
|  | **HIV test** | Yes | ***98.4%*** | *97.2%* | *97.4%* | *100.0%* | ***97.2%*** | *95.7%* | *97.9%* | *100.0%* | *96.2%* | ***97.4%*** | *91.7%* | *100.0%* | *100.0%* | *100.0%* | ***100.0%*** | *100.0%* | *100.0%* | *100.0%* | *100.0%* |
|  | **HIV test** | No, it was not offered | ***1.3%*** | *2.1%* | *2.6%* | *-* | ***2.1%*** | *4.3%* | *2.1%* | *-* | *-* | ***2.6%*** | *8.3%* | *-* | *-* | *-* | ***-*** | *-* | *-* | *-* | *-* |
|  | **HIV test** | No, I chose not to | ***0.3%*** | *0.7%* | *-* | *-* | ***0.7%*** | *-* | *-* | *-* | *3.8%* | ***-*** | *-* | *-* | *-* | *-* | ***-*** | *-* | *-* | *-* | *-* |
|  | **HIV test** | I do not remember | ***-*** | *-* | *-* | *-* | ***-*** | *-* | *-* | *-* | *-* | ***-*** | *-* | *-* | *-* | *-* | ***-*** | *-* | *-* | *-* | *-* |
|  | **Provision of first aid in case of broken skin or other wound** | Yes | ***48.7%*** | *60.7%^* | *30.8%* | *40.0%* | ***60.7%*** | *60.9%* | *57.4%* | *73.1%* | *53.8%* | ***30.8%*** | *33.3%* | *25.0%* | *50.0%* | *20.0%* | ***40.0%*** | *30.0%* | *26.8%* | *65.0%* | *63.2%* |
|  | **Provision of first aid in case of broken skin or other wound** | No, it was not offered | ***11.2%*** | *12.4%* | *23.1%* | *5.8%* | ***12.4%*** | *13.0%* | *12.8%* | *-* | *23.1%* | ***23.1%*** | *25.0%* | *12.5%* | *50.0%* | *20.0%* | ***5.8%*** | *7.5%* | *4.9%* | *5.0%* | *5.3%* |
|  | **Provision of first aid in case of broken skin or other wound** | No, I chose not to | ***4.3%*** | *2.8%* | *2.6%* | *6.7%* | ***2.8%*** | *2.2%* | *2.1%* | *7.7%* | *-* | ***2.6%*** | *-* | *6.2%* | *-* | *-* | ***6.7%*** | *-* | *7.3%* | *15.0%* | *10.5%* |
|  | **Provision of first aid in case of broken skin or other wound** | I do not remember | ***2.6%*** | *0.7%* | *7.7%* | *3.3%* | ***0.7%*** | *-* | *2.1%* | *-* | *-* | ***7.7%*** | *-* | *-* | *-* | *60.0%* | ***3.3%*** | *2.5%* | *4.9%* | *-* | *5.3%* |
|  | **Provision of first aid in case of broken skin or other wound** | This was not applicable | ***33.2%*** | *23.4%* | *35.9%* | *44.2%* | ***23.4%*** | *23.9%* | *25.5%* | *19.2%* | *23.1%* | ***35.9%*** | *41.7%* | *56.2%* | *-* | *-* | ***44.2%*** | *60.0%* | *56.1%* | *15.0%* | *15.8%* |
| **From PEP journey profile:  Showcard 3: Counselling and support** | **Risk of HIV discussed** | Yes | ***93.8%*** | *91.7%* | *89.7%* | *97.5%^* | ***91.7%*** | *97.8%* | *95.7%* | *88.5%* | *76.9%* | ***89.7%*** | *100.0%* | *87.5%* | *83.3%* | *80.0%* | ***97.5%*** | *100.0%* | *95.1%* | *95.0%* | *100.0%* |
|  | **Risk of HIV discussed** | No, it was not offered | ***4.3%*** | *6.2%* | *5.1%* | *1.7%* | ***6.2%*** | *2.2%* | *4.3%* | *7.7%* | *15.4%* | ***5.1%*** | *-* | *-* | *16.7%* | *20.0%* | ***1.7%*** | *-* | *4.9%* | *-* | *-* |
|  | **Risk of HIV discussed** | No, I chose not to | ***1.0%*** | *1.4%* | *-* | *0.8%* | ***1.4%*** | *-* | *-* | *3.8%* | *3.8%* | ***-*** | *-* | *-* | *-* | *-* | ***0.8%*** | *-* | *-* | *5.0%* | *-* |
|  | **Risk of HIV discussed** | I do not remember | ***1.0%*** | *0.7%* | *5.1%* | *-* | ***0.7%*** | *-* | *-* | *-* | *3.8%* | ***5.1%*** | *-* | *12.5%* | *-* | *-* | ***-*** | *-* | *-* | *-* | *-* |
|  | **Risk and benefits of HIV PEP explained** | Yes | ***96.1%*** | *96.6%* | *84.6%§* | *99.2%* | ***96.6%*** | *95.7%* | *95.7%* | *96.2%* | *100.0%* | ***84.6%*** | *75.0%* | *87.5%* | *83.3%* | *100.0%* | ***99.2%*** | *100.0%* | *97.6%* | *100.0%* | *100.0%* |
|  | **Risk and benefits of HIV PEP explained** | No, it was not offered | ***3.3%*** | *2.8%* | *12.8%^* | *0.8%* | ***2.8%*** | *4.3%* | *2.1%* | *3.8%* | *-* | ***12.8%*** | *25.0%* | *6.2%* | *16.7%* | *-* | ***0.8%*** | *-* | *2.4%* | *-* | *-* |
|  | **Risk and benefits of HIV PEP explained** | No, I chose not to | ***0.3%*** | *0.7%* | *-* | *-* | ***0.7%*** | *-* | *2.1%* | *-* | *-* | ***-*** | *-* | *-* | *-* | *-* | ***-*** | *-* | *-* | *-* | *-* |
|  | **Risk and benefits of HIV PEP explained** | I do not remember | ***0.3%*** | *-* | *2.6%* | *-* | ***-*** | *-* | *-* | *-* | *-* | ***2.6%*** | *-* | *6.2%* | *-* | *-* | ***-*** | *-* | *-* | *-* | *-* |
|  | **Side effects described** | Yes | ***94.7%*** | *93.8%* | *89.7%* | *97.5%* | ***93.8%*** | *91.3%* | *95.7%* | *96.2%* | *92.3%* | ***89.7%*** | *91.7%* | *93.8%* | *83.3%* | *80.0%* | ***97.5%*** | *97.5%* | *95.1%* | *100.0%* | *100.0%* |
|  | **Side effects described** | No, it was not offered | ***3.9%*** | *4.1%* | *10.3%* | *1.7%* | ***4.1%*** | *6.5%* | *-* | *3.8%* | *7.7%* | ***10.3%*** | *8.3%* | *6.2%* | *16.7%* | *20.0%* | ***1.7%*** | *2.5%* | *2.4%* | *-* | *-* |
|  | **Side effects described** | No, I chose not to | ***0.3%*** | *0.7%* | *-* | *-* | ***0.7%*** | *-* | *2.1%* | *-* | *-* | ***-*** | *-* | *-* | *-* | *-* | ***-*** | *-* | *-* | *-* | *-* |
|  | **Side effects described** | I do not remember | ***1.0%*** | *1.4%* | *-* | *0.8%* | ***1.4%*** | *2.2%* | *2.1%* | *-* | *-* | ***-*** | *-* | *-* | *-* | *-* | ***0.8%*** | *-* | *2.4%* | *-* | *-* |
|  | **Counselling on how to take and keep taking PEP for 28 days** | Yes | ***96.4%*** | *96.6%* | *94.9%* | *96.7%* | ***96.6%*** | *100.0%* | *95.7%* | *96.2%* | *92.3%* | ***94.9%*** | *91.7%* | *93.8%* | *100.0%* | *100.0%* | ***96.7%*** | *95.0%* | *100.0%* | *100.0%* | *89.5%* |
|  | **Counselling on how to take and keep taking PEP for 28 days** | No, it was not offered | ***2.6%*** | *3.4%* | *2.6%* | *1.7%* | ***3.4%*** | *-* | *4.3%* | *3.8%* | *7.7%* | ***2.6%*** | *8.3%* | *-* | *-* | *-* | ***1.7%*** | *-* | *-* | *-* | *10.5%* |
|  | **Counselling on how to take and keep taking PEP for 28 days** | No, I chose not to | ***0.3%*** | *-* | *2.6%* | *-* | ***-*** | *-* | *-* | *-* | *-* | ***2.6%*** | *-* | *6.2%* | *-* | *-* | ***-*** | *-* | *-* | *-* | *-* |
|  | **Counselling on how to take and keep taking PEP for 28 days** | I do not remember | ***0.7%*** | *-* | *-* | *1.7%* | ***-*** | *-* | *-* | *-* | *-* | ***-*** | *-* | *-* | *-* | *-* | ***1.7%*** | *5.0%* | *-* | *-* | *-* |
|  | **Specific support in case of sexual assault** | Yes | ***56.6%*** | *64.8%^* | *43.6%* | *50.8%* | ***64.8%*** | *73.9%* | *61.7%* | *57.7%* | *61.5%* | ***43.6%*** | *58.3%* | *25.0%* | *50.0%* | *60.0%* | ***50.8%*** | *40.0%* | *39.0%* | *75.0%* | *73.7%* |
|  | **Specific support in case of sexual assault** | No, it was not offered | ***6.6%*** | *6.2%* | *15.4%* | *4.2%* | ***6.2%*** | *4.3%* | *6.4%* | *7.7%* | *7.7%* | ***15.4%*** | *-* | *12.5%* | *33.3%* | *40.0%* | ***4.2%*** | *2.5%* | *4.9%* | *5.0%* | *5.3%* |
|  | **Specific support in case of sexual assault** | No, I chose not to | ***4.3%*** | *2.8%* | *7.7%* | *5.0%* | ***2.8%*** | *4.3%* | *-* | *3.8%* | *3.8%* | ***7.7%*** | *-* | *18.8%* | *-* | *-* | ***5.0%*** | *-* | *4.9%* | *10.0%* | *10.5%* |
|  | **Specific support in case of sexual assault** | I do not remember | ***2.0%*** | *1.4%* | *-* | *3.3%* | ***1.4%*** | *2.2%* | *-* | *-* | *3.8%* | ***-*** | *-* | *-* | *-* | *-* | ***3.3%*** | *7.5%* | *2.4%* | *-* | *-* |
|  | **Specific support in case of sexual assault** | This was not applicable | ***30.6%*** | *24.8%* | *33.3%* | *36.7%* | ***24.8%*** | *15.2%* | *31.9%* | *30.8%* | *23.1%* | ***33.3%*** | *41.7%* | *43.8%* | *16.7%* | *-* | ***36.7%*** | *50.0%* | *48.8%* | *10.0%* | *10.5%* |
| **From PEP journey profile:  Showcard 3: Prescription** | **PEP initiated as early as possible following exposure (within 72 hours)** | Yes | ***95.7%*** | *96.6%* | *94.9%* | *95.0%* | ***96.6%*** | *97.8%* | *97.9%* | *96.2%* | *92.3%* | ***94.9%*** | *91.7%* | *100.0%* | *83.3%* | *100.0%* | ***95.0%*** | *95.0%* | *92.7%* | *95.0%* | *100.0%* |
|  | **PEP initiated as early as possible following exposure (within 72 hours)** | No, it was not offered | ***3.3%*** | *3.4%* | *5.1%* | *2.5%* | ***3.4%*** | *2.2%* | *2.1%* | *3.8%* | *7.7%* | ***5.1%*** | *8.3%* | *-* | *16.7%* | *-* | ***2.5%*** | *2.5%* | *2.4%* | *5.0%* | *-* |
|  | **PEP initiated as early as possible following exposure (within 72 hours)** | No, I chose not to | ***-*** | *-* | *-* | *-* | ***-*** | *-* | *-* | *-* | *-* | ***-*** | *-* | *-* | *-* | *-* | ***-*** | *-* | *-* | *-* | *-* |
|  | **PEP initiated as early as possible following exposure (within 72 hours)** | I do not remember | ***1.0%*** | *-* | *-* | *2.5%* | ***-*** | *-* | *-* | *-* | *-* | ***-*** | *-* | *-* | *-* | *-* | ***2.5%*** | *2.5%* | *4.9%* | *-* | *-* |
|  | **28-day prescription** | Yes | ***97.7%*** | *100.0%* | *87.2%§* | *98.3%* | ***100.0%*** | *100.0%* | *100.0%* | *100.0%* | *100.0%* | ***87.2%*** | *75.0%* | *87.5%* | *100.0%* | *100.0%* | ***98.3%*** | *97.5%* | *97.6%* | *100.0%* | *100.0%* |
|  | **28-day prescription** | No, it was not offered | ***1.0%*** | *-* | *7.7%§* | *-* | ***-*** | *-* | *-* | *-* | *-* | ***7.7%*** | *25.0%* | *-* | *-* | *-* | ***-*** | *-* | *-* | *-* | *-* |
|  | **28-day prescription** | No, I chose not to | ***0.7%*** | *-* | *5.1%§* | *-* | ***-*** | *-* | *-* | *-* | *-* | ***5.1%*** | *-* | *12.5%* | *-* | *-* | ***-*** | *-* | *-* | *-* | *-* |
|  | **28-day prescription** | I do not remember | ***0.7%*** | *-* | *-* | *1.7%* | ***-*** | *-* | *-* | *-* | *-* | ***-*** | *-* | *-* | *-* | *-* | ***1.7%*** | *2.5%* | *2.4%* | *-* | *-* |
|  | **Drug information given** | Yes | ***92.4%*** | *90.3%* | *97.4%* | *93.3%* | ***90.3%*** | *95.7%* | *87.2%* | *88.5%* | *88.5%* | ***97.4%*** | *91.7%* | *100.0%* | *100.0%* | *100.0%* | ***93.3%*** | *90.0%* | *92.7%* | *95.0%* | *100.0%* |
|  | **Drug information given** | No, it was not offered | ***4.6%*** | *6.9%* | *2.6%* | *2.5%* | ***6.9%*** | *4.3%* | *6.4%* | *7.7%* | *11.5%* | ***2.6%*** | *8.3%* | *-* | *-* | *-* | ***2.5%*** | *5.0%* | *-* | *5.0%* | *-* |
|  | **Drug information given** | No, I chose not to | ***0.7%*** | *-* | *-* | *1.7%* | ***-*** | *-* | *-* | *-* | *-* | ***-*** | *-* | *-* | *-* | *-* | ***1.7%*** | *2.5%* | *2.4%* | *-* | *-* |
|  | **Drug information given** | I do not remember | ***2.3%*** | *2.8%* | *-* | *2.5%* | ***2.8%*** | *-* | *6.4%* | *3.8%* | *-* | ***-*** | *-* | *-* | *-* | *-* | ***2.5%*** | *2.5%* | *4.9%* | *-* | *-* |
|  | **Assessment of health conditions and any other medications** | Yes | ***81.9%*** | *83.4%* | *84.6%* | *79.2%* | ***83.4%*** | *76.1%* | *89.4%* | *84.6%* | *84.6%* | ***84.6%*** | *75.0%* | *81.2%* | *100.0%* | *100.0%* | ***79.2%*** | *80.0%* | *73.2%* | *90.0%* | *78.9%* |
|  | **Assessment of health conditions and any other medications** | No, it was not offered | ***10.5%*** | *12.4%* | *10.3%* | *8.3%* | ***12.4%*** | *19.6%* | *6.4%* | *7.7%* | *15.4%* | ***10.3%*** | *16.7%* | *12.5%* | *-* | *-* | ***8.3%*** | *7.5%* | *7.3%* | *5.0%* | *15.8%* |
|  | **Assessment of health conditions and any other medications** | No, I chose not to | ***3.9%*** | *2.8%* | *5.1%* | *5.0%* | ***2.8%*** | *4.3%* | *4.3%* | *-* | *-* | ***5.1%*** | *8.3%* | *6.2%* | *-* | *-* | ***5.0%*** | *2.5%* | *7.3%* | *5.0%* | *5.3%* |
|  | **Assessment of health conditions and any other medications** | I do not remember | ***3.6%*** | *1.4%* | *-* | *7.5%* | ***1.4%*** | *-* | *-* | *7.7%* | *-* | ***-*** | *-* | *-* | *-* | *-* | ***7.5%*** | *10.0%* | *12.2%* | *-* | *-* |
| **From PEP journey profile:  Showcard 3: Follow-up** | **HIV test at 3 months after exposure** | Yes | ***87.2%*** | *87.6%* | *71.8%§* | *91.7%* | ***87.6%*** | *84.8%* | *89.4%* | *96.2%* | *80.8%* | ***71.8%*** | *58.3%* | *81.2%* | *66.7%* | *80.0%* | ***91.7%*** | *87.5%* | *90.2%* | *100.0%* | *94.7%* |
|  | **HIV test at 3 months after exposure** | No, it was not offered | ***5.3%*** | *6.9%* | *7.7%* | *2.5%* | ***6.9%*** | *8.7%* | *4.3%* | *3.8%* | *11.5%* | ***7.7%*** | *-* | *-* | *33.3%* | *20.0%* | ***2.5%*** | *2.5%* | *4.9%* | *-* | *-* |
|  | **HIV test at 3 months after exposure** | No, I chose not to | ***6.9%*** | *4.8%* | *20.5%^* | *5.0%* | ***4.8%*** | *6.5%* | *4.3%* | *-* | *7.7%* | ***20.5%*** | *41.7%* | *18.8%* | *-* | *-* | ***5.0%*** | *7.5%* | *4.9%* | *-* | *5.3%* |
|  | **HIV test at 3 months after exposure** | I do not remember | ***0.7%*** | *0.7%* | *-* | *0.8%* | ***0.7%*** | *-* | *2.1%* | *-* | *-* | ***-*** | *-* | *-* | *-* | *-* | ***0.8%*** | *2.5%* | *-* | *-* | *-* |
|  | **Link to HIV treatment if needed** | Yes | ***68.4%*** | *71.0%* | *41%§* | *74.2%* | ***71.0%*** | *76.1%* | *72.3%* | *61.5%* | *69.2%* | ***41.0%*** | *33.3%* | *31.2%* | *50.0%* | *80.0%* | ***74.2%*** | *67.5%* | *70.7%* | *80.0%* | *89.5%* |
|  | **Link to HIV treatment if needed** | No, it was not offered | ***8.9%*** | *8.3%* | *20.5%^* | *5.8%* | ***8.3%*** | *2.2%* | *12.8%* | *7.7%* | *11.5%* | ***20.5%*** | *25.0%* | *18.8%* | *16.7%* | *20.0%* | ***5.8%*** | *5.0%* | *4.9%* | *10.0%* | *5.3%* |
|  | **Link to HIV treatment if needed** | No, I chose not to | ***2.6%*** | *3.4%* | *7.7%* | *-* | ***3.4%*** | *4.3%* | *-* | *-* | *11.5%* | ***7.7%*** | *-* | *12.5%* | *16.7%* | *-* | ***-*** | *-* | *-* | *-* | *-* |
|  | **Link to HIV treatment if needed** | I do not remember | ***2.6%*** | *2.8%* | *2.6%* | *2.5%* | ***2.8%*** | *2.2%* | *2.1%* | *7.7%* | *-* | ***2.6%*** | *8.3%* | *-* | *-* | *-* | ***2.5%*** | *5.0%* | *2.4%* | *-* | *-* |
|  | **Link to HIV treatment if needed** | This was not applicable | ***17.4%*** | *14.5%* | *28.2%* | *17.5%* | ***14.5%*** | *15.2%* | *12.8%* | *23.1%* | *7.7%* | ***28.2%*** | *33.3%* | *37.5%* | *16.7%* | *-* | ***17.5%*** | *22.5%* | *22.0%* | *10.0%* | *5.3%* |
|  | **Any other HIV prevention provided if needed (e.g. PrEP)** | Yes | ***81.6%*** | *89.0%* | *51.3%§* | *82.5%* | ***89.0%*** | *80.4%* | *91.5%* | *96.2%* | *92.3%* | ***51.3%*** | *58.3%* | *56.2%* | *33.3%* | *40.0%* | ***82.5%*** | *72.5%* | *80.5%* | *100.0%* | *89.5%* |
|  | **Any other HIV prevention provided if needed (e.g. PrEP)** | No, it was not offered | ***6.6%*** | *3.4%* | *25.6%^* | *4.2%* | ***3.4%*** | *6.5%* | *2.1%* | *3.8%* | *-* | ***25.6%*** | *25.0%* | *18.8%* | *50.0%* | *20.0%* | ***4.2%*** | *7.5%* | *2.4%* | *-* | *5.3%* |
|  | **Any other HIV prevention provided if needed (e.g. PrEP)** | No, I chose not to | ***2.3%*** | *2.1%* | *-* | *3.3%* | ***2.1%*** | *4.3%* | *-* | *-* | *3.8%* | ***-*** | *-* | *-* | *-* | *-* | ***3.3%*** | *5.0%* | *4.9%* | *-* | *-* |
|  | **Any other HIV prevention provided if needed (e.g. PrEP)** | I do not remember | ***3.3%*** | *2.1%* | *7.7%* | *3.3%* | ***2.1%*** | *4.3%* | *2.1%* | *-* | *-* | ***7.7%*** | *-* | *6.2%* | *-* | *40.0%* | ***3.3%*** | *5.0%* | *4.9%* | *-* | *-* |
|  | **Any other HIV prevention provided if needed (e.g. PrEP)** | This was not applicable | ***6.2%*** | *3.4%* | *15.4%^* | *6.7%* | ***3.4%*** | *4.3%* | *4.3%* | *-* | *3.8%* | ***15.4%*** | *16.7%* | *18.8%* | *16.7%* | *-* | ***6.7%*** | *10.0%* | *7.3%* | *-* | *5.3%* |
| *Abbreviations: HIV, Human Immunodeficiency Disease, PEP, Post-Exposure Prophylaxis; PrEP, Pre-Exposure Prophylaxis; YW, Young Women. NB. Key Population includes Female Sex Workers and Men who have Sex with Men. § Significantly lower than the other 2 countries, ^ Significantly higher than the other 2 countries. † Low base size.* | | | | | | | | | | | | | | | | | | | | | |
